# Evidence that the loss of colonic anti-microbial peptides may promote dysbiotic Gram-negative inflammaging-associated bacteria in aging mice

**DOI:** 10.3389/fragi.2024.1352299

**Published:** 2024-03-04

**Authors:** Christopher B. Forsyth, Maliha Shaikh, Phillip A. Engen, Fabian Preuss, Ankur Naqib, Breanna A. Palmen, Stefan J. Green, Lijuan Zhang, Zlata R. Bogin, Kristi Lawrence, Deepak Sharma, Garth R. Swanson, Faraz Bishehsari, Robin M. Voigt, Ali Keshavarzian

**Affiliations:** ^1^ Department of Internal Medicine, Rush University Medical Center, Chicago, IL, United States; ^2^ Rush Center for Integrated Microbiome and Chronobiology Research, Rush University Medical Center, Chicago, IL, United States; ^3^ Department of Anatomy and Cell Biology, Rush University Medical Center, Chicago, IL, United States; ^4^ Department of Biological Sciences, University of Wisconsin Parkside, Kenosha, WI, United States; ^5^ Genomics and Microbiome Core Facility, Rush University Medical Center, Chicago, IL, United States; ^6^ Department of Physiology, Rush University Medical Center, Chicago, IL, United States

**Keywords:** aging, tight junction, microbiota, gene expression, anti-microbial peptides, dysbiosis, inflammaging

## Abstract

**Introduction:** Aging studies in humans and mice have played a key role in understanding the intestinal microbiome and an increased abundance of “inflammaging” Gram-negative (Gn) bacteria. The mechanisms underlying this inflammatory profile in the aging microbiome are unknown. We tested the hypothesis that an aging-related decrease in colonic crypt epithelial cell anti-microbial peptide (AMP) gene expression could promote colonic microbiome inflammatory Gn dysbiosis and inflammaging.

**Methods:** As a model of aging, C57BL/6J mice fecal (colonic) microbiota (16S) and isolated colonic crypt epithelial cell gene expression (RNA-seq) were assessed at 2 months (mth) (human: 18 years old; yo), 15 mth (human: 50 yo), and 25 mth (human: 84 yo). Informatics examined aging-related microbial compositions, differential colonic crypt epithelial cell gene expressions, and correlations between colonic bacteria and colonic crypt epithelial cell gene expressions.

**Results:** Fecal microbiota exhibited significantly increased relative abundances of pro-inflammatory Gn bacteria with aging. Colonic crypt epithelial cell gene expression analysis showed significant age-related downregulation of key AMP genes that repress the growth of Gn bacteria. The aging-related decrease in AMP gene expressions is significantly correlated with an increased abundance in Gn bacteria (dysbiosis), loss of colonic barrier gene expression, and senescence- and inflammation-related gene expression.

**Conclusion:** This study supports the proposed model that aging-related loss of colonic crypt epithelial cell AMP gene expression promotes increased relative abundances of Gn inflammaging-associated bacteria and gene expression markers of colonic inflammaging. These data may support new targets for aging-related therapies based on intestinal genes and microbiomes.

## Introduction

The population of the world is growing older. By the year 2050, the number of people over 65 will double. By 2050, over 21% of the global population, or two billion people, will be over the age of 60 (LeBrasseur et al., 2015; López-Otín et al., 2023). Therefore, there is a great need to understand mechanisms of “unhealthy” aging to be able to direct new approaches and treatments to promote healthy aging, also called “healthspan” (Campisi et al., 2019; Dugan et al., 2023). The key is that inflammation associated with aging (“inflammaging”) is the predominant risk factor and the driver for most age-associated diseases and conditions that limit healthspan and are the focus of geroscience (Niccoli and Partridge, 2012; Kennedy et al., 2014; Boehme et al., 2023). In recent years, intestinal microbiome research has seen a revolution in our understanding of the microbiome’s role in human health (Cryan et al., 2019a; Boehme et al., 2023). This role has gone from a nuisance and health hazard to a new understanding that a “healthy” diet is associated with a microbiome profile that is anti-inflammatory and promotes human health and healthy immunity (Cryan et al., 2019b; Cryan and Mazmanian, 2022). The disrupted gut microbiome community (so-called “dysbiosis”) appears to be the key factor for inflammaging, and thus, the microbiota community can be used to predict healthy aging and survival. In these microbiome calculations, age is the most important factor (Wilmanski et al., 2021). A widely cited aging mechanisms review updated in *Cell* 2023 (Lopez-Otin et al., 2013; López-Otín et al., 2023) has now added both microbiome *dysbiosis* and *inflammation* (also called “inflammaging”) (Franceschi et al., 2000; Franceschi et al., 2018) as “hallmarks of unhealthy aging.” These two concepts, microbiome dysbiosis and inflammaging, also called “*microb-aging*” (Bosco and Noti, 2021), are the focus of this study. These two factors have become widely associated with age-related disorders that collectively represent the leading cause of disability, frailty, and mortality worldwide (Hayflick, 2021).

Many outstanding aging gut microbiome studies in a wide variety of human subjects across the world, as well as aging animal microbiome models, have been carried out over the last 20+ years (Claesson et al., 2012; Biagi et al., 2016; Kong et al., 2019; Ghosh et al., 2022; Dugan et al., 2023). Human microbiome data and machine learning can now be used to estimate subjects’ ages within 6 years (Galkin et al., 2020). The overall conclusion has become that the aging microbiome in humans, mice, and even *Drosophila* (Clark et al., 2015) and killifish (Smith et al., 2017) becomes pro-inflammatory with increased Gram-negative (Gn) bacteria. Together with increased intestinal permeability (so-called “leaky gut”), this Gn dysbiosis promotes systemic inflammation, which drives unhealthy inflammaging, cell senescence, and aging-related diseases (Franceschi et al., 2018; Cryan et al., 2019a; Furman et al., 2019; Conway J and A Duggal, 2021). However, a classic study on human aging subjects that included centenarians noted that *A key feature of microbial dysbiosis during aging is the loss of protective commensals*, *followed by the overgrowth and colonization of endotoxin-producing [Gn] pathobionts…. But we have learned very little about the mechanisms underlying this so-called ‘dysbiosis’ of aging* (Biagi et al., 2016; Franceschi et al., 2018). Investigating potential mechanisms for the increase in pro-inflammatory Gn intestinal bacteria with aging and related colonic mechanisms of inflammaging are the focus of this study. The colon contains, by far, the greatest number (more than 90%) of the intestinal microbiome (de Vos et al., 2022). The gut colonic epithelium is directly facing these gut microbiota, so the host must rely on several physical and biochemical barriers to restrict pathogens/pathobionts from entering the body. These include immunoglobulin A, the mucus layer, tight junctions, and anti-microbial peptides (AMPs) (Song et al., 2023). The colonic mucus barrier is comprised of a firmly attached inner layer devoid of bacteria and a more loosely attached outer layer that contains large numbers of bacteria and AMPs (Johansson et al., 2008; Blyth et al., 2020; Song et al., 2023).

One critical set of evidence supporting a potential role for Gn bacterial dysbiosis driving aging is a recent series of fecal microbiome transplant (FMT) studies in animal models that support a central, even causative, role for the gut microbiome in aging. Several landmark FMT studies in mice have shown that mice without a microbiome (germ free) live longer, and stool transplants from old mice could promote dysbiosis, increased gut leakiness, systemic interleukin-6 (IL-6), and signs of inflammaging in young mice. The FMT from young mice into old mice can reverse signs of aging, including reduced systemic IL-6, and prolong lifespan (Fransen et al., 2017; Thevaranjan et al., 2017; Bárcena et al., 2019). In addition, the FMT from young mice corrected behavioral deficits in old mice (Boehme et al., 2021). Others have repeated these findings in mouse aging models for stroke, in which stroke outcomes improved with young mice FMT into old mice (Spychala et al., 2018). A recent study in the killifish model of aging showed that the gut microbiome of young killifish could improve the lifespan of old killifish by 41% (Smith et al., 2017). Another recent mouse aging study showed that the restoration of intestinal stem cell telomerase could reverse systemic inflammaging (El Maï et al., 2023). Thus, the aging gut-microbiome can drive systemic inflammaging. Relevant to our study, a recent aging study in *Drosophila* investigated the role of *Drosophila* intestinal AMPs in aging. Overall, they found no major effect of individual AMP deletions on lifespan. However, *Drosophila* lacking seven AMP gene families displayed microbiome dysbiosis and a reduced lifespan (Hanson and Lemaitre, 2023). This supports a model for intestinal AMPs in preventing dysbiosis of aging and that loss of AMPs promotes dysbiosis of aging. Intestinal microbiome dysbiosis and loss of gut barrier function are thus associated with inflammaging in humans, mice, fish, and *Drosophila* (Clark et al., 2015; Buford, 2017).

In this study of the aging intestine (colon) and gut microbiome in C57BL/6 mice, we sought to investigate a potential mechanism to explain the increase in pro-inflammatory Gn colonic bacteria (aging dysbiosis), as well as leaky gut and inflammaging associated with aging Gn dysbiosis. We sought to test the *hypothesis* that aging-related changes in colonic crypt epithelial cell gene expression could be a critical mechanism driving the pro-inflammatory increases (aging dysbiosis, leaky gut, and inflammaging markers) that have been described in virtually every study of the aging human, mouse, killifish, and *Drosophila* aging microbiome models (Clark and Walker, 2018; DeJong et al., 2020). We chose the widely validated C57BL/6 mouse model of aging (Yanai and Endo, 2021) and isolated purified intestinal colonic crypt epithelial cells (no other immune cells or tissue) at three mice aging time points: 2 month (mth) (human 18 years old; yo), 15 mth (human 50 yo), and 25 mth (human 84 yo) (Dutta and Sengupta, 2016). We chose to examine mRNA gene expression with RNA-seq in these colonic crypt epithelial cells using a protocol widely used by our group and others to isolate these colonic crypt epithelial cells for organoid culture (Sato et al., 2011; Forsyth et al., 2017). These colonic crypt epithelial cells have been characterized as containing Lgr5^+^ stem cells, goblet cells, epithelial enterocytes, Reg4^+^ Paneth-like cells, tuft cells, and enteroendocrine cells (Sato et al., 2011; Sasaki et al., 2016; Forsyth et al., 2017). We also assessed stool microbial communities using high-throughput sequencing of 16S ribosomal RNA (rRNA) gene amplicons at each aging time point. We then set out to determine if there were differential correlations in microbiome profiles and colonic crypt epithelial cell gene expression at our three aging time points. Our RNA-seq and microbiome data taken together support a model for aging-associated loss of expression of key AMPs in the colon strongly correlating with increased Gn colonic bacteria with loss of Gram-positive (Gp) bacteria (commensals, “good guys”) and critical correlated changes in other key colonic gene expression inflammaging markers of intestinal hyperpermeability (barrier), senescence, and inflammation.

## Methods and materials

### Animals

C57BL/6J (parental generation originally obtained from The Jackson Laboratory) male mice were born and raised in the conventional facility at the University of Wisconsin-Parkside and housed in a light- and temperature-controlled (12 light: 12 dark) environment. The Institutional Animal Care and Use Committees of UW-Parkside approved all methods. Mice were given food (Teklad^©^ irradiated LM-485 mouse diet) and water *ad libitum*. The mice were weaned, and littermates remained group-housed until euthanasia (up to five animals per cage). Mice referred to as “young” euthanized at 2 months (mth) (human: 18 years old; yo), “middle-aged” mice were 15 mth (human: 50 yo), and “old-aged” mice were 25 mth (human: 84 yo). To avoid seasonal or circadian effects, all animals were born during the same season and euthanized simultaneously at the same time of day (∼9–10 a.m.). Following euthanasia, portions of the colon were collected fresh for the isolation of colonic crypt epithelial cells, some portions were paraffin-embedded and formalin-fixed for histological analysis, other portions were preserved in RNA*later* ^©^ for tissue analysis, and stool pellets (stored at −80°C until analysis) were also collected. A power calculation was performed, which indicated that at least n = 5 mice per group would be sufficient (achieving 80% power if the effect size of the aging is at least 20%); therefore, we moved forward with the analysis after collecting samples (feces and colonic crypt epithelial cells) (van der Lugt et al., 2018).

### Colonic crypt isolation

The colonic crypt isolation was based on the modified protocol (Sato et al., 2011; Forsyth et al., 2017; Tran et al., 2021; Steffens et al., 2022). The colons of mice were washed with ice-cold 1× PBS without Ca^++^ or Mg^++^. The samples were cut longitudinally, washed with cold PBS, cut into smaller pieces, and transferred into a 50-mL conical tube. Samples were washed twice with PBS, then placed in a 2.5 mM EDTA chelating buffer, and placed on a shaker at 4°C for 1 h. The EDTA solution was discarded, and the tissue pieces were washed in 1× PBS. Subsequently, the first fraction was discarded. PBS was added to the tubes and pipetted gently multiple times. Next, the crypts were filtered through a 70-μm cell strainer and saved in a fresh conical tube. This was repeated three more times, adding a fresh PBS fraction each time. Ten percent fetal bovine serum (FBS) was added to the crypt suspension, and the samples were centrifuged for 5 minutes at 300 g. The supernatant was discarded, and the cell pellet was re-suspended in 15 mL of advanced Dulbecco’s Modified Eagle Medium (DMEM). The washing and centrifugation steps were repeated three times to get a clean crypt suspension. The isolated colonic crypt epithelial cells were processed for RNA sequencing and library preparation.

### Immunofluorescent staining

The mouse colon tissue immunofluorescent staining of Zonula occludens-1 (ZO-1; tight junction protein marker) and phosphorylated-H2AX (γH2AX, protein signaling DNA damage marker) was performed on paraffin-embedded, formalin-fixed colon tissue samples, which were cut into 5-μm-thick sections, de-paraffinized, and rehydrated in serial ethanol (100%, 95%, and 70%) followed by distilled water (Forsyth et al., 2017; Dodiya et al., 2020). Heat-induced antigen retrieval was completed by submerging tissue in an EDTA buffer for 4 minutes using a pressure cooker. Slides were blocked with 10% donkey serum (Jackson ImmunoResearch, 017–000-12) overnight, followed by overnight incubation with primary antibodies (anti-rabbit ZO-1: 1:500, Invitrogen #61–7300, and anti-gamma H2AX (phosphor S139–1:200) (Abcam # ab11174). Secondary antibodies diluted at 1:250 (Alexa Fluor donkey anti-rabbit 488 #A-2120 and Alexa Fluor donkey anti-rabbit 488 #A-31572, respectively) were applied for 45 min, followed by washing. Sections were stained with DAPI for 3 minutes and mounted in Sigma Fluoromount Aqueous Mounting Medium #F4680. Immunofluorescence images were acquired using a ZEISS Axio Observer 7 at ×40 magnification; five images per sample were processed. The number of positive H2AX foci per total number of nuclei was counted and scored for quantification. Images were quantified for ZO-1 fluorescence density using ImageJ software.

### DNA extraction and next-generation sequencing

Automated DNA extraction of the mice fecal pellets was performed using a QIAcube Connect instrument with the QIAamp PowerFecal Pro DNA Kit (QIAGEN, Germantown, MD), according to the manufacturer’s instructions. Genomic DNA was polymerase chain reaction (PCR)-amplified with primers targeting the V4 variable region of microbial 16S rRNA genes using a two-stage PCR protocol, as described previously (Naqib et al., 2018). The primers contained 5’ common sequence tags known as Fluidigm common sequences 1 and 2 (CS1 and CS2). Primers CS1_515F and CS2_806R (modified from the primer set employed by the Earth Microbiome Project (EMP; ACACTGACGACATGGTTCTACAGTGTGYCAGCMGCCGCGGTAA and TACGGTAGCAGAGACTTGGTCTCCGGACTACNVGGGTWTCTAAT, respectively—underlined regions represent linker sequences)) were employed for the first stage amplifications. PCRs were performed with 10 μL reaction mixture in 96-well plates using repliQa HiFi ToughMix (Quantabio). The PCR conditions were 98 C for 2 minutes, followed by 28 cycles of 98 C for 10 min, 52 C for 1 minute, and 68°C for 1 minute.

Subsequently, a second PCR amplification was performed with 10 μL reaction mixture in 96-well plates using the same PCR master mix. Each well received a separate primer pair with a unique ten-base barcode obtained from the Access Array Barcode Library for Illumina (Fluidigm, South San Francisco, CA; Item# 100–4876). A measure of 1 μL of PCR product from the first stage of amplification was used as a template for the second stage without cleanup. The cycling conditions were 98 °C for 2 minutes, followed by eight cycles of 98 °C for 10 minutes, 60 °C for 1 minute, and 68°C for 1 minute. Libraries were pooled and sequenced with a 10% PhiX spike-in on an Illumina MiniSeq sequencer employing a mid-output flow cell (2 × 154 paired-end reads). Library preparation, pooling, and sequencing were performed at the Genomics and Microbiome Core Facility (GMCF) at Rush University. Raw sequence data (FASTQ files) were deposited in the National Center for Biotechnology Information (NCBI) Sequence Read Archive (SRA) under the BioProject identifier PRJNA1017444.

### Bioinformatics analysis of amplicon sequences

Microbiome bioinformatics were performed using the software package QIIME2 (version 2021.11) (Bolyen et al., 2019). Raw sequence data were checked for quality using FastQC and merged using PEAR (Zhang et al., 2014). Merged sequences underwent quality filtering using the q2‐demux plugin, followed by denoising using DADA2 (via q2‐dada2) (Callahan et al., 2016). Primer adapter sequences were removed using the Cutadapt algorithm (Martin, 2011). Alpha‐diversity metrics (Shannon index, Simpson’s index, observed features, and Pielou’s evenness) and beta-diversity metrics were calculated using q2‐diversity after samples were rarefied to a depth of 4,800 sequences per sample. Taxonomy was assigned using the q2‐feature‐classifier classify‐sklearn naive Bayes taxonomy classifier against the SILVA 138 99% reference database (Quast et al., 2013; Bokulich et al., 2018). Contaminant removal software, *decontam* (Davis et al., 2018), did not detect any contaminants based on the prevalence of amplicon sequence variants (ASVs) in the reagent negative blank controls using default parameters.

To assess microbial community composition, we conducted a permutational multivariate analysis of variance (PERMANOVA), derived from Aitchison distance, which is a linear measure of sample dissimilarity for compositional data (Gloor et al., 2017), using 9,999 permutations and corrected for multiple testing using the Benjamini–Hochberg (BH) method on ASV counts. Centroid-based non-metric multi-dimensional scaling (NMDS) plots were generated for all metadata groups using the *vegan* package in R. These plots were generated based on ASV counts rarefied at 4,800 sequences. Differentially abundant bacterial phyla and genera between pairwise groups were identified using the compositional-centered log-ratio Kruskal–Wallis (CLR-KW) algorithm. Adjusted *p*-values (i.e., q-values) were generated using the BH method. A random-forest based machine-learning approach using the R implementation of the algorithm (*Boruta* algorithm, “randomForest” package) (Kursa and Rudnicki, 2010), was employed (number of iterations = 1,000) to detect features that are important between the groups. Metagenomic functional pathways from 16S rRNA marker genes were predicted using the Phylogenetic Investigation of Communities by Reconstruction of Unobserved States (PICRUSt2) plugin within the QIIME2 environment (Douglas et al., 2020). Generated pathways were annotated against the MetaCyc metabolic pathway database (Caspi et al., 2020). All the downstream data processing (i.e., alpha-diversity, beta-diversity, PERMANOVA, and PICRUSt2) was performed using open-source packages within the R programming language. Bacterial genera are classified based on their cell-wall structures as Gram-positive or Gram-negative.

### RNA preparation and RNA-seq quantification

Total RNA was extracted from mice colonic crypts using the RNeasy Plus Mini Kit (QIAGEN; #74136). Additional DNAse treatment of the RNA was performed using a RNase-Free DNase kit (QIAGEN; #79254) according to the manufacturer’s instructions, followed by purification using a RNeasy Mini Cleanup kit (QIAGEN; 74116) implemented on a QIAcube Connect device. Messenger RNA libraries were created using Revelo mRNA-Seq kits (Tecan; 30186621) implemented on a Tecan MagicPrep NGS system. For each sample, 150 ng of total DNAse-free RNA was used as input, and 17 PCR cycles were performed on the instrument. Libraries were evaluated by sequencing on an Illumina MiniSeq instrument and re-pooled and subsequently sequenced on an Illumina NovaSeq 6000 instrument using an S4 flow cell with paired-end 2 × 150 base reads. Library preparation and MiniSeq sequencing were performed at the Rush GMCF. NovaSeq sequencing was performed at the DNA Services Facility at the University of Illinois Urbana-Champaign. Raw sequence data (FASTQ files) were deposited in the National Center for Biotechnology Information (NCBI) Sequence Read Archive (SRA) under the BioProject identifier PRJNA1017446.

### RNA-seq bioinformatics analysis

Raw reads were trimmed to remove TruSeq adapters and bases from the 3′-end with quality scores less than 20 using Cutadapt (Martin, 2011); trimmed reads shorter than 40 bp were discarded. Trimmed reads were aligned to the *Mus musculus* (house mouse) genome assembly GRCm39 (mm39) from the Genome Reference Consortium [GCA_000001635.9 and GCF_000001635.27] using the STAR RNA-seq aligner (Dobin et al., 2013). The expression level of Ensembl genes was quantified using featureCounts (Liao et al., 2014).

Differential expression statistics were computed using *edgeR* (Robinson et al., 2010; McCarthy et al., 2012) on raw expression counts obtained from quantification. Normalized expression was computed as log_2_ CPM (counts per million), including a TMM normalization. Comparisons were made between the different age groups. In all comparisons, adjusted *p*-values (i.e., q-values) were generated using the BH method. Principal component analysis (PCA) plots, heatmaps, and enhanced volcano plots were also generated within the R programming language. An absolute log_2_FC (log_2_ Fold Change) value of 1.5 (log_2_FC > 1.5 and log_2_FC < −1.5) and a *p*-value (<0.05) were used to generate the enhanced volcano plots. Venn diagrams were created to visualize common and differentially expressed genes (DEGs) (https://bioinformatics.psb.ugent.be/webtools/Venn/), both upregulated and downregulated between different age groups (Bardou et al., 2014).

Data-specific gene sets were chosen and mapped against differentially abundant genes that were generated using the *edgeR* algorithm within R. For generating the inflammation-based gene set, four gene sets were extracted from MSigDB (https://www.gsea-msigdb.org/gsea/msigdb) (Liberzon et al., 2015). The four gene sets used were BIOCARTA_INFLAM_PATHWAY, BIOCARTA_LONGEVITY_PATHWAY, GOBP_ACUTE_INFLAMMATORY_RESPONSE, and GOBP_CHRONIC_INFLAMMATORY_RESPONSE. All redundant genes were removed, and a total of 158 unique genes were used to map our data against this curated inflammation database (Supplementary Data Sheet S1). For the aging-related senescence model of the study, 177 genes from GenAge: The Database of Cell Senescence Genes (http://genomics.senescence.info/cells) (Tacutu et al., 2018) were mapped against DEGs from our analysis. The curated databases of anti-microbial peptides and barrier function gene sets were created from extensive research literature searches on PubMed.

### Gene expression with a real-time polymerase chain reaction

Colonic tissue/crypt samples from the colon were processed for RNA isolation according to the manufacturer’s protocol (RNeasy Mini kit, QIAGEN, Hilden, Germany). cDNA was prepared using the High-Capacity cDNA Reverse Transcription kit from the manufacturer (Applied Biosystems, Foster City, CA). The real-time PCR (RT-PCR) was performed on an Applied Biosystems 7500 apparatus using primers (IDT, Coralville, IA) and Fast SYBR Green (Applied Biosystems). The quantitative analysis was calculated from the ΔΔCt values normalized against the GAPDH used as a housekeeping gene. Data means from n = 5 mice for all data. Specific primer sequences are regenerating islet-derived 3 beta (*Reg3ß*) [F: ACT​CCC​TGA​AGA​ATA​TAC​CCT​CC; R: CGC​TAT​TGA​GCA​CAG​ATA​CGA​G], resistin-like molecule beta (RELMß or *Retnlß*) [F: AAG​CCT​ACA​CTG​TGT​TTC​CTT​TT; R: GCT​TCC​TTG​ATC​CTT​TGA​TCC​AC], and glyceraldehyde-3-phosphate dehydrogenase (*GAPDH*) [F: AGG​TCG​GTG​TGA​ACG​GAT​TTG; R: GGG​GTC​GTT​GAT​GGC​AAC​A] (custom primers from Integrated DNA Technologies, Coralville, IA).

### Multivariate analysis of the microbiota and gene expression

Sparse correlation for compositional data (SparCC) (Friedman and Alm, 2012) was used to generate correlations using differentially abundant bacterial Gp and Gn genera with core DEGs that were derived from our four curated databases: anti-microbial peptides, barrier, inflammation, and senescence. *Pseudo p*-values were computed using 100 randomized sets and then corrected using the BH method. Heatmaps indicate significant associations with *p*-values (*p* < 0.05; empty circle) and q-values (q < 0.05; black circle).

Only significantly strong positive and negative microbe–gene correlations (R < −0.5, R > 0.5; q < 0.05) generated from SparCC were exported and visualized as network plots within the open-source platform Cytoscape 3.10.0 (Shannon et al., 2003). Colors for bacterial taxonomy within Cytoscape were assigned based on whether the bacterial taxonomy was Gp or Gn genera. The colors for the genes and gene clusters (Markov cluster algorithm, MCL) (Brohée and van Helden, 2006) were defined using interaction networks in the STRING database v12.0 (https://string-db.org/) (Szklarczyk et al., 2023). All significant gene pathway enrichment categories (e.g., GO, KEGG, Reactome, Monarch, UniPlot, and SMART) were reported from the STRING database v12.0 per aging comparison. Within the Cytoscape network plots, the thickness of the nodes or individual features was calculated using the cytoHubba module within Cytoscape 3.10.0. The nodes within cytoHubba were ranked using the maximal clique centrality (MCC) method (Chin et al., 2014; Thul and Lindskog, 2018).

### Statistical data analysis

Statistical analyses and graphical visualizations were, unless stated otherwise, conducted using GraphPad Prism 10.0 (GraphPad Software, San Diego, California, United States). Sample-size estimations were based upon two-tailed hypotheses, and power was set to 80%. The Shapiro–Wilk test was used to assess whether the data were normally distributed. In the case of a normal distribution, one-way analysis of variance (ANOVA) was used to assess differences between ages. In the case where the data were not normally distributed, the non-parametric Kruskal–Wallis test was used to assess differences between ages. The BH method was used to adjust for multiple comparison tests. For all statistical tests, unless stated otherwise, a *p*-value < 0.05 was considered statistically significant.

## Results

### Impact of aging on the colonic microbiota structure

**FIGURE 1:**
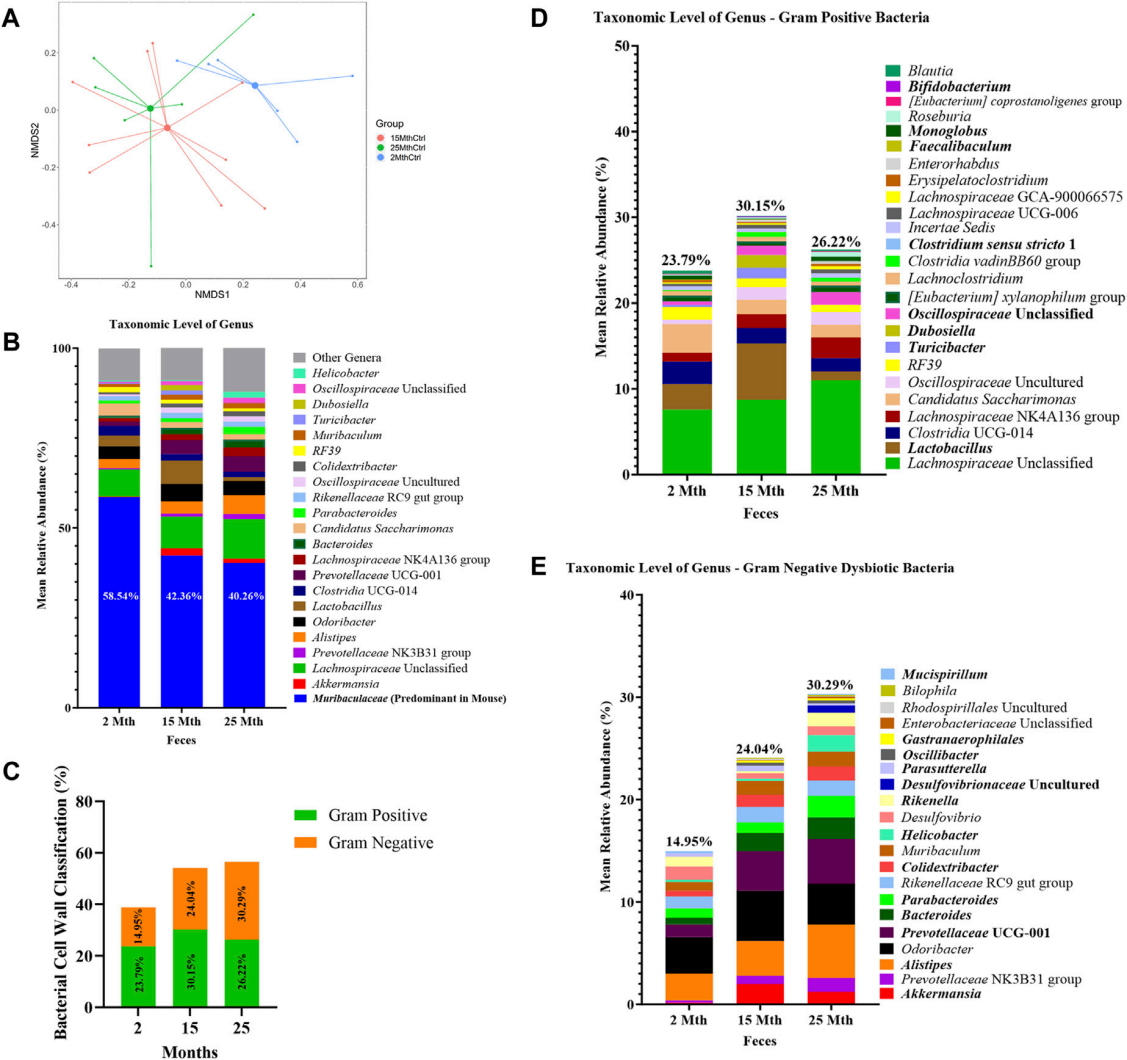
Alteration in gut microbiota composition at different ages. **(A)** Visualization of fecal microbial community structures in 2-mth-, 15-mth-, and 25-mth-old mouse groups was performed using centroid-based NMDS plot-based Aitchison distances using ASV counts rarefied at 4,800 sequences. Symbols representing each mouse fecal sample were connected to a centroid representing the mean value of each aging group: 2 mth (blue), 15 mth (red), and 25 mth (green). Refer to Table 1 for corresponding PERMANOVA data. **(B)** Mean relative abundance of microbial genera (>1% relative abundance) for 2-mth-, 15-mth-, and 25-mth-old mice. **(C)** Bacterial cell wall classifications (%) across time indicating an increased mean relative abundance of dysbiotic Gn genera paired with a reversal decrease in the mean relative abundance of beneficial Gp genera. **(D)** Mean relative abundance of Gp genera (>0.1% relative abundance) and **(E)** Gn dysbiotic genera (>0.1% relative abundance) identifying taxa that are differentially abundant with aging. **(D, E)** Bold taxa indicate a significant difference (*p* < 0.05) between aging mouse groups assessed using the centered log-ratio with Kruskal–Wallis (CLR-KW) to generate *p*-values and corrected for multiple comparisons using the Benjamini–Hochberg method (q-value). Group sample sizes: 2 mth (n = 6), 15 mth (*n* = 9), and 25 mth (n = 7).

Microbial alpha-diversity indices were measured within each mouse’s aged fecal sample to determine whether there were differences in the microbial community structure (Supplementary Table S1; Supplementary Figure S1). No significant differences in alpha-diversity were observed for analyses conducted at the feature level. Although we observed no significant effect of aging on the microbiota alpha-diversity, beta-diversity analyses revealed significant differences in fecal microbial community structures between 2–15-mth-old mice (q = 0.029) and 2–25-mth-old mice (q = 0.037), with respect to Aitchison distance, as the mice samples clustered separately based on their age groups (PERMANOVA: Table 1; centroid-based NMDS plot; Figure 1A).

**TABLE 1:** Intestinal microbial community structure comparisons between mouse aging fecal samples.

Comparison	Feature taxonomic level				
	Sample size	Permutations	Pseudo-F	*p*-value	q-value
2 mth vs. 15 mth	15	9,999	6.147	0.017*	0.029*
2 mth vs. 25 mth	13	9,999	4.939	0.022*	0.037*
15 mth vs. 25 mth	16	9,999	0.607	0.545	0.545

PERMANOVA results are based on the Aitchison distance matrix. The test was performed on feature amplicon sequence variant counts rarefied at 4,800 sequences. PERMANOVA was used to give an insight into the degree of separation between the tested groups of samples. Significance was determined using 9,999 permutations and corrected for multiple testing using the Benjamini–Hochberg method (q-value). Significance (bold*): *p*-value <0.05; q-value <0.05. Group sample sizes: 2 mth (n = 6), 15 mth (n = 9), and 25 mth (n = 7).

Overall, microbial communities between aging mouse groups were different and dominated by bacteria from the phyla Bacteroidetes and Firmicutes (Supplementary Figure S2). Differential abundance analyses indicated increased relative abundances of phyla Cyanobacteria (CLR-KW: 2–15 mth, q = 0.023; 2–25 mth, *p* = 0.007), Verrucomicrobiota (CLR-KW: 2–15 mth, *p* = 0.033), Campylobacterota (2–25 mth, *p* = 0.032; 15–25 mth, *p* = 0.009), and Proteobacteria (CLR-KW: 2–15 mth, *p* = 0.059) in older mice compared to younger mice, along with a decreased relative abundance in the phylum Deferribacterota (CLR-KW: 2–15 mth, q = 0.003; 2–25 mth, *p* = 0.056) in older mice compared to younger mice (Supplementary Data Sheet S2).

At the genus taxonomic level, the relative abundance of *Muribaculaceae* was elevated across all aging mouse groups (2 mth: 58.54% to 25 mth: 40.26%). *Muribaculaceae*, formerly known as S24-7 (phylum Bacteroidetes), is a known dominant bacterium highly abundant in the mouse gut microbiota (Figure 1B) (Lagkouvardos et al., 2019). Beyond the dominant mouse bacterium *Muribaculaceae*, aging mice had diverse microbial environments, similar to those found in the human gut microbiota (Rajilić-Stojanović and de Vos, 2014), composed of Gp and Gn bacterial genera that were significantly altered over time (Figure 1C). The percent bacterial cell-wall classifications across time indicated increased mean relative abundances of pro-inflammatory “dysbiotic” Gn bacteria (2 mth: 14.95% onward to 25 mth: 30.29%), with reversal decreased mean relative abundances of putative beneficial Gp bacteria (15 mth: 30.15% downward to 25 mth: 26.22%) (Figures 1D, E). A total of 10 genera (4 Gp and 6 Gn bacteria: 2-to-15 mth comparison), 11 genera (1 Gp and 10 Gn bacteria: 2-to-25 mth comparison), and 12 genera (7 Gp and 5 Gn bacteria: 15-to-25 mth comparison) were significantly differentially abundant between aging mouse groups (Table 2). The higher relative abundances of Gn dysbiotic genera found in older mice at 15 mth of age were *Oscillibacter*, *Bacteroides*, *Gastranaerophilales*, and *Akkermansia*, as compared to 2-mth-old mice (Table 2; Figure 1E). However, the strongest aging-related Gn dysbiotic genera alterations were shown in 25-mth-old mice with increased relative abundances of *Oscillibacter*, *Bacteroides*, *Parabacteroides*, *Desulfovibrionaceae* unclassified, *Gastranaerophilales*, *Alistipes, Helicobacter*, *Colidextribacter*, and *Prevotellaceae* UCG 001 when compared to 2-mth-old mice (Table 2; Figure 1E). Examining middle-to-late-aged mice, the 25-mth-old mice had a significant loss of Gp genera with decreased relative abundances of *Lactobacillus*, *Turicibacter*, *Dubosiella*, *Faecalibaculum*, *Bifidobacterium*, and *Clostridium* sensu stricto 1 compared to 15-mth-old mice (Table 2; Figure 1D). Inversely, the 25-mth-old mice further had elevated Gn dysbiotic genera with increased relative abundances of *Helicobacter*, *Rikenella*, *Parabacteroides*, and *Desulfovibrionaceae* Unclassified (Table 2; Figure 1E). Additionally, machine learning Boruta feature selections identified these Gp and Gn bacterial genera that were differentially abundant between aging mouse groups, further indicating these genera alterations of importance between aging mouse fecal samples (Table 2).

**TABLE 2:** Machine learning and differential abundance results indicate the genera of importance between mouse aging fecal samples.

Genus taxonomic level	Important feature	2-mth mean RA % ± SD	15-mth mean RA % ± SD	*p*-value	q-value
*Oscillibacter*-Gn	1	0.00 **±** 0.00	0.26 **±** 0.40	0.002*	0.048*
*Rikenella-*Gn	2	0.92 **±** 0.58	0.19 **±** 0.45	0.018*	0.138
*Bacteroides*-Gn	3	0.64 **±** 0.51	1.78 **±** 0.67	0.007*	0.071
*Gastranaerophilales*-Gn	4	0.01 **±** 0.01	0.23 **±** 0.38	0.005*	0.061
*Monoglobus*-Gp	5	0.43 **±** 0.22	0.08 **±** 0.06	0.013*	0.116
*Akkermansia*-Gn	6	0.16 **±** 0.13	1.98 **±** 2.30	0.033*	0.187
*Dubosiella*-Gp	7	0.00 **±** 0.00	1.51 **±** 3.04	0.004*	0.061
*Mucispirillum*-Gn	8	0.20 **±** 0.22	0.00 **±** 0.00	0.0003*	0.018*
*Turicibacter*-Gp	9	0.26 **±** 0.36	1.20 **±** 1.33	0.033*	0.187
*Bifidobacterium*-Gp	10	0.00 **±** 0.00	0.06 **±** 0.15	0.035*	0.187

Genus taxonomic level	Important feature	2-mth mean RA % ± SD	25-mth mean RA % ± SD	*p*-value	q-value
*Oscillospiraceae* Unclassified-Gp	1	0.41 **±** 0.13	1.46 **±** 0.78	0.004*	0.107
*Oscillibacter*-Gn	2	0.00 **±** 0.00	0.30 0.36	0.009*	0.107
*Bacteroides*-Gn	3	0.64 **±** 0.51	2.14 **±** 2.08	0.010*	0.107
*Parabacteroides*-Gn	4	0.92 **±** 0.86	2.09 **±** 0.65	0.018*	0.161
*Desulfovibrionaceae* Uncultured-Gn	5	0.00 **±** 0.00	0.67 **±** 0.94	0.002*	0.085
*Gastranaerophilales*-Gn	6	0.01 **±** 0.01	0.16 **±** 0.18	0.008*	0.107
*Mucispirillum*-Gn	7	0.20 **±** 0.22	0.09 **±** 0.17	0.050*	0.270
*Alistipes*-Gn	8	2.60 **±** 0.97	5.21 **±** 2.34	0.032*	0.213
*Helicobacter*-Gn	9	0.19 **±** 0.19	1.62 **±** 2.27	0.032*	0.213
*Colidextribacter*-Gn	10	0.56 **±** 0.27	1.33 **±** 0.83	0.046*	0.241
*Prevotellaceae* UCG 001-Gn	11	1.24 **±** 0.82	4.33 **±** 3.02	0.046*	0.241

Genus taxonomic level	Important feature	15-mth mean RA % ± SD	25-mth mean RA % ± SD	*p*-value	q-value
*Lactobacillus*-Gp	1	6.55 **±** 7.55	1.00 **±** 0.62	0.003*	0.036*
*Helicobacter*-Gn	2	0.20 **±** 0.26	1.62 **±** 2.27	0.009*	0.064
*Rikenella*-Gn	3	0.19 **±** 0.45	1.35 **±** 0.98	0.002*	0.036*
*Parabacteroides*-Gn	4	0.99 **±** 0.53	2.09 **±** 0.65	0.007*	0.062
*Desulfovibrionaceae* Uncultured-Gn	5	0.00 **±** 0.00	0.67 **±** 0.94	0.0002*	0.013*
*Monoglobus*-Gp	6	0.08 **±** 0.06	0.46 **±** 0.30	0.008*	0.062
*Turicibacter*-Gp	7	1.20 **±** 1.33	0.05 **±** 0.11	0.003*	0.036*
*Dubosiella*-Gp	8	1.51 **±** 3.04	0.00 **±** 0.01	0.003*	0.036*
*Parasutterella*-Gn	9	0.55 **±** 0.31	0.23 **±** 0.14	0.023*	0.119
*Faecalibaculum*-Gp	10	0.07 **±** 0.10	0.00 **±** 0.00	0.023*	0.119
*Bifidobacterium*-Gp	11	0.06 **±** 0.15	0.00 **±** 0.00	0.024*	0.119
*Clostridium sensu stricto* 1-Gp	12	0.04 **±** 0.08	0.00 **±** 0.00	0.048*	0.215

Signature featured genera of importance between mouse aging fecal samples were identified using the machine learning algorithm *Boruta* on unrarefied sequences. Genera (filtered by relative abundances >0.1%) were log-ratio-transformed to detect differential abundance changes using the CLR-KW to generate *p*-values and corrected for multiple comparisons using the Benjamini–Hochberg method (q-value). Significance (bold*): *p*-value <0.05; q-value <0.05. Mean RA %, average number of sequences per taxa, calculated from the total sum of all sequence counts, depicted as a percentage. SD %, standard deviation as a percentage. Gp, Gram-positive; Gn, Gram-negative. Group sample sizes: 2 mth (n = 6), 15 mth (n = 9), and 25 mth (n = 7).

Lastly, we investigated the effects of aging on the metagenome functional content of the fecal microbiota (inferred gene content—PICRUSt2) (Supplementary Data Sheet S2). When compared to the 2-mth-old mice, a total of 16 functional pathways (*p* < 0.05) were increased in 15-mth-old mice and 45 functional pathways (*p* < 0.05) were increased in 25-mth-old mice. Examining mid-to-late-aged mice, five functional pathways were higher (*p* < 0.05) and four functional pathways were lower (*p* < 0.05) in 25-mth-old mice compared to 15-mth-old mice.

Collectively, these data are consistent with intestinal microbiota studies demonstrating the significant aging-related hallmark increase in Gn-associated dysbiotic bacteria in the colon (stool) in 15-mth- and 25-mth-aged mouse groups.

### Effect of aging on colonic crypt epithelial cell gene expression

**FIGURE 2:**
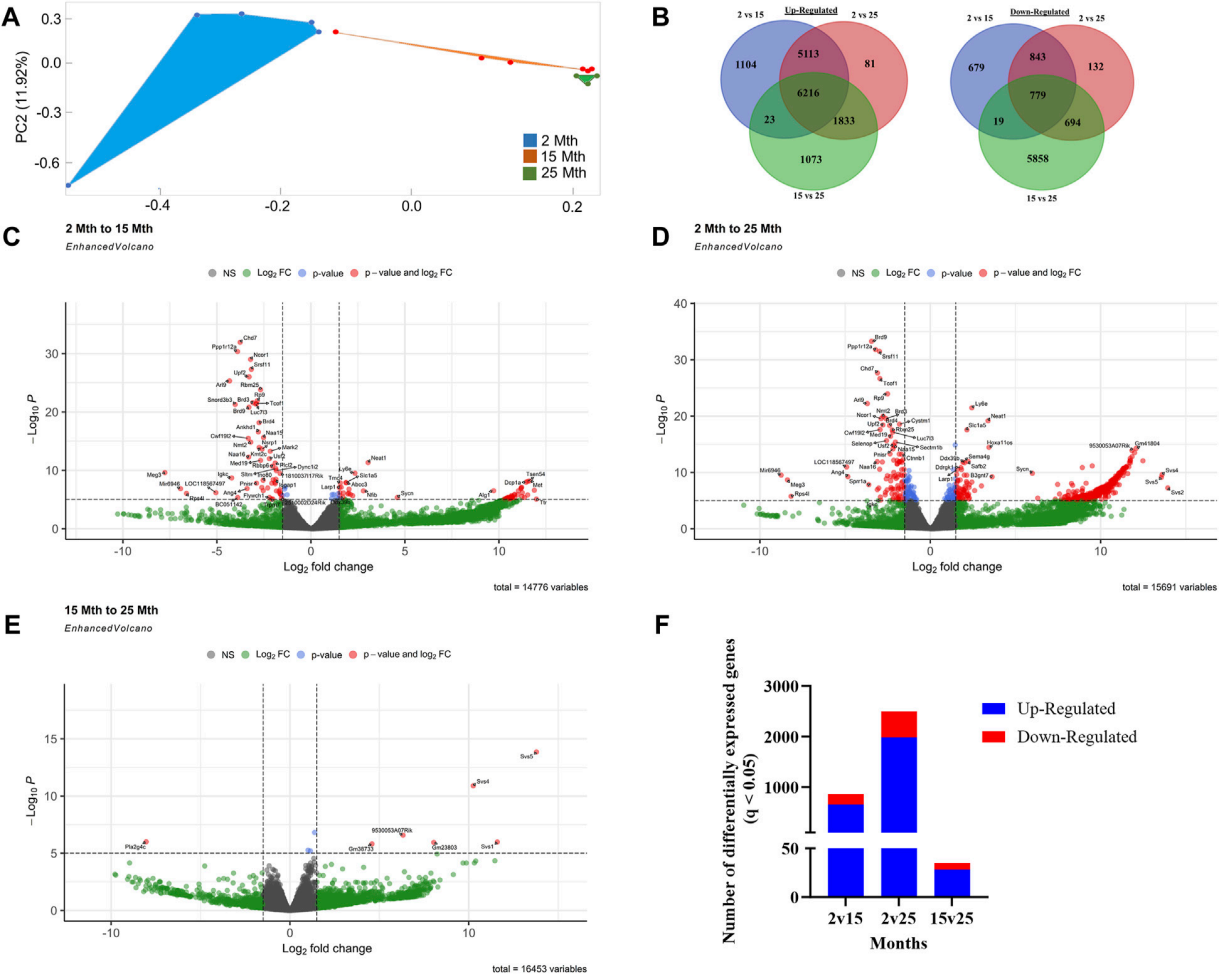
Effects of aging on colonic epithelial crypt cell gene expression. **(A)** PCA plot using 16,729 genes revealed a clear separation between aging mouse groups: 2 mth (blue), 15 mth (red), and 25 mth (green). **(B)** Venn diagrams showing the number of overlapping and unique differentially expressed genes upregulated and downregulated between 2 mth vs. 15 mth, 2 mth vs. 25 mth, and 15 mth vs. 25 mth mouse groups. Enhanced volcano plots for **(C)** 2 mth vs. 15 mth mice, **(D)** 2 mth vs. 25 mth mice, and **(E)** 15 mth vs. 25 mth mice absolute log_2_ fold change (log_2_FC) value of 1.5 (log_2_FC > 1.5 and log_2_FC < −1.5) and *p*-value (<0.05) identified more than a total of 14,000 DEGs for each pairwise comparison: 2 mth vs. 15 mth: 14,776 total DEGs; 2 mth vs 25 mth: 15,691 total DEGs; and 15 mth vs 25 mth: 16,453 total DEGs. **(F)** Total number of DEGs (q < 0.05) both upregulated and downregulated for all age comparisons. Differential expression statistics were computed using *edgeR*. mth, month; NS, not significant. Group sample sizes: 2 mth (n = 5), 15 mth (n = 6), and 25 mth (n = 6).

To explore the impact of aging on gene expression in the colon, RNA-seq analysis was performed on total RNA extracted from colonic crypt epithelial cells from mice, for which we also had fecal samples. A PCA plot was conducted on 16,729 genes that revealed a clear separation between aging mouse groups (Figure 2A). To show the overall distribution of variable genes between each aging mouse group comparison, Venn diagrams were created to depict both upregulated and downregulated overlapping and unique genes that were differentially expressed in the colonic crypt epithelial cells (Figure 2B; Supplementary Data Sheet S3). These isolated cells were processed for RNA-seq analysis, and the resulting data for the three aging mouse time points identified more than a total of 14,000 DEGs for each pairwise comparison (2 vs. 15 mth: 14,776 total DEGs; 2 vs. 25 mth: 15,691 total DEGs; and 15 vs. 25 mth: 16,453 total DEGs) (Figures 2C–E; Supplementary Data Sheets S4–6). The highest number of significantly DEGs (q < 0.05) (2,492 DEGs: 1,978 upregulated and 514 downregulated) were found between the youngest 2-mth-old mice compared to the oldest 25-mth-old mice group (Figure 2F). All significant DEGs (p- and q-values <0.05) for each pairwise aging mouse comparison are shown in Supplementary Data Sheets S4–6.

With the substantial number of DEGs per aging mouse comparison, we sought to focus our analysis on evaluating our hypothesis rather than simply mapping global gene expression; thus, we designed four curated gene sets to analyze in-depth. Curated gene set 1 was assembled based on known colonic AMPs and potential AMPs, like S100 calcium-binding proteins g (*S100g*) and A10 (*S100a10*) and related proteins. Curated gene set 2 was designed on intestinal barrier gene expression, like ZO-1 tight junction protein (*TJP1*). Curated gene sets 3 (senescence) and 4 (inflammation) were compiled to investigate potential inflammaging-related mechanisms for the increase in Gn bacteria we observed in the aging mouse microbiota data.

**FIGURE 3:**
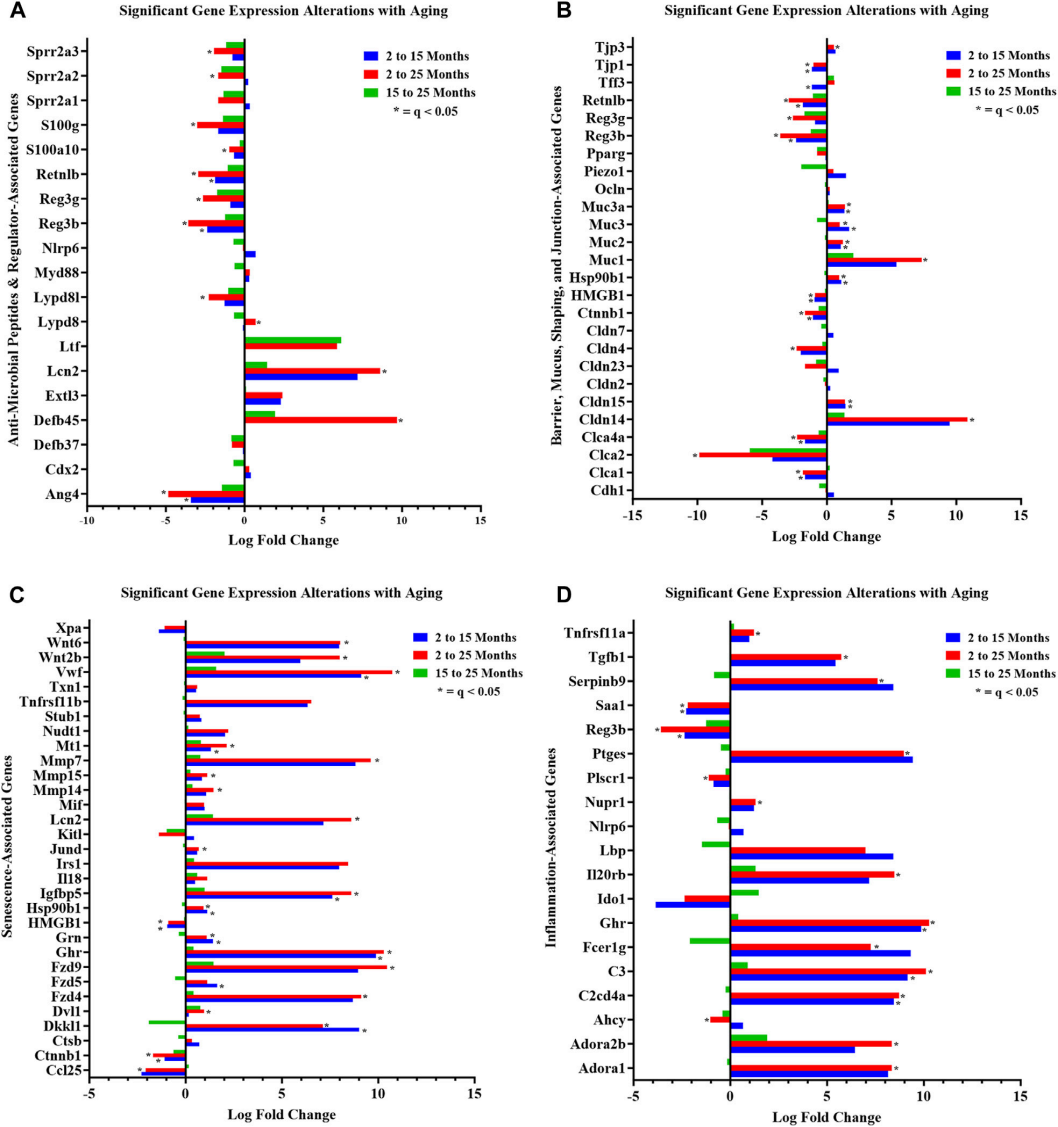
Curated gene sets depicting differentially expressed genes between ages. Significant (q < 0.05) DEG alterations (log_2_ fold change) between aging mouse groups are indicated: **(A)** anti-microbial peptides and regulator-associated DEGs; **(B)** barrier, mucus, shaping, and junction-associated DEGs; **(C)** senescence-associated DEGs; and **(D)** inflammation-associated DEGs. Refer to Supplementary Tables S2, S4–S6 for corresponding statistical data. Differential expression statistics were computed using *edgeR*. Group sample sizes: 2 mth (n = 5), 15 mth (n = 6), and 25 mth (n = 6).

For curated gene set 1, aging strongly affected colonic crypt epithelial cell AMP gene expression in the study. Differential gene expression analysis (2-mth compared to 25-mth-old mice) indicated a significant (q < 0.05) downregulation for five AMP genes, namely, RELMβ (resistin-like molecule beta, *Retnlb*), *Reg3β*, *Reg3γ*, *Ang4*, and *Lypd8l*, as well as several AMP-related protein genes, including *S100g*, *S100a10*, *Sprr2a2*, and *Sprr2a3* in aging mice (Figure 3A; gene descriptions, accession numbers, and data; Supplementary Table S2). Of note, there were three AMP-related protein genes significantly (q < 0.05) upregulated, namely, *Defb45*, *Lypd8*, and *Lcn2*, in aging mice (Figure 3A; Supplementary Table S2). Additionally, the gene expressions of three AMPs, namely, RELMβ (*Retnlb*), *Reg3β*, and *Ang4*, were significantly (q < 0.05) downregulated at the onset of the study in 15-mth-old mice compared to 2-mth-old mice (Figure 3A; Supplementary Table S2).

We noted dramatic downregulated expressions of the AMPs: RELMβ (*Retnlb*) (80%) (q = 7.374E-05) and *Reg3β* (86%) (q = 4.894E-04) in 25-mth-old mice compared to 2-mth-old mice. Additional analysis of AMP (*RELMβ* and *Reg3β*) gene expression loss in aging mice was validated using RT-PCR on colonic tissue/crypt samples (Supplementary Table S3; Supplementary Figure S3). Both AMPs demonstrated an aging effect (RELMβ (*Retnlb*), KW: *p* = 0.0015) (*Reg3β*, one-way ANOVA F = 4.551, *p* = 0.0300), indicating that these gene expressions decreased with aging. Multiple comparison analysis showed the significantly decreased expression of both RELMβ (*Retnlb*) (q = 0.0040) and *Reg3β* (q = 0.0205) in 25-mth-old mice when compared to 2-mth-old mice. These data indicate the potential vital role of the loss of two important colonic AMPs in aging related to dysbiosis and colonic inflammaging.

In curated gene set 2, aging robustly altered colonic crypt epithelial cell barrier-associated gene expressions. The number of significant (q < 0.05) barrier-associated DEGs (n = 13) was evident in the 2-mth-old mice compared to the 15-mth-old mice, but then further significantly (q < 0.05) noticeably differentially expressed (n = 18) in the 2-mth- compared to 25-mth-old mice (Figure 3B; gene descriptions, accession numbers, and data; Supplementary Table S4). Aging mice notably had downregulated barrier-associated expression of genes such as *Ctnnb1*, *Clca1*, *Clca2*, *Clca4a*, *Hmgb1*, RELMβ (*Retnlb*), *Reg3β*, *Tjp1*, *Reg3γ*, and *Cldn4*, along with an upregulated expression of *Cldn14*, *Cldn15*, *Muc1*, *Muc2*, *Muc3*, *Muc3a*, *Hsp90b1*, and *Tjp3* (Figure 3B; Supplementary Table S4).

Furthermore, aging had a pronounced effect on both curated gene sets 3 (senescence) and 4 (inflammation), suggesting that these significant DEGs could potentially be involved in inflammaging-related mechanisms. A total of 25 senescence genes were significantly (q < 0.05) differentially expressed for 2-mth compared to 25-mth-old mice (Figure 3C; gene descriptions, accession numbers, and data; Supplementary Table S5). Aging mice markedly (q < 0.05) exhibited downregulation in the expression of senescence-related genes such as *Ctnnb1*, *Hmgb1*, *Cdkn1a*, *Eps8*, and *Ccl25*, accompanied by an upregulated expression of *Vwf*, *Fzd4*, *Fzd9*, *Mt1*, *Ghr*, *Igfbp5*, *Mmp7*, *Mmp14*, *Mmp15*, *Cav1*, *Hsp90b1*, *Wnt2b*, *Wnt6*, *Jund*, *Grn*, *Dkkl1*, *Lmna*, *Lcn2*, *Dvl1*, and *Polg* (Figure 3C). The fourth gene set indicated there were 21 significant (q < 0.05) inflammation DEGs between 2-mth compared to 25-mth-old mice (Figure 3D; gene descriptions, accession numbers, and data; Supplementary Table S6). Aging mice markedly (q < 0.05) exhibited downregulation in the expression of inflammation-related genes such as *Ahcy*, *Saa1*, *Reg3β*, *Plscr1*, and *Reg3γ* together with an upregulation in the expression of *C3*, *Ghr*, *Ptges*, *C2cd4a*, *Dnase1*, *Nupr1*, *Il20rb*, *Adora1*, *Adora2b*, *Selenos*, *Tgfb1*, *Fcer1g*, *Tnfrsf11a*, *Park7*, *Serpinb9*, and *Tgfb2*.

Together, the investigation of our hypothesis using these four gene set data helped provide a meaningful step forward in attempting to better understand the complex colonic gene expression with aging.

### Tight junction protein zonula occludens-1 integrity in aging mice

The ZO-1 protein plays a key role in intestinal epithelial barrier function. Therefore, we assessed the ZO-1 protein in the colons of aging mice. Immunofluorescence staining for ZO-1 demonstrated a significant (KW: *p* = 0.0005) disruption between aged mouse groups, indicating that its expression is decreased with aging (Supplementary Table S7; Supplementary Figure S4). Multiple comparison analysis showed significantly (q = 0.0008) decreased expression of ZO-1 in 25-mth-old mice when compared to both 2-mth- and 15-mth-old mouse groups. These data serve as the functional validation of our RNA-seq data and support the notion that aging and associated microbiota dysbiosis disrupt intestinal barrier integrity and promote intestinal leak.

### Phosphorylation of the histone variant H2AX in aging mice

Analysis of γH2AX protein expression can be used to detect DNA damage signaling and repair responses. We examined the γH2AX protein in the colons of aging mice as a quantitative marker of DNA double-strand breaks. The γH2AX protein expression demonstrated an aging effect (one-way ANOVA: F (2,11) = 10.04, *p* = 0.0033), indicating that its expression is increased with aging (Supplementary Table S8; Supplementary Figure S5). Multiple comparison analysis showed significantly increased expression of γH2AX in 25-mth-old mice when compared to both 2-mth- (q = 0.0011) and 15-mth-old (q = 0.0136) mouse groups. These data help validate that the effects of aging lead to an increase in inflammatory response and DNA damage signaling in the colon.

### Interactions between gut microbes and colonic crypt epithelial cell-curated gene sets

We used SparCC to generate correlations using bacterial Gp and Gn genera (*p* < 0.05 and filtered at (>1%) mean relative abundance) with core DEGs that were derived from our four curated databases: AMPs, barrier function/integrity, senescence, and inflammation. Only significantly strong positive and negative gene–gene and gene–microbe associations with strong effect sizes (SparCC: R < −0.5, R > 0.5; q < 0.05) were described, exported, and visualized as network plots. Comprehensive SparCC correlation results for the three mice age span comparisons are shown: 2–15 mth, 2–25 mth, and 15–25 mth (Supplementary Data Sheets S7–10). For all four curated gene datasets, thorough integrated network data defined MCL gene colors and gene clusters, interaction scores, significant gene pathway enrichment categories, and MCC method rankings for the two mice age spans (2–15 mth and 2–25 mth) are presented (Supplementary Data Sheets S11–14). Based on these described parameters, the four curated gene datasets and bacterial correlation outcomes are described below.

### Integrative analysis of gut microbes and anti-microbial peptide genes

**FIGURE 4:**
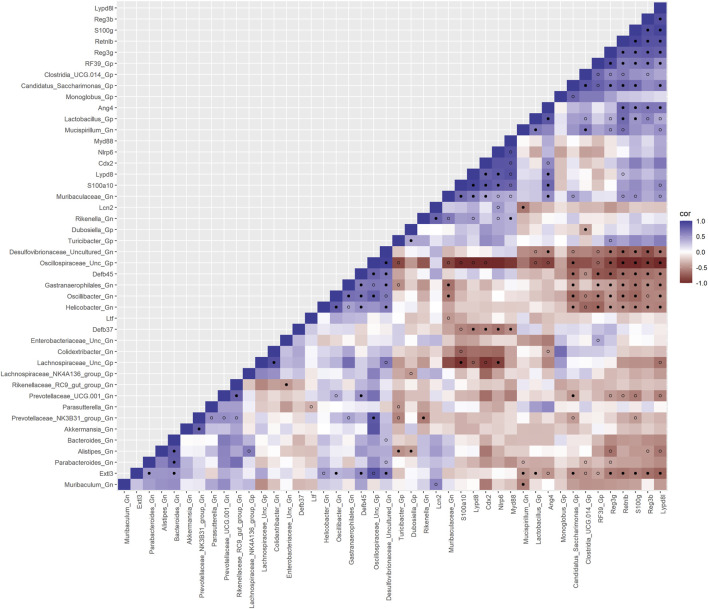
Interactions between aging anti-microbial peptide genes and Gram-positive/Gram-negative gut microbes comparing 2-mth- and 25-mth-old mice. SparCC plot depicting gene–microbe correlations. The colors indicate the magnitude of the correlation. *Pseudo p*-values were computed using 100 randomized sets and then corrected using the Benjamini–Hochberg method. The heatmap indicates significant associations with *p*-values (*p* < 0.05; empty circle) and q-values (q < 0.05; black circle). Gp, Gram-positive genus; Gn, Gram-negative genus.

**FIGURE 5:**
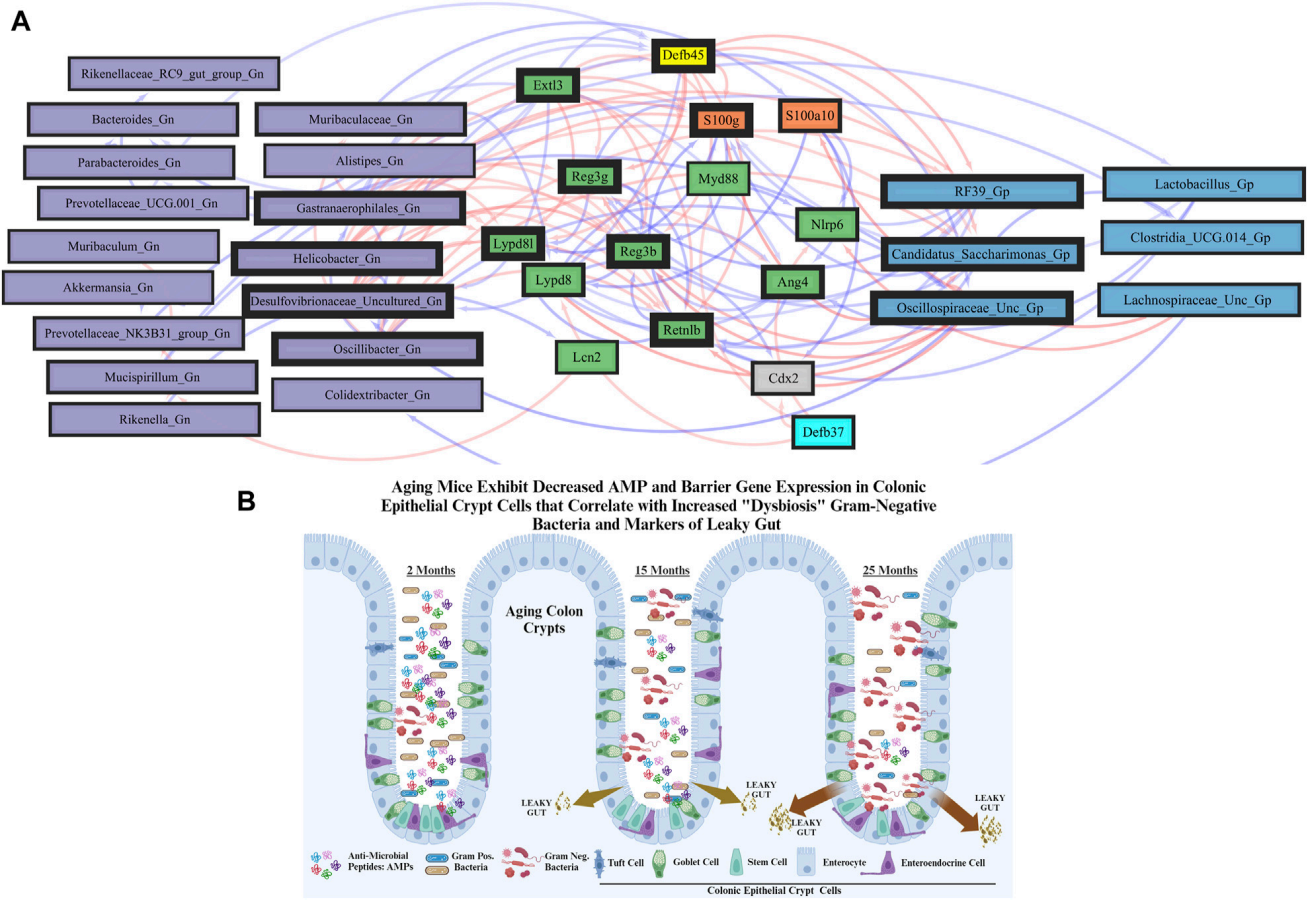
Network interactions depicting significant aging anti-microbial peptide genes and Gram-positive/Gram-negative gut microbes comparing 2-mth- and 25-mth-old mice. **(A)** Significant correlations (R < −0.5, R > 0.5; q < 0.05) generated from SparCC were exported and visualized as network plots within the open-source platform Cytoscape 3.10.0. Blue edges indicate a positive correlation, and red edges indicate a negative correlation. Purple nodes indicate Gram-negative genera, and blue nodes indicate Gram-positive genera. The colors for the genes and gene clusters (based on the Markov cluster algorithm) were defined using interaction networks in the STRING database v12.0. The thickness of the nodes or individual features was calculated using the cytoHubba module within Cytoscape 3.10.0 and ranked by importance (thick black square: higher importance; thin black square: lower importance) using the maximal clique centrality method. **(B)** Schematic model representing aging mice exhibits significant downregulation of both AMP and barrier gene expressions in colonic epithelial crypt cells that correlate with increased pathobiont Gram-negative bacteria and intestinal markers of leaky gut. Created with BioRender.com.

To investigate the relationships between genes and microbes in the colonic crypt epithelial cells for their potential roles in the effects of aging, we highlight the significantly strong correlations between the core 16 AMPs genes and 28 combined bacterial Gp and Gn bacterial genera that were examined for the age span comparison between 2-mth- and 25-mth-old mice (Figure 4; Supplementary Data Sheet S7). In aging, the relationships between these AMP genes (MCC network rankings: RELMβ (*Retnlb*), *S100g*, *Lydp8l*, *Reg3ß*, *Reg3γ*, and *Ang4*) were positively associated with lower expression with each other but negatively associated with higher abundances of signature-dysbiotic Gn genera (MCC rankings: *Desulfovibrionaceae* Unclassified, *Helicobacter*, *Oscillibacter*, and *Gastranaerophilales*)(Figure 5A; Supplementary Data Sheets S7, 11). It is also interesting that the downregulated expression of these six AMP genes positively correlated with lower abundances of Gp genera (MCC network rankings: *Candidatus Saccharimona*, *RF39*, and *Lactobacillus*) over time (Figure 5A; Supplementary Data Sheets S7, 11).

In the early phase of aging between 2-mth- and 15-mth-old mice, these same core AMP genes (MCC network rankings: *Reg3ß*, RELMβ (*Retnlb*), *S100g*, *Ang4*, *Lydp8l*, and *Reg3γ*) were positively associated with downregulated expression with each other while being negatively associated with higher abundances of dysbiotic-associated Gn genera (MCC rankings: *Oscillibacter*, *Gastranaerophilales*, and *Muribaculum*). *Bacteroides and Akkermansia* exhibited increased abundances paired with downregulated RELMβ (*Retnlb*) (Supplementary Figures S6, 7; Supplementary Data Sheets S7, 11). The heatmap (Supplementary Figure S8; Supplementary Data Sheets S7, 11) depicts the significant associations between AMP genes and genera in 15-mth- mice and 25-mth-old mice.

**FIGURE 6:**
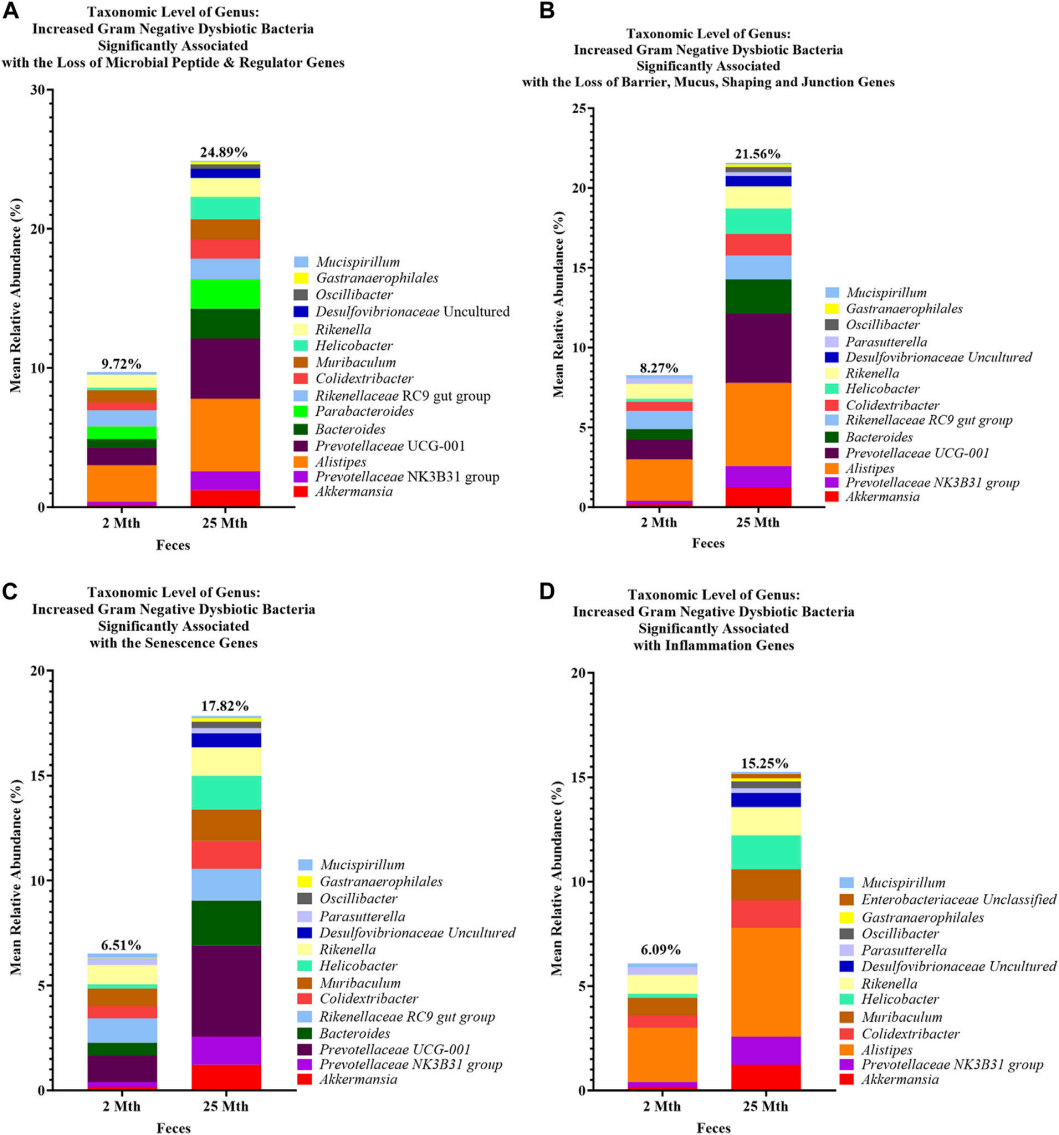
Loss of aging-related hallmark genes increased Gram-negative dysbiotic bacteria between 2-mth- and 25-month-old mice. Mean relative abundance percentages of Gram-negative (Gn) genera that significantly correlated with the expression losses of hallmark genes: **(A)** anti-microbial peptides and regulator genes, **(B)** barrier, mucus, shaping, and junction genes, **(C)** senescence genes, and **(D)** inflammation genes indicated similar or unique increased abundances of dysbiotic and inflammaging Gn bacterial compositions with aging.

These strongly significant correlations suggest an understanding of the complex colonic gene expression and associated microbiome profile mechanisms involved in promoting increased putative pro-inflammatory Gn bacteria associated with the losses of core AMP gene expressions throughout the course of aging (Figures 5B; Figure 6A). The significant gene pathway enrichment categories associated with the loss of AMP genes between 2-mth- and 25-mth-old mice are shown (Table 3) to highlight the potential effects of aging on human health. For significant gene pathway enrichment categories between 2-mth- and 15-mth-old mice, please refer to Supplementary Data Sheet S11.

**TABLE 3:** Top 10 most significant gene pathway enrichment categories of anti-microbial peptides, barrier, senescence, and inflammation between 2-mth- and 25-mth-old mice.

Anti-microbial peptides			
Category ID	Description	Gene count	q-value
GO:0042742	Defense response to bacterium	8	**4.21E-08**
GO:0009617	Response to bacterium	9	**4.80E-07**
GO:0050830	Defense response to Gram-positive bacterium	6	**8.14E-07**
GO:0050829	Defense response to Gram-negative bacterium	4	**0.00052**
GO:0051873	Killing by the host of symbiont cells	3	**0.00057**
GO:0005576	Extracellular region	9	**0.0021**
GO:0005615	Extracellular space	8	**0.0021**
GO:0006955	Immune response	7	**0.0027**
GO:0044278	Cell wall disruption in another organism	2	**0.0075**
GO:0002526	Acute inflammatory response	3	**0.0169**
Barrier			
GO:0043296	Apical junction complex	11	**1.55E-14**
GO:0005923	Bicellular tight junction	10	**1.23E-13**
GO:0045216	Cell–cell junction organization	11	**3.78E-13**
GO:0070830	Bicellular tight junction assembly	8	**5.52E-12**
GO:0007043	Cell–cell junction assembly	9	**1.38E-11**
GO:0016338	Calcium-independent cell–cell adhesion via plasma membrane cell-adhesion molecules	6	**1.02E-09**
GO:0098609	Cell–cell adhesion	10	**4.57E-08**
GO:0016327	Apicolateral plasma membrane	5	**7.62E-08**
GO:0016328	Lateral plasma membrane	6	**8.49E-08**
GO:0070161	Anchoring junction	12	**2.84E-07**
Senescence			
GO:0042221	Response to chemical	33	**1.13E-13**
GO:0050896	Response to stimulus	40	**1.35E-13**
GO:0048523	Negative regulation of the cellular process	33	**1.88E-11**
GO:0051716	Cellular response to stimulus	35	**5.97E-11**
GO:0031399	Regulation of the protein modification process	21	**4.07E-10**
GO:0031401	Positive regulation of the protein modification process	18	**4.07E-10**
GO:0048518	Positive regulation of the biological process	35	**4.07E-10**
GO:0051246	Regulation of the protein metabolic process	25	**4.07E-10**
GO:0051338	Regulation of transferase activity	17	**4.07E-10**
GO:0048522	Positive regulation of the cellular process	33	**1.05E-09**
Inflammation			
GO:0006954	Inflammatory response	17	**1.62E-16**
GO:0002673	Regulation of acute inflammatory response	10	**7.93E-16**
GO:0080134	Regulation of response to stress	21	**9.39E-16**
GO:0032101	Regulation of response to external stimulus	18	**2.58E-14**
GO:0050727	Regulation of inflammatory response	14	**2.58E-14**
GO:0031347	Regulation of defense response	15	**8.49E-13**
GO:0032103	Positive regulation of response to external stimulus	13	**1.25E-11**
GO:0002682	Regulation of the immune system process	18	**1.40E-11**
GO:0048583	Regulation of response to stimulus	24	**5.12E-11**
GO:0002376	Immune system process	19	**2.64E-10**

Top ten significant gene pathway enrichment Gene Ontology (GO) categories (biological process, molecular function, and cellular component) based on Benjamini–Hochberg correction (bold q-value), as reported from the STRING database v12.0 per aging comparison. Please refer to Supplementary Data Sheet S11 (anti-microbial peptides), Supplementary Data Sheet S12 (barrier), Supplementary Data Sheet S13 (senescence) and Supplementary Data Sheet S14 (inflammation) for further details; significant gene pathway enrichment categories beyond the top ten; significant gene enrichment pathway categories between 2-mth-- and 15-mth-old mouse groups.

### Integrative analysis of gut microbes and intestinal barrier function genes

**FIGURE 7:**
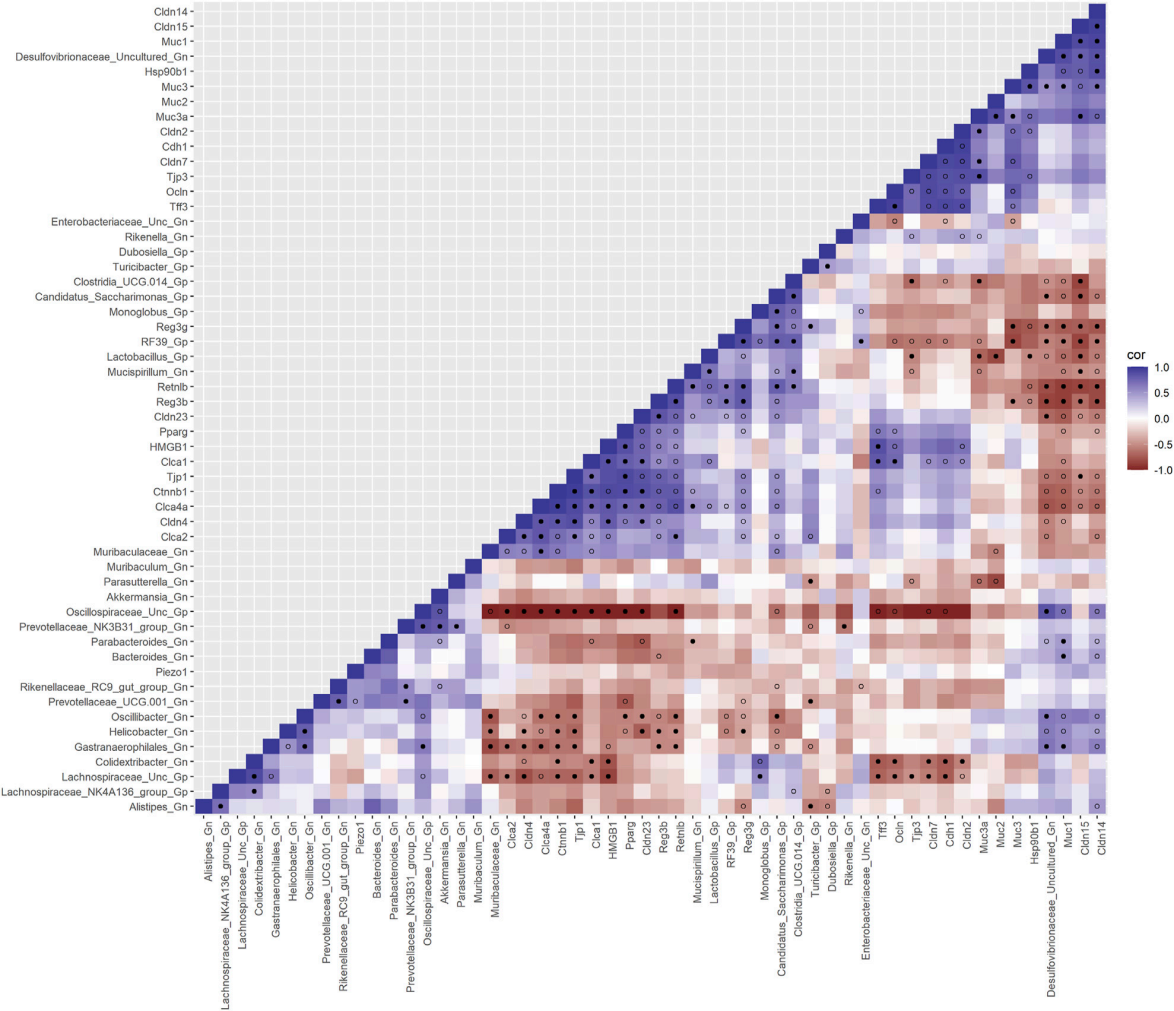
Interactions between aging barrier genes and Gram-positive/Gram-negative gut microbes comparing 2-mth- and 25-month-old mice. SparCC plot depicting gene–microbe correlations. The colors indicate the magnitude of the correlation. *Pseudo p*-values were computed using 100 randomized sets and then corrected using the Benjamini–Hochberg method. The heatmap indicates significant associations with *p*-values (*p* < 0.05; empty circle) and q-values (q < 0.05; black circle). Gp, Gram-positive genus; Gn, Gram-negative genus.

**FIGURE 8:**
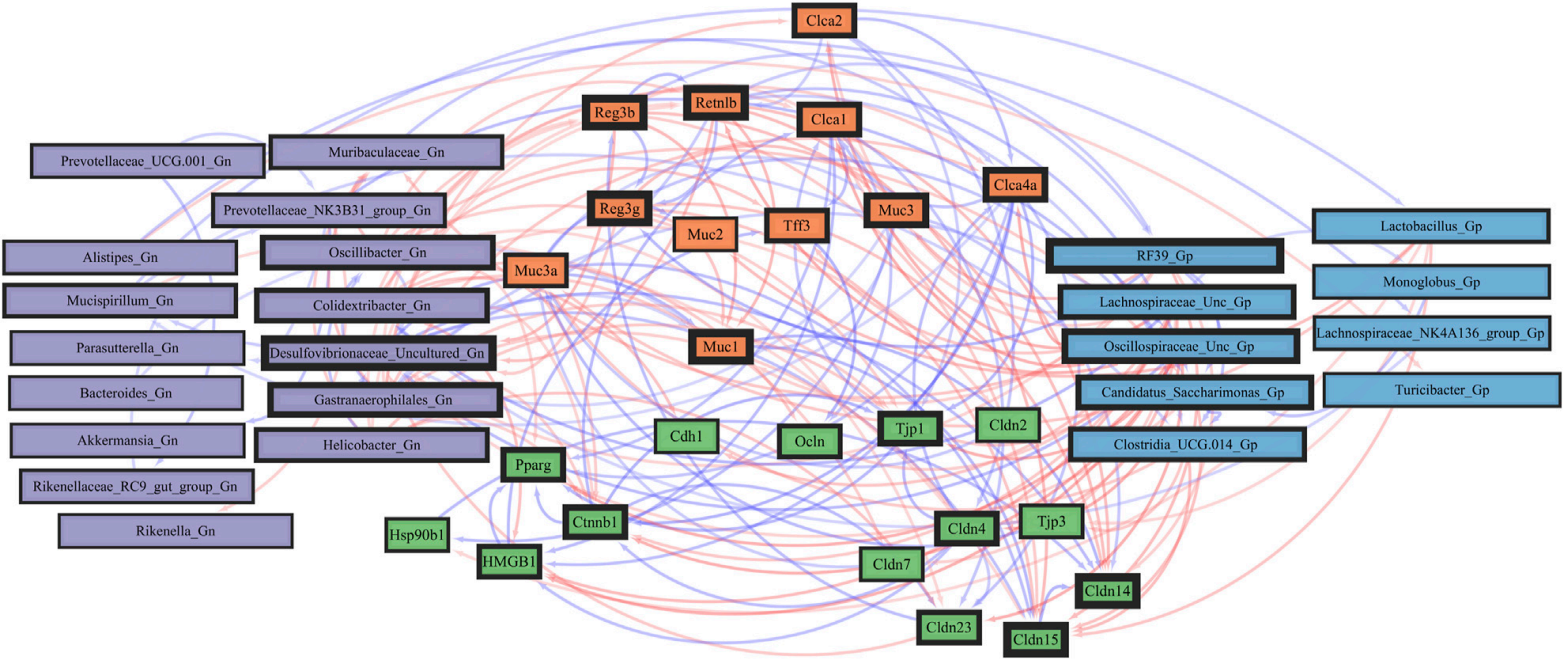
Network interactions depicting significant aging barrier genes and Gram-positive/Gram-negative gut microbes comparing 2-mth- and 25-month-old mice. Significant correlations (R < −0.5, R > 0.5; q < 0.05) generated from SparCC were exported and visualized as network plots within the open-source platform Cytoscape 3.10.0. Blue edges indicate a positive correlation, and red edges indicate a negative correlation. Purple nodes indicate Gram-negative genera, and blue nodes indicate Gram-positive genera. The colors for the genes and gene clusters (based on the Markov cluster algorithm) were defined using interaction networks in the STRING database v12.0. The thickness of the nodes or individual features was calculated using the cytoHubba module within Cytoscape 3.10.0 and ranked by importance (thick black square: higher importance; thin black square: lower importance) using the maximal clique centrality method.

Next, we feature the correlation analysis between the core 26 barrier, mucus, shaping, and junction genes and 28 combined bacterial Gp and Gn genera that were examined for the age span comparison between 2-mth- and 25-mth-old mice (Figure 7; Supplementary Data Sheet S8). The relationships between these barrier function/integrity genes (MCC network rankings: *Reg3γ*, *Reg3ß*, RELMβ (*Retnlb*), *Clca4a*, *Tjp1*, *Ctnnb1*, *Cldn4*, *Clca1*, *Pparg*, *Clca2*, *Cldn23*, and *HMGB1*) were positively associated with downregulated expression with one another over time and negatively associated with higher abundances of dysbiotic Gn genera (MCC Rankings: *Desulfovibrionaceae* Unclassified, *Gastranaerophilales*, *Oscillibacter*, *Colidextribacter*, and *Helicobacter*) with aging (Figure 8; Supplementary Data Sheets S8, 12). Interestingly, the significant downregulation of barrier function/integrity genes *Reg3γ*, *Reg3ß*, RELMβ (*Retnlb*), and *Tjp1* indicated negative associations with the upregulation of specific mucin, tight junction, and heat-shock proteins (MCC rankings: *Muc1*, *Cldn14*, *Cldn15*, *Muc3*, *Muc3a*, and *Hsp90b1*) in all three mice time span comparisons. These mucin, tight junction, and heat-shock proteins being overexpressed with aging could be the result of potentially overcompensating for the significant loss of core intestinal barrier function/integrity proteins identified.

In the early phase of aging between 2-mth- and 15-mth-old mice, the majority of these core barrier function genes (MCC network rankings: *Clca4a*, *Ctnnb1*, *Tjp1*, *Reg3ß*, RELMβ (*Retnlb*), *Reg3γ*, *HMGB1*, and *Clca1*) were positively associated with downregulated expression with each other, while negatively correlated with higher abundances of dysbiotic Gn genera (MCC rankings: *Oscillibacter* and *Muribaculum*) in aging (Supplementary Figures S9, S10; Supplementary Data Sheets S8, 12). The heatmap (Supplementary Figure 11; Supplementary Data Sheets S8, 12) depicts the significant associations between 15-mth- and 25-mth-old mice’s barrier function genes and genera.

Importantly, we found that the putative pro-inflammatory Gn bacteria that were significantly associated with the loss of core barrier function/integrity genes had great similarity to the significant associations found with the loss of core AMP gene expressions (Figures 5B; Figure 6B). These correlation outcomes further support the disrupted intestinal permeability (i.e., leaky gut) of immunofluorescence staining for ZO-1 with aging (Supplementary Table S7; Supplementary Figure S4). The significant gene pathway enrichment categories associated with the loss of core intestinal barrier function/integrity genes between 2-mth- and 25-mth-old mice are shown in Table 3 to highlight the potential effects of aging on human health. For significant gene pathway enrichment categories between 2-mth- and 15-mth-old mice, please refer to Supplementary Data Sheet S12.

### Integrative analysis of gut microbes and senescence genes

**FIGURE 9:**
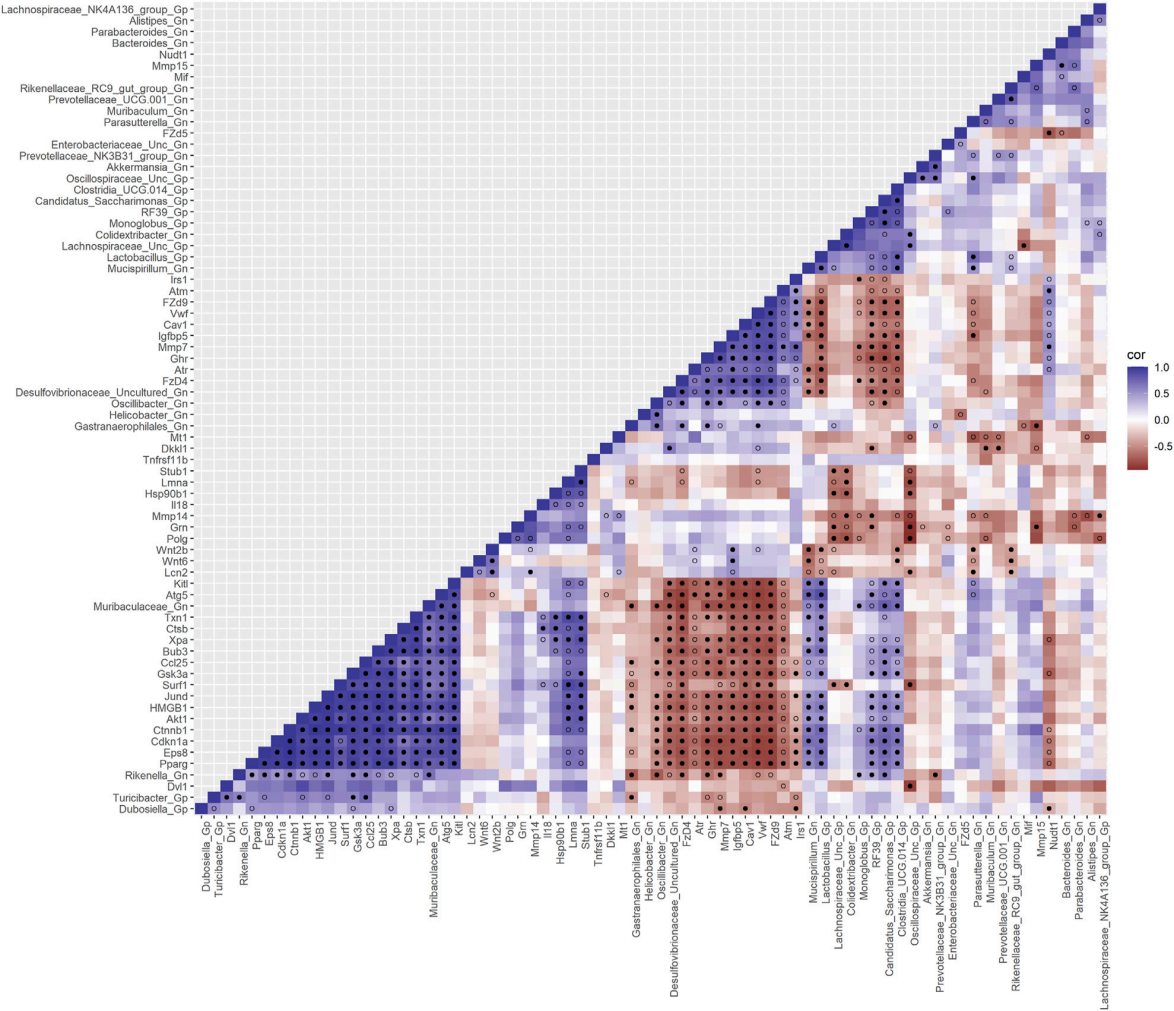
Interactions between aging senescence genes and Gram-positive/Gram-negative gut microbes comparing 2-mth- and 25-mth-old mice. SparCC plot depicting gene–microbe correlations. The colors indicate the magnitude of the correlation. *Pseudo p*-values were computed using 100 randomized sets and then corrected using the Benjamini–Hochberg method. The heatmap indicates significant associations with *p*-values (*p* < 0.05; empty circle) and q-values (q < 0.05; black circle). Gp, Gram-positive genus; Gn, Gram-negative genus.

**FIGURE 10:**
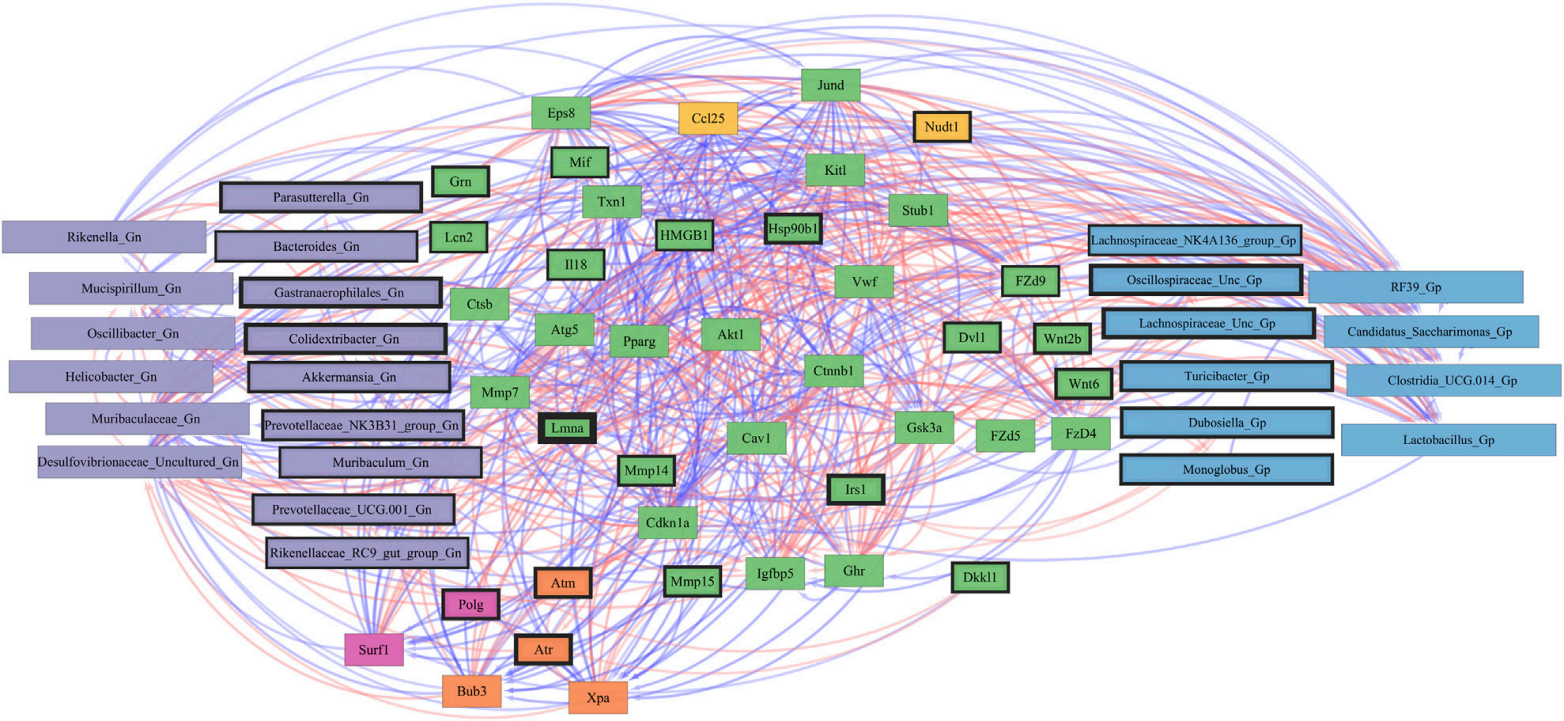
Network interactions depicting significant aging senescence genes and Gram-positive/Gram-negative gut microbes comparing 2-mth- and 25-month-old mice. Significant correlations (R < −0.5, R > 0.5; q < 0.05) generated from SparCC were exported and visualized as network plots within the open-source platform Cytoscape 3.10.0. Blue edges indicate a positive correlation, and red edges indicate a negative correlation. Purple nodes indicate Gram-negative genera, and blue nodes indicate Gram-positive genera. The colors for the genes and gene clusters (based on the Markov cluster algorithm) were defined using interaction networks in the STRING database v12.0. The thickness of the nodes or individual features was calculated using the cytoHubba module within Cytoscape 3.10.0 and ranked by importance (thick black square: higher importance, thin black square: lower importance) using the maximal clique centrality method.

Furthermore, to examine the relationships between cellular senescence genes and microbes in the colonic crypt epithelial cells and their roles in the consequences of aging, a correlation analysis was performed between 44 senescence genes and 28 bacterial Gp and Gn genera (Figure 9; Supplementary Data Sheet S9). Collectively, the positively associated downregulated expressions between *HMGB1*, *Ctnnb1*, *Eps8*, *Pparg*, *Akt1*, *and Cdkn1a* genes were additionally found to be negatively associated with the upregulation of cellular-inflammaging genes (MCC rankings: *Fzd9*, *Vwf*, *FzD4*, *Cav1*, *Igfbp5*, *Mmp7*, *Ghr*, *Irs1*, and *Atm*), as well as negatively associated with higher abundances of dysbiotic Gn genera (MCC rankings: *Desulfovibrionaceae* Unclassified, *Oscillibacter*, and *Gastranaerophilales*) (Figure 10; Supplementary Data Sheets S9, 13). Furthermore, the gene expression losses of *HMGB1*, *Ctnnb1*, *Eps8*, *Pparg*, *Akt1*, *and Cdkn1a* were positively associated with decreased abundances of Gp genera (MCC rankings: *Lactobacillus*, *Candidatus Saccharimonas*, *Clostridia UCG 014*, and *RF39*). Similarly, the upregulation expression of inflammaging genes like *Wtn2b*, *Cav1*, and *Fzd9* was negatively associated with lower abundances of Gp genera (MCC rankings: *Lactobacillus*, *Candidatus Saccharimonas*, *Clostridia UCG 014*, *RF39*, *and Dubosiella*) (Figure 10; Supplementary Data Sheets S9, 13). For additional correlation analyses on early and middle age associations between senescence genes and microbes, please see Supplementary Material (Supplementary Figures S12-14, Supplementary Data Sheets S9, 13).

Collectively, these senescence-associated gene and microbe relationships throughout the process of aging suggests an accumulation of cellular inflammaging gene expressions plus pro-inflammatory abundances of dysbiotic genera (Figure 6C), paired with decreased abundances of beneficial Gp genera, as previously described (Figures 1C–E). These data further support the increased expression of γH2AX in 25-mth-old mice when compared to 2-mth-old mice helping validate that the effects of aging could lead to an increase in the inflammatory response of DNA damage signaling in the colon (Supplementary Table S8; Supplementary Figure S5). The significant gene pathway enrichment categories associated with the increase in cellular inflammaging genes between 2-mth- and 25-mth-old mice are shown (Table 3) to highlight the potential effects of aging on human health. For significant gene pathway enrichment categories between 2-mth- and 15-mth-old mice, please refer to Supplementary Data Sheet S13.

### Integrative analysis of gut microbes and inflammation genes

**FIGURE 11:**
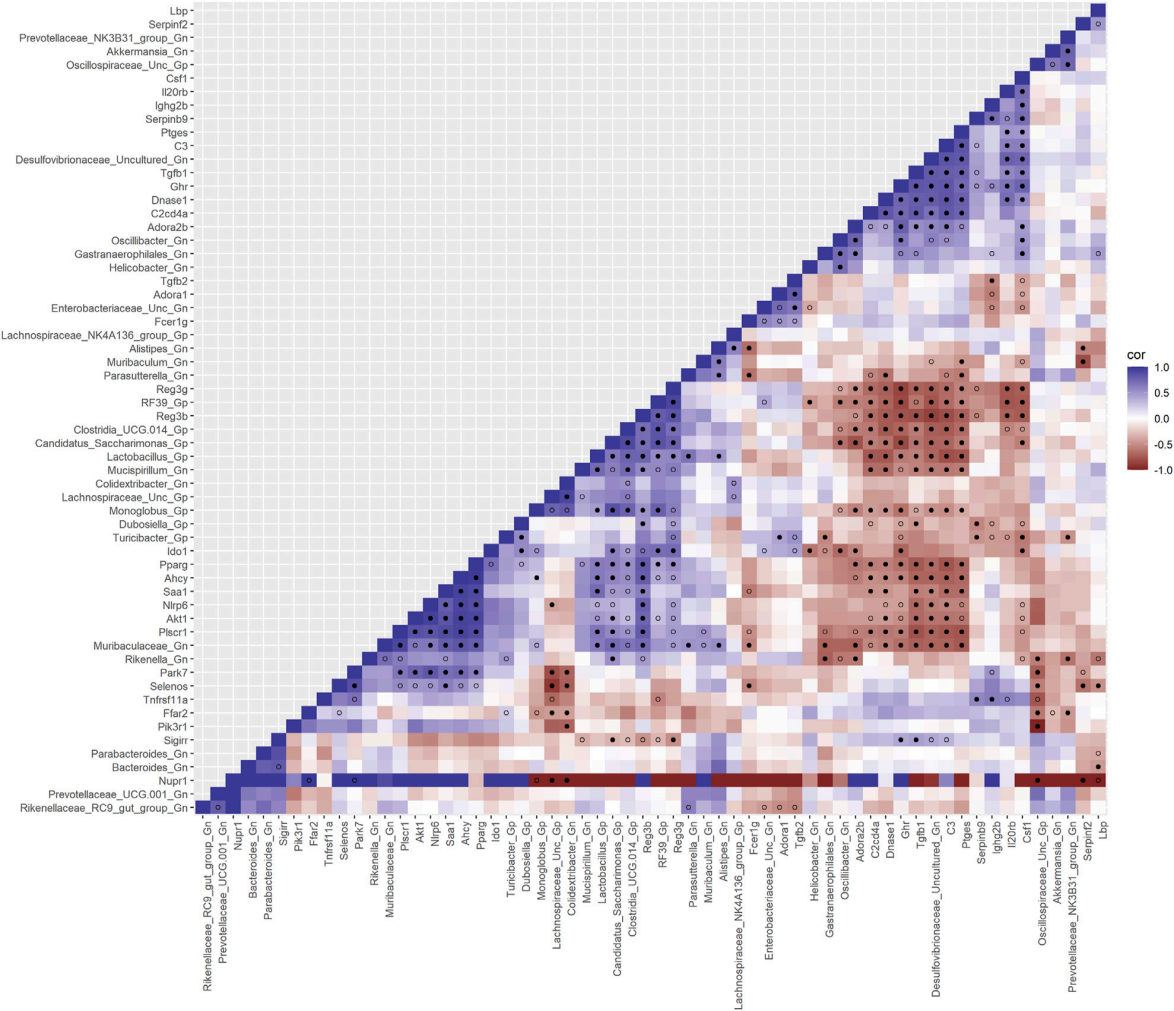
Interactions between aging inflammation genes and Gram-positive/Gram-negative gut microbes comparing 2-mth- and 25-mth-old mice. SparCC plot depicting gene–microbe correlations. The colors indicate the magnitude of the correlation. *Pseudo p*-values were computed using 100 randomized sets and then corrected using the Benjamini–Hochberg method. The heatmap indicates significant associations with *p*-values (*p* < 0.05; empty circle) and q-values (q < 0.05; black circle). Gp, Gram-positive genus; Gn, Gram-negative genus.

**FIGURE 12:**
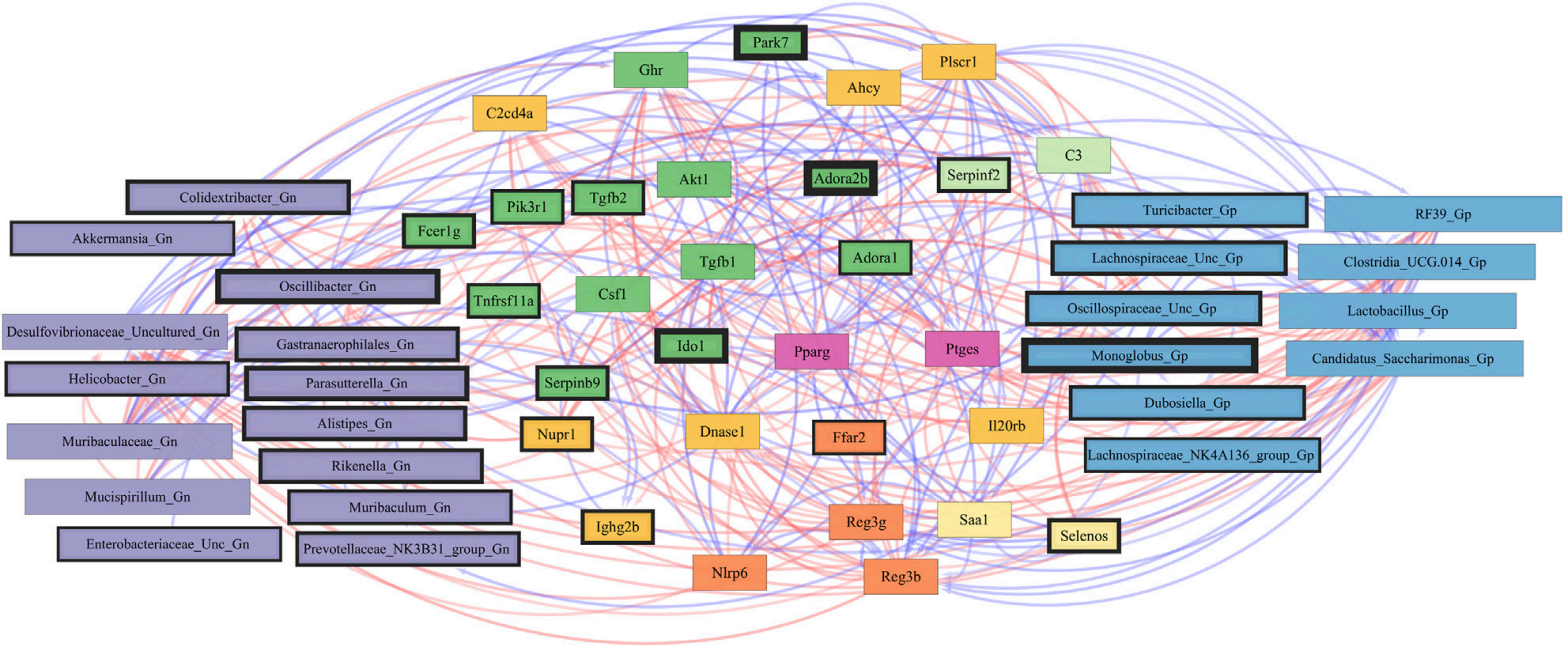
Network interactions depicting significant aging inflammation genes and Gram-positive/Gram-negative gut microbes comparing 2-mth- and 25-month-old mice. Significant correlations (R < −0.5, R > 0.5; q < 0.05) generated from SparCC were exported and visualized as network plots within the open-source platform Cytoscape 3.10.0. Blue edges indicate a positive correlation, and red edges indicate a negative correlation. Purple nodes indicate Gram-negative genera, and blue nodes indicate Gram-positive genera. The colors for the genes and gene clusters (based on the Markov cluster algorithm) were defined using interaction networks in the STRING database v12.0. The thickness of the nodes or individual features was calculated using the cytoHubba module within Cytoscape 3.10.0 and ranked by importance (thick black square: higher importance; thin black square: lower importance) using the maximal clique centrality method.

Finally, we explored the relationships between inflammation-associated genes and microbes in the colon for their potential roles in the process of aging. A correlation analysis between 32 inflammation genes and 28 bacterial Gp and Gn genera was examined between all three mice aging group comparisons (Figure 11; Supplementary Data Sheet S10). Collectively, the positively associated downregulated expressions of select intestinal homeostasis-, barrier-, and the microbiome-mediating genes (MCC network rankings: *Reg3ß*, *Pparg*, *Reg3γ*, *Nlrp6*, and *Akt1*) were additionally found to be negatively associated with the upregulation of inflammation-related genes (MCC rankings: *C3*, *Ptges*, *Dnase1*, *Ghr*, *Tgfb1*, *Csf1*, *and Il20rb*), as well as negatively associated with higher abundances of dysbiotic Gn genera (MCC rankings: *Desulfovibrionaceae* Unclassified, *Oscillibacter*, and *Parasutterella*). Furthermore, the upregulated expressions of inflammatory genes (MCC rankings: *C3*, *Ptges*, *Dnase1*, *Ghr*, *Tgfb1*, *Csf1*, *and Il20rb*) were negatively associated with decreased abundances of beneficial putative Gp genera (MCC rankings: *Lactobacillus*, *Candidatus Saccharimonas*, *Clostridia UCG 014*, *RF39*, and *Monoglobus*) (Figure 12; Supplementary Data Sheets S10, 14). For additional correlation analyses on early and middle age associations between inflammation genes and microbes, please see Supplementary Material (Supplementary Figures S15–17; Supplementary Data Sheets S10, 14).

These inflammation-associated gene and microbe relationships further support prior studies suggesting that aging is characterized by chronic inflammation plus pro-inflammatory taxa (Figure 6D), which promotes senescence and loss of function over time and is another facet of “inflammaging.” The significant gene pathway enrichment categories associated with the increase in inflammation-associated genes between 2-mth- and 25-mth-old mice are shown (Table 3) to highlight the potential effects of aging on human health. For significant gene pathway enrichment categories between 2-mth- and 15-mth-old mice, please refer to Supplementary Data Sheet S14.

## Discussion

The goals of this study were to (LeBrasseur et al., 2015) characterize the mouse colonic microbiota community associated with aging (López-Otín et al., 2023), determine whether AMPs, intestinal barrier, and inflammaging gene expressions are impacted by aging, and (Campisi et al., 2019) investigate how aging-related changes in colonic crypt epithelial cell gene expression might correlate with the aging colonic fecal microbiome in the widely validated aging C57BL/6 mouse model. We analyzed these data for three mouse age spans: 2–15 mth, 15–25 mth, and 2–25 mth. Colonic crypt epithelial cells were extracted and processed for RNA-seq analysis, with the resulting data for each of the three time points identifying more than 14,000 DEGs for each time point comparison (2–15 mth: 14,777 DEGs; 15–25 mth: 16,521 DEGs; and 2–25 mth: 15,692 DEGs) (Figures 2C–E; Supplementary Data Sheets S4–S6). We then compared differences between the three time points, resulting in three sets of differential gene expression RNA-seq and 16S rRNA microbiota data matched for each time span.

Overall, our study has several important conclusions: 1) our study is consistent with prior published aging intestinal microbiota studies demonstrating the significant aging-related hallmark increased relative abundances of Gram-negative “dysbiotic” bacteria in the colon (stool) in our 15-mth- and 25-mth-old mice. 2) We showed what we feel is one of the most remarkable examples of aging colonic crypt epithelial cell RNA-seq gene expressions published to date, with more than 14,000 DEGs at each of the three aging time spans: 2–15 mth (human: 18 yo), 15–25 mth (human: 50 yo), and 2–25 mth (human: 84 yo). 3) Our colonic crypt epithelial cell RNA-seq was performed on mice, for which we had corresponding 16S rRNA fecal microbiota data for the three mouse aging time points. 4) After preliminary analysis of our RNA-seq data, we noted dramatically lower gene expression of AMPs *RELMβ* (*Retnlb*) with 80% loss at 25 mth (q = 7.37369^E-05^) and several other core AMP genes (Figure 3A). Thus, we chose to focus our analysis on five specific colonic AMP DEGs that were significantly downregulated with aging: *RELMβ*, *Reg3β*, *Reg3γ*, *Ang4*, and *Lypd8l*. Our data revealed that significant downregulation (q < 0.05) of these core AMP gene expressions in the colonic crypt epithelial cells with aging strongly negatively correlated (R < −0.5 and q < 0.05) with signature increased relative abundances of Gn dysbiotic genera (Figures 4, 5A, 6A). 5) The gene expression losses of these core five AMPs with aging, especially *RELMβ*, strongly positively correlated (R > 0.5 and q < 0.05) with the expression losses of key intestinal barrier homeostasis genes, including *Ctnnb1*, *Clca1*, *Clca2*, *Clca4a*, *Hmgb1*, *Tjp1*, and *Cldn4* (Figure 7). 6) Finally, and remarkably, we found the significant Gn dysbiotic genera, which significantly correlated with changes in gene expressions in our three curated gene sets of barrier, senescence, and inflammation were each a subset of the Gn genera that significantly correlated with the expression loss of our AMP genes (Figures 6B–D). Taken together, these data support a vital role for the aging-related loss of colonic AMP gene expression in Gn dysbiosis and inflammaging.

Because of the large numbers of DEGs per aging mouse comparison, we sought to focus our analysis on evaluating our hypothesis. We designed four gene sets to analyze in detail (Wang et al., 2021). Group 1 consists of genes coding for known colonic AMPs and related potential anti-microbial peptides, such as *S100g* and *S100a10* (Blyth et al., 2020; Singh and Ali, 2022; Song et al., 2023) (Figure 3A; Supplementary Table S2). As noted above, expression loss of these AMP genes with aging became our secondary hypothesis in this study after we determined that RNA-seq analysis revealed significant (q < 0.05) downregulation of several key AMPs. This downregulation was especially notable for the five AMPs that we will focus on: *RELMβ*, *Reg3β*, *Reg3γ*, *Ang4*, and *Lypd8l*. Interestingly, *Reg3β*, *Reg3γ*, *Ang4*, and *Lypd8l* were strongly positively correlated (R > 0.5, q < 0.05) with *RELMβ* expression loss with aging. These gene expression losses were also strongly negatively correlated (R < −0.5 and q < 0.05) with increased Gn bacteria with aging. Several studies in mice using knockout technology, including *RELMβ* and *Reg3β/γ* AMPs KO mice or recombinant AMPs given rectally, support our data for increased abundances of these core AMPs in Gn bacteria with gene expression loss in our study (discussed below). We found no cathelicidin AMPs in the colon expressed in our DEGs and only two beta-defensin AMPs at low levels: defensin β37 and defensin β45, both upregulated compared to other AMPs (Figure 3A). Significantly, our data agree with one other recent BL/6 aging mouse study that looked at colonic gene expression using colonic epithelium scrapings and compared 12 mth to 28 mth gene expression using microarray analysis. They found 3 of the 15 genes exhibiting the greatest colonic loss at 28 mth (in 1,371 DEGs) were *RELMβ*, *Reg3β*, and *Ang4* and noted this pointed toward a dysregulation of anti-microbial peptide expressions at old age (van der Lugt et al., 2018).

We then designed a second curated gene set to evaluate gene expressions related to barrier function, such as ZO-1 (*TJP1*). ZO-1 is a tight junction protein critical to maintaining the gut barrier. We designed a third gene set (senescence) and a fourth gene set (inflammation) to investigate potential inflammaging-related mechanisms for the observed increase in Gn bacteria in the fecal microbiota data. Furthermore, we aimed to investigate whether these aging-related genes could provide a mechanistic insight into the loss of our five key AMP genes with age or if they were a consequence of AMP expression loss.

Importantly, rather than simply mapping global gene expression, we focused our analysis on evaluating a specific hypothesis: *we sought to determine whether aging-related loss of colonic epithelial crypt cell AMPs gene expression might correlate with the increase in pro-inflammatory bacteria in the colon (stool) microbiota, especially Gn pro-inflammatory bacteria*. Specifically, we also sought to examine aging-associated changes in colonic crypt epithelial cell gene expression that might explain the increased relative abundances of Gn bacteria (“dysbiosis”) of aging, as well as increased gut leakiness and inflammaging (Ghosh et al., 2022; Hohman and Osborne, 2022).

AMPs are short cationic peptides produced by a variety of cells in the body (in this case the colon goblet and epithelial cells) that repress/modulate or kill microorganisms and are part of our innate immune defense (Blyth et al., 2020; Song et al., 2023). However, other key functions of AMPs, which we propose may be significant, are potent colon homeostasis functions, including anti-inflammatory properties, recruitment of immune cells, wound healing, and cytokine production, and our data support the idea that these functions may also be lost with the loss of AMPs (Chen et al., 2019; Blyth et al., 2020; Shi et al., 2023). Some AMP studies, for example, in inflammatory bowel disease (IBD), claim that the anti-inflammatory properties of AMPs are more important for colonic homeostasis than their anti-microbial activity (Shindo et al., 2020). Intestinal colonic goblet cells and enterocytes produce several types of AMPs (Blyth et al., 2020; Song et al., 2023). Importantly, although ∼90% of the microbiome resides in the colon, most of the research interest over the years has focused on small intestinal AMPs, especially ileal Paneth cell cathelicidins, defensins, and *Reg3ß/γ* AMPs (Mukherjee and Hooper, 2015). We believe our study may be the first in-depth analysis to investigate the effect of aging on colonic AMPs in mice.

Our data strongly support a role for the loss of these five key AMPs (and other gene expression changes) with aging, significantly correlating with the classic increase in aging Gn dysbiosis bacteria, as well as compelling evidence of loss of barrier gene function and increased inflammaging gene markers in our aging mouse model. We propose that our data also supports a significant role for the aging loss of *RELMβ* gene expression in this model. A recent review characterizes *RELMβ* as a “critical regulator of colon homeostasis and intestinal permeability” and a regulator of AMPs in the colon, especially the *Reg3β* and *Reg3γ* AMPs (Hogan et al., 2006; Blyth et al., 2020). Our data support this role for colonic *RELMβ* and showed that the expression loss of *RELMβ* strongly positively correlated (R > 0.5 and q < 0.05) with expression losses of *Reg3β*, *Reg3γ*, *Ang4*, and *Lypd8l* AMPs (Figure 4). Our RNA-seq expression data show that in the full study (2 mth–25 mth comparison), *RELMβ* expression loss is ∼80%, which strongly significantly correlated with the expression losses of each key AMP: *Reg3β* (86%), *Reg3γ* (74%), *Ang4* (94%), and *Lypd8l* (68%). The data for gene expression losses of both *Reg3β* and *Reg3γ* are consistent with studies in *RELMβ* KO mice in which *Reg3β/γ* expressions were lost in the colon and correlated with increased gut leakiness (Hogan et al., 2006; Blyth et al., 2020). However, we believe our data are one of the first to show this effect of *RELMβ* expression loss in the mouse colon with aging, as well as associated colonic expression losses of the AMPs *Reg3β*, *Reg3γ*, *Ang4*, and *Lypd8l* with aging strongly correlating with the expression loss of *RELMβ*.

Importantly, the AMPs such as *RELMβ*, *Reg3β*, *Reg3γ, Ang4*, and *Lypd8l* also exhibit anti-inflammatory properties (Shi et al., 2023). For example, rectal delivery of these peptides corrects colonic dysbiosis and blocks inflammation in mouse colitis models (Darnaud et al., 2018; Blyth et al., 2020). However, the cellular receptors for these five AMPs have not yet been identified in humans or mice (Chen et al., 2019; Shi et al., 2023). Colonic *Lypd8l* AMP is newly discovered, and cell sources are not yet well defined (until our data), although related *Lypd8* AMP is better characterized and also expressed by colon goblet cells in our data and slightly down at 25 mth. Paneth cell AMPs observed in our colon expression data include *Reg3β*, *Reg3γ*, and *Ang4* in our list, but *RELMβ* is expressed virtually only in the colon, with only a trace amount found in the ileum (Steppan et al., 2001; Hogan et al., 2006).

The first four of these five AMPs have been characterized as exhibiting specific anti-microbial activity as well as other beneficial regulation of the intestinal barrier and inflammation. Our microbiota data further support the aging models previously shown in other studies of mice and humans for significant increased abundances of pro-inflammatory/dysbiotic Gn bacteria and loss of beneficial Gp bacteria with aging (Figures 1C–E). (van Tongeren et al., 2005; Claesson et al., 2012; Langille et al., 2014; Biagi et al., 2016; van der Lugt et al., 2018; Wang et al., 2019; Wu et al., 2021). Our data also support that gene expression losses of the five AMPs, as described above for *RELMβ*, *Reg3β*, *Reg3γ*, *Ang4*, and *Lypd8l*, all strongly negatively correlate with increased abundances in Gn genera (Figures 4; Figure 5A). The *RELMβ* (gene symbol *Retnlb*) is a cysteine-rich peptide that forms multimers and punches holes in the cell membranes of Gn bacteria. Mice have four RELM proteins (RELMα, -β, -γ, and resistin) encoded by Retlna (not found, NF), Retlnb, Retlng (NF), and Retn (NF) genes, respectively, whereas humans possess only *RELMβ* and resistin encoded by *RETLNβ* and *RETN* genes (Steppan et al., 2001; Artis et al., 2004) We only found *RELMβ* (*Retnlb*) expressed in our mouse colons (Propheter et al., 2017; Blyth et al., 2020). All mouse and human *RELMβ* proteins are detectable in the serum and stool, offering the potential to utilize *RELMβ* levels as biomarkers (He et al., 2003). *RELMβ* is predominantly and constitutively expressed in the colon by goblet cells and enterocytes but virtually absent in the small intestine (Steppan et al., 2001; He et al., 2003). *RELMβ* regulates innate immune (anti-Gn bacteria and worms) colonic function as well as colonic homeostasis, including barrier permeability and inflammation susceptibility (Hogan et al., 2006; Blyth et al., 2020; Shi et al., 2023). *RELMβ* also regulates systemic insulin resistance (Pine et al., 2018; Shi et al., 2023). Importantly, gene array RNA expression analysis of *RELMβ* KO mice colon tissue revealed a nearly total loss of *Reg3β* and *Reg3γ* expression (Hogan et al., 2006) consistent with our study data for significant positive correlations for *Reg3β* and *Reg3γ* expression losses with *RELMβ* expression loss with aging in our mouse colons (Figure 4). Importantly, the array analysis also revealed that over 50 other colonic genes were affected by *RELMβ* KO in those mice, supporting a key role for *RELMβ* in colonic permeability and homeostasis, as we also propose (Hogan et al., 2006; Pine et al., 2018; Shi et al., 2023). The microbiome data observed in *RELMβ* KO mice in another study are also significant (Propheter et al., 2017). The Hooper lab showed that *RELMβ* is an AMP that kills Gn bacteria *in vitro* and promotes critical spatial segregation of Gn bacteria from the epithelium in mouse colons. *RELMβ* limits the entry of Gn bacteria into the colon’s inner mucus layer (Propheter et al., 2017). The *RELMβ* KO resulted in a dramatically increased abundance of Gn phylum Proteobacteria and increased association and invasion of those bacteria with the colonic epithelium (not Gp bacteria). Particularly relevant to our microbiota data, that study noted a specific increase in colon Gn Proteobacteria-associated genus *Helicobacter* in *RELMβ* KO mice, virtually absent in their control mice (as with our 2 mth-old mice microbiota data) (Propheter et al., 2017). In our fecal microbiota data, the relative abundance of the genus *Helicobacter* was significantly increased in the 25 mth-aged mice and negatively correlated (R < −0.5, q < 0.05) with the 80% loss of *RELMβ* expression (Figure 4). Thus, colonic gene expression and microbiota data from *RELMβ* KO mice support the *Reg3β/γ* expression loss and microbiota alterations shown in our 25-mth-old aging mice with 80% colonic *RELMβ* loss. Additional pro-inflammatory Gn genera relative abundances that increased with *RELMβ* expression loss in our 25-mth-old mice included *Gastranaerophilales*, *Oscillibacter*, *Desulfovibrionaceae* Uncultured, and *Prevotellaceae* UCG-001 (Figures 4; Figure 5A; Figure 6A).

*Reg3β* and *Reg3γ* proteins are also key intestinal AMPs expressed in both ileum Paneth cells and colonic enterocytes and goblet cells (Blyth et al., 2020; Sun et al., 2021). Most microbiome data on these AMPs come from ileal Paneth cell studies (Mukherjee and Hooper, 2015). Some studies claim that *Reg3β* is not expressed by colonic enterocytes, but our gene expression data show significant downregulated expressions of both *Reg3β* and *Reg3γ* in our colonic crypt epithelial cells of 25-mth-old mice (Figure 3A). Studies in mice show *RELMβ*, as well as *Reg3β* and *Reg3γ*, require a microbiome to be expressed in the intestine (Hogan et al., 2006; Blyth et al., 2020). As noted above, *RELMβ* KO mice expressed almost no colonic *Reg3β* or *Reg3γ* (Hogan et al., 2006). Our 2 mth–25 mth gene expression data showed loss of *RELMβ* (80%) strongly positively correlated (R > 0.5 and q < 0.05) with downregulated losses of *Reg3β* (86%) and *Reg3γ* (74%). Our data support this at all three aging time points, indicating that gene expression loss of RELMβ strongly positively correlates (R > 0.5 and q < 0.05) with gene expression losses of *Reg3β* and Reg3γ. Both *Reg3β* and *Reg3γ* AMPs contain a C-type lectin domain that binds bacteria and punches holes in the cell (Blyth et al., 2020). *Reg3β* has bactericidal activity against Gn bacteria and protects mice against intestinal infection and dissemination of Gn bacteria, including the species *Salmonella enteritidis* (Miki et al., 2012; van Ampting et al., 2012). *Reg3β* KO mice have significantly greater Gn bacteria in their intestines, as shown in our data (Wang et al., 2016). *Reg3γ* has bactericidal activity against Gp bacteria, helps maintain the spatial segregation of luminal bacteria and the intestinal epithelial surface, and prevents invasion (Cash et al., 2006; Vaishnava et al., 2011; Mukherjee et al., 2014). Mice with loss of *Reg3γ* protein expression with gene KO or high-fat diet (which represses *Reg3γ* expression) exhibit disrupted diurnal rhythms of mucosa-associated microbial abundance within the colon and disrupted systemic metabolism (Thaiss et al., 2016; Frazier et al., 2022). Mice overexpressing *Reg3γ* have lower intestinal bacterial loads and bacterial translocation after ethanol feeding (Wang et al., 2016). These findings support a model in which *Reg3γ* reduces the number of bacteria on mucosal surfaces of the intestine to limit bacterial translocation and participates in regulating the microbiome circadian rhythm and systemic metabolism (Wang et al., 2016; Choi et al., 2021). The human homolog of mouse *Reg3γ*, called REG3A, has shown remarkable properties. When delivered rectally in mice, it reduces colitis inflammation and dysbiosis (Darnaud et al., 2018). As discussed further below, the anti-inflammatory properties of *RELMβ* and *Reg3β/γ* AMPs may be more important than their direct anti-microbial properties in maintaining colonic homeostasis (Shindo et al., 2020). These AMPs have even been called “gut hormones” (Shin and Seeley, 2019; Shi et al., 2023).

In our 25-mth-old mice, the downregulation of gene *Reg3β* (86%, q = 0.005) was strongly positively correlated (R > 0.5, q < 0.05) with gene expression losses of all four core AMPs we focused on: *RELMβ*, *Reg3γ*, *Ang4*, and *Lypd8l* (Figure 4). As noted above, *Reg3β* was identified as specifically toxic to Gn bacteria but not Gp bacteria (Miki et al., 2012). Our 25-mth data showed that gene expression loss of *Reg3β* was also negatively correlated with increased relative abundances of Gn pro-inflammatory genera: *Oscillibacter, Helicobacter, Gastranaerophilales,* *Desulfovibrionaceae* Uncultured, and *Alistipes* (Figure 4). In addition, *Reg3β* gene expression loss was positively correlated with decreased relative abundances of beneficial Gp genera *RF39*, *Clostridia* UCG-014*, and Lactobacillus* (Figure 4). Furthermore, in our 25-mth-old mice, gene expression loss of *Reg3γ* (74%, q = 0.032) strongly positively correlated (R > 0.5, q < 0.05) with downregulated gene losses of *RELMβ*, *Reg3β*, and *Lypd8l* but surprisingly not *Ang4* (Figure 4). It is also interesting that the gene expression loss of *Reg3γ* is negatively correlated with increased relative abundances of Gn genera *Helicobacter*, *Parabacteroides*, *Alistipes*, *Prevotellaceae* UCG-001, *Oscillibacter*, *Desulfovibrionaceae* Uncultured, and *Gastranaerophilales*. *Reg3γ* gene expression loss was positively correlated with decreased relative abundances of beneficial Gp genera *RF39*, *Candidatus Saccharimonas*, *Clostridia* UCG-014, and *Lactobacillus* (Figure 4).

Both humans and mice express angiogenin family proteins (each ∼120 aa peptides). Angiogenin-4 (*Ang4*) was originally identified in ileum Paneth cells (Hooper et al., 2003) but, more recently, in colonic goblet cells (Forman et al., 2012). Ang4 expression is also induced by the microbiome, similar to *RELMβ* and *Reg3β/γ* proteins (Hooper et al., 2003). *Ang4* exhibits RNase activity, as do most family members, but *Ang4* anti-microbial activity was recently shown to not be dependent on its RNase properties (Sultana et al., 2022a; Sultana et al., 2022b). *Ang4* also exhibits angiogenesis properties (Sultana et al., 2022b). A recent study in mice administered mouse *Ang4* protein (mAng4) rectally and then analyzed 16S rRNA colonic microbiota composition alterations days later (Sultana et al., 2022a). Broadly speaking, *Ang4* was described as promoting the growth of beneficial bacteria and killing pathobionts (Sultana et al., 2022a). *Ang4* treatment resulted in increased abundances of beneficial genera, including *Lactobacillus, Akkermansia*, *Dubosiella*, *Coriobacteriaceae* UCG-002, and *Adlercreutzia,* but also decreased certain pathogenic Gn bacteria, including *Alistipes* and *Enterohabdus.* Expression loss of *Ang4* (94%, q = 8.33883^E-08^) in our 25 mth mice strongly positively correlated (R > 0.5 and q < 0.05) with downregulation gene expression losses of *RELMβ*, *Reg3β*, and *Lypd8l* but not with *Reg3γ* (Figure 4). The 94% expression loss of *Ang4* was strongly positively correlated (R > 0.5 and q < 0.05) with a decreased relative abundance of Gp *Lactobacillus* (supported by the rectal study above), as well as increased relative abundances in Gn bacteria *Colidextribacter* and *Desulfovibrionaceae* Uncultured.

Very little is known about mouse AMP *Lypd8l*, but more is known about its close relative *Lypd8* (Okumura et al., 2016). We include *Lypd8l* because it exhibits a dramatic loss in our 25 mth mice (68%, q = 5.52438^E-06^) and is proposed to function like *Lypd8* and repress Gn flagellated bacteria by binding to flagella to block bacterial function (Okumura et al., 2016; Blyth et al., 2020). Perhaps most importantly, our correlation analysis showed that gene expression loss of *Lypd8l* at 25 mth strongly positively correlated (R > 0.5, q < 0.05) with downregulation gene expression losses of *RELMβ*, *Reg3β*, *Reg3γ*, and *Ang4* (Figure 4). It is also interesting that gene expression loss of *Lypd8l* is strongly negatively correlated (R < −0.5, q < 0.05) with increased relative abundances of Gn genera *Helicobacter, Alistipes*, Prevotellaceae UCG-001, *Oscillibacter,* Desulfovibrionaceae Uncultured, and *Gastranaerophilales*. The *Lypd8l* gene expression loss was positively correlated with decreased relative abundances of beneficial Gp genera *RF39*, *Candidatus Saccharimonas*, and *Lactobacillus* (Figure 4).

Our preliminary analysis revealed a significant gene expression loss of colonic AMPs with aging, potentially resulting in increased abundances of Gn dysbiotic bacteria, as observed in our 15-mth- and 25-mth-old mice, as well as in many microbiome aging studies. Based on this hypothesis, we further hypothesized that other key genes related to intestinal (colonic) epithelial cell inflammaging might correlate with the loss of AMPs (as a potential cause for AMPs expression loss or as a consequence of AMPs loss), as well as increased Gn bacterial gut dysbiosis (DeJong et al., 2020). There is no National Institute of Health (NIH)’s formal aging gene database. Therefore, we combined what databases were currently known online and incorporated our own curated gene sets (see *Methods*) broadly related to three additional categories: intestinal barrier function, senescence (the mechanism of aging), and inflammation, also called inflammaging, as a major driver of aging-related loss of cell function (Franceschi and Campisi, 2014; López-Otín et al., 2023). Because there are so much data for each curated gene set, we will include only the entire study 2 mth–25 mth outcomes/correlations in our discussion below and limit the number of key genes we can discuss.

Barrier-related genes. Increased intestinal permeability (leaky-gut and loss of barrier) is a key element driving systemic inflammation in aging humans, mice, and *Drosophila* (Clark et al., 2015; Buford, 2017; Scott et al., 2017; Ahmadi et al., 2020). Old mouse microbiome FMT transfer to young mice leads to increased translocation of inflammatory bacterial products into the circulation (Fransen et al., 2017; Thevaranjan et al., 2017). Important in this barrier gene group is β-catenin (*Ctnnb1*) expression loss. Our data revealed a 50% expression loss (q = 2.8599^E-11^) of *Ctnnb1* in our 25 mth colonic cells with aging (Figure 3B). β-catenin is the lynchpin of *Wnt* signaling in the intestinal epithelium, especially intestinal stem cells (ISCs) in our colonic crypt cells (Nalapareddy et al., 2022). *Wnt* signaling is the engine that drives intestinal epithelial differentiation/function, and the loss of *Ctnnb1* with aging is critical to the loss of intestinal epithelial cell (IEC) homeostasis (Asano et al., 2022). The restoration of *Ctnnb1* signaling alone rescues aging IEC from senescence and death (Nalapareddy et al., 2017; Nalapareddy et al., 2022). The expression loss of *Ctnnb1* was strongly positively correlated (R > 0.5, q < 0.05) with the expression losses of several key barrier proteins, including *Tjp1*, *Ppar*γ, *Cldn23*, and *Clca1* (Figure 7). The expression loss of β-catenin was also positively correlated with (R > 0.5 and q < 0.05) *RELMβ*, *Reg3β*, *Reg3γ*, and *Hmgb1* expression losses. ZO-1 (*TJP1*) is the key tight-junction protein that regulates intestinal permeability and has been shown to decrease with aging resulting in leaky-gut and increased systemic inflammation (Tran and Greenwood-Van Meerveld, 2013; Lustgarten, 2016). Our data showed a significant loss of ZO-1 expression of 25% in the colon of our aging mice (q = 0.0008) (Supplementary Table S7; Supplementary Figure S4) In our data, *Tjp1* expression loss is positively correlated (q < 0.05) with expression losses of both *PPARγ* and *Clca1* (a metalloprotease that promotes normal *Muc2* protective mucus configuration; loss of *Clca1* is associated with abnormal *Muc2* and loss of barrier protection (Nyström et al., 2018). *Pparγ* regulates the intestinal barrier in many models, and *Pparγ*-agonists such as pioglitazone improve the gut barrier (Zhao et al., 2018). Expression loss of *Hmgb1* (also associated with senescence-inflammaging) was correlated (*p* < 0.05) with expression losses of AMPs *RELMβ* and *Reg3β*. The AMPs *Ang4* and *Lypd8l* were not included in the barrier-curated gene set. There are many significant negative correlations between downregulated barrier genes and increased abundances of Gn genera. Importantly, we found that the Gn genera that were significantly increased in abundance in the barrier group had great similarity to the Gn genera in that their abundances increased with the loss of AMP gene expressions, which was also true for the senescence- and inflammation-curated gene sets (Figure 6).

Senescence genes. A key element in current models of aging is increased cellular senescence, which promotes an inflammatory milieu of cytokines and factors released from senescent cells (Baker et al., 2011; Wang et al., 2021) that in turn drives inflammation and aging, currently called “inflammaging” (Franceschi and Campisi, 2014; LeBrasseur et al., 2015; Wissler Gerdes et al., 2020; Dugan et al., 2023). The increased abundance of Gn bacteria and leaky gut are also key elements of this current inflammaging model (Penfield et al., 2013). We used gene markers from the *Database of Cell Senescence Genes* (see *Methods*) and our own literature search to define the next group of senescence-inflammaging genes to assess in our analysis. Once again, we saw *Ctnnb1* exhibiting the greatest loss in 25-mth-old mice (q = 2.8599^E-11^) (Figure 3C). Gene expression loss of this key element in the *Wnt* signaling pathway has been strongly associated with aging, as noted above (Cui et al., 2019). *Hmbg1* is a nuclear protein that, when lost, has been called the gateway cellular event to initiate cell senescence (Davalos et al., 2013). Significantly, our data showed that the expression loss of *Ctnnb1* was strongly correlated (R > 0.5 and q < 0.05) in our colonic cells with expression loss of *HMGB1* (Figure 9). Another key senescence-promoting marker that we identified was heat shock protein *Hsp901b* (aka Grp94), which was significantly elevated (319%, q = 0.003) in 25-mth-old mice. This *Hsp901b* has received much recent attention as a target to inhibit to prevent senescence in humans (Cui et al., 2022) and positively correlates (*p* < 0.05) with upregulated expression of *IL-18* (an *NLRP3* inflammasome cytokine) in our 25-mth-old colon cells. Finally, p21 (*Cdkn1a*) gene expression loss strongly positively correlated (R > 0.5, q < 0.05) with expression losses of multiple genes, including *Ctnnb1* and *Hmgb1*, as well as increased abundances in several Gn genera (Figure 6C). AMPs were not included in this senescence-curated gene set but will be in future studies.

Inflammation genes. Aging is characterized by chronic inflammation, which promotes senescence and loss of function over time, which is another facet of “inflammaging” (Franceschi et al., 2000; Chung et al., 2009; Franceschi and Campisi, 2014; Wang et al., 2021). Within this curated gene set, the greatest increased gene expression was complement component 3 (*C3*) in 25-mth-old mice (q = 3.88^E-06^) (Figure 3D). Upregulated *C3* expression has been strongly associated with decreased longevity (Fu et al., 2020; Zheng et al., 2022). The upregulation of C3 expression was strongly negatively correlated (R < −0.5 and q < 0.05) with downregulated expressions of AMPs *Reg3β* and *Reg3γ*, as well as *NLRP6* inflammasome loss, a key mediator of intestinal homeostasis and the microbiome (Elinav et al., 2018). Furthermore, the upregulation of *C3* expression strongly negatively correlated (R < −0.5 and q < 0.05) with the downregulated expressions of *Pparγ* (i.e., barrier control) and *Akt1*, a mediator of apoptosis. In addition, the downregulated expression of *Pparγ* with aging, as well as expression loss of *Akt1*, are positively associated (R > 0.5 and q < 0.05) with AMP *Reg3β* and *Reg3γ* expression losses.

Furthermore, we also found significant changes in several aging-related proteins (q < 0.05), including amyloid A expression loss (Figure 3D, q = 0.0001). Only AMPs Reg3β and *Reg3γ* were included in the inflammation curated gene set of analysis, and their expression loss in 25-mth-old mice was significantly negatively correlated (R < −0.5, q < 0.05) with upregulated expressions of several inflammation-related genes, like prostaglandin E synthase (*Ptges*), growth hormone receptor (*Ghr*), deoxyribonuclease I (*Dnase1*), interleukin 20 receptor beta (*Il20rb*), colony stimulating factor 1 (*Csf1*), and transforming growth factor beta 1 (*Tgfb1*) (Figure 11). Furthermore, the upregulated expressions of inflammatory genes *C3*, *Ptges*, *Dnase1*, *Ghr*, *Tgfb1*, *Csf1*, *and Il20rb* were negatively associated with decreased abundances of beneficial putative Gp genera *Lactobacillus*, *Candidatus Saccharimonas*, *Clostridia* UCG 014, *RF39*, and *Monoglobus* in 25 mth-old mice (Figure 1D; Figure 11).

As we noted above, in addition to dysbiosis, another crucial element in interpreting our data are the established anti-inflammatory properties of the AMPs we chose to focus on, especially the *RELMβ*, *Reg3β*, and *Reg3γ* AMPs that are the best studied. *Reg3β* and *Reg3γ* have been shown to exhibit potent anti-inflammatory effects in the ileum and colon and have even been suggested to be gut hormones (Chen et al., 2019; Shin and Seeley, 2019). Regenerating gene (Reg) family proteins serve as multifunctional secretory molecules with trophic, anti-apoptotic, anti-inflammatory, anti-microbial, and probably immuno-regulatory effects (Chen et al., 2019; Sun et al., 2021). DSS-induced colitis is aggravated in *Reg3β* KO mice relative to wild-type mice, suggesting that attenuation of colitis and ileitis is dependent on the function of *Reg3β* (Shindo et al., 2020) miRNA knockdown of *Reg3β* and *Reg3γ* promotes colitis in mice (Runtsch et al., 2015). *Reg3γ* KO mice have greater numbers of mucosal-attached bacteria, and *Reg3β* KO mice have greater numbers of stool Gn bacteria (Xiao et al., 2019). The rectal delivery of human hREG3A (homolog to mouse Reg3γ) in mice alters the intestinal microbiota and controls colitis inflammation in mice (Darnaud et al., 2018). hREG3A also resulted in a significant shift in colon microbiota, including an increased abundance of Gp-beneficial commensals, like Lachnospiraceae-associated short-chain fatty acid-producing taxa, and depleted abundances of Gn bacteria, like pro-inflammatory-producing Proteobacteria-associated taxa. Significantly, cohoused and germ-free mice fed feces from hREG3A transgenic mice developed less severe DSS colitis. This effect may be relevant to FMT studies, as we note below. Mice given hREG3A rectally developed less severe TNBS colitis (Darnaud et al., 2018). IL-33 has been shown to regulate *Reg3γ* expression, and IL-33 KO mice exhibit loss of *Reg3γ* and increased Gn colonic bacteria, including genus *Alistipes*, which we observed to increase in abundance in 25-mth-old mice (Xiao et al., 2019). Intestinal function, energy balance, circadian rhythms, and glucose regulation are disrupted in *Reg3γ* KO mice, while diets high in fermentable fiber increase circulating levels of *Reg3γ* in mice (Shin et al., 2022; Shin et al., 2023). *Reg3γ* has extensive ROS-scavenging ability and can also normalize glucose tolerance in mice (Le Lay et al., 2022). *Reg3β/γ* also promotes colonic epithelial cell proliferation and healing (Blyth et al., 2020).

Our study supports a potential central role for loss of colonic AMP *RELMβ* with aging-related dysbiosis and colonic inflammaging (Shi et al., 2023). Recent reviews characterize *RELMβ* as regulating colonic homeostasis by regulating expression of the *Reg3β/γ* proteins, as well as regulating colonic permeability and inflammation/homeostasis (Pine et al., 2018; Blyth et al., 2020). These data are strongly supported by *RELMβ* KO mouse studies. Hogan et al. (2006) showed that *RELMβ* KO mice have a virtual loss of *Reg3β/g* proteins in the colon as well as 50+ other homeostasis-related genes using microarray, and an increase in intestinal permeability of greater than 300%. Rectal administration of *RELMβ* ameliorates TNBS colitis in mice (Krimi et al., 2008), and rectal administration of butyrate in mice induces *RELMβ* colonic expression and ameliorates colitis (Jiminez et al., 2017). Other recent studies using *RELMβ* KO mice showed these mice had enhanced susceptibility to colonic inflammation, colon cancer, and glucose intolerance. They found *RELMβ* was secreted by colonic goblet cells into the stool and the bloodstream (Wernstedt Asterholm et al., 2016). In another study, rectal delivery of RELMβ in RELMβ KO mice restored colon T-cell function and reduced mucosal pathology (Bergstrom et al., 2015).

Cohoused and germ-free mice fed feces from hREG3A transgenic mice developed less severe DSS colitis (Darnaud et al., 2018). This is an FMT experiment. Considering our data, these findings, and other studies involving AMP KO and rectal treatment cited above, these studies have not considered that in a young-to-old FMT study, the *healthy levels of AMPs in the younger mice stool are also being transferred with FMT*, *especially most likely Reg3β/γ and RELMβ AMPs*. *Conversely*, *an old-to-young FMT would lack the anti-inflammatory and anti-microbial AMPs as well as contain increased Gn dysbiosis. This may be the most impactful concept of our study*. Our study investigated the hypothesis that changes in colonic crypt epithelial cell gene expression with aging might play a key role in aging-related Gn dysbiosis and inflammaging. Our data strongly support this hypothesis but do not absolutely resolve the cause or consequence of AMP loss (DeJong et al., 2020). Our data supports a model in which aging-related loss of colonic AMPs that can suppress Gn bacteria strongly correlates with the loss of suppression of Gn pathobionts and with inflammaging-related changes in colonic gene expression. Alternatively, aging-related losses in genes such as *Ctnnb1* and *Hmgb1* could be driving leaky-gut, AMP expression loss, and colonic inflammaging. However, we noted several KO and recombinant AMPs that support a causative role for AMP loss in promoting Gn dysbiosis and increased inflammaging markers. The increased abundances of Gn bacterial groups correlating with our barrier, senescence, and inflammation group genes (Figure 6) are all subsets of the Gn group correlating with AMP loss. The loss of *RELMβ* appears to play a key role in this model, including early significant loss (61%) at 15 mth (50 yo human), strong correlations with the AMP gene loss (especially *Reg3β/γ*) (Sun et al., 2021), and increased abundances of Gn bacteria (Blyth et al., 2020; Shi et al., 2023). Interestingly, a recent mouse study suggested 10 mth (midlife) as a possible “tipping point” for the aging microbiome that was partially ameliorated with prebiotic feeding (Boehme et al., 2019). Prebiotics promote *Reg3β/γ* and *RELMβ* expression. Our AMP data are also like the model supported by the recent *Drosophila* AMP KO study cited earlier, in which loss of multiple intestinal AMPs resulted in Gn dysbiosis and decreased longevity. Rearing these AMP KO *Drosophila* under germ-free conditions with no intestinal microbiome significantly rescued lifespan, supporting that intestinal AMPs contribute to lifespan through their impact on the microbiome (Hanson and Lemaitre, 2023).

As with any successful study, our data has limitations that raise questions to be answered in future studies. One of our future objectives is to measure the actual AMP protein levels in the blood and stool of aging mice, as well as human aging studies. We are planning future aging studies using this mouse model, in which we will measure protein levels of AMPs in blood and stool, as well as RNA-seq and 16S stool analyses. Stool AMP levels will also be measured in FMT studies. As noted above, FMT of young microbiome to old mice could include healthy levels of AMPs in the young mouse stool, which have potent anti-inflammatory and homeostasis properties as well as anti-Gn microbicidal properties and fewer Gn bacteria (Shin and Seeley, 2019; Sun et al., 2021; Shi et al., 2023). In healthy young mice (and humans), *RELMβ* and *Reg3β/γ* are measurable in the stool and blood with commercial ELISA (Hogan et al., 2006; Wernstedt Asterholm et al., 2016; Shin and Seeley, 2019). High levels of stool *RELMβ* are associated with increased survival in human colon cancer (Zheng et al., 2009). Our study also highlights the potential role of several key non-AMP-related barrier, senescence, and inflammation gene interactions with the microbiome to be investigated in future studies. The findings from this study will be instrumental in informing future animal trials, which will involve a larger sample size necessary to further assess the effects of aging accurately. Additionally, the findings in our aging mouse model will need to be evaluated in human clinical trials to confirm that these aging outcomes are translatable to human health. It is important to note that animal models provide valuable information, but it is well understood that the intestines and the microbiota of each mammalian species are distinct. For microbiome optimization in humans, studies of microbiota-based therapeutic interventions will require extensive studies in clinical trials.

In terms of possible insights into potential therapies reversing aging Gn gut dysbiosis by stimulating intestinal AMP expression, significant are several studies supporting a role for fermentable fiber and probiotics, as well as rectal butyrate, in promoting *Reg3β/γ* (Shin et al., 2022; Rock and Turnbaugh, 2023) and also *RELMβ* AMPs (Monk et al., 2015; Monk et al., 2016; Jiminez et al., 2017; Shi et al., 2023). Both probiotics and prebiotics stimulate increased *Reg3γ* production in the ileum and colon, which, along with *RELMβ*, is also associated with systemic metabolism that is lost in *Reg3γ* and *RELMβ* KO mice (Shi et al., 2023; Shin et al., 2023). Significantly, a high-fat diet has been shown to disrupt metabolism in significant part by having negative effects on intestinal *Reg3γ* and *RELMβ* function (Frazier et al., 2022; Shi et al., 2023). Together, these data support a novel mechanism in the beneficial effects of probiotic and prebiotic high-fiber diets beyond promoting the growth of “good” bacteria but also potentially promoting expression of key intestinal AMPs, including *Reg3β/γ* and *RELMβ* (Fujio et al., 2008; Shin et al., 2023). These data could provide a framework for analyzing microbiota–gut health associations and interactions with the physiological aging process to help scientists further explore and develop microbiota-based therapeutic interventions for older human populations. Translation of laboratory discoveries to the clinic requires clinical trials to demonstrate safety and effectiveness.

In summary, our study represents a significant step forward in understanding the complex mechanisms of colonic gene expression and associated microbiome profiles involved in promoting an increase in the pro-inflammatory gram-negative microbiome associated with aging dysbiosis and colonic inflammaging. As noted above, recent FMT studies in aging and young mice have revealed a potentially critical role for aging-related dysbiosis as a driver of systemic inflammation and aging. Similar studies in *Drosophila* and killifish support a key role for the increase in intestinal Gram-negative pro-inflammatory bacteria with aging (dysbiosis) driving inflammaging.

## Data Availability

The datasets presented in this study can be found in online repositories. The names of the repository/repositories and accession number(s) can be found in the article/Supplementary Material.
