# Supplementary material for: Evidence that the loss of colonic anti-microbial peptides may promote dysbiotic Gram-negative inflammaging-associated bacteria in aging mice
Source: Front Aging. 2024 Mar 4;5:1352299. doi: 10.3389/fragi.2024.1352299 (PMC10945560; doi:10.3389/fragi.2024.1352299)
Supplement: Supplementary file 3 [file Presentation1.pdf]

Supplementary Material

1 Supplementary Data

Supplementary Data Sheets uploaded individually as Microsoft Excel files. Please note there are 14 Supplementary Data Sheets.

2 Supplementary Tables

Supplementary Table 1. Alpha diversity indices of feces within aging mice.

One-Way ANOVA test Shannon: $F_{(2,19)} = 1.763$; p-value = 0.1985

Kruskal-Wallis test Simpson: p-value = 0.1630^{+}

One-Way ANOVA test Observed Features: $F_{(2,19)} = 2.004$; p-value = 0.1623

One-Way ANOVA test Evenness: $F_{(2,19)} = 1.054$; p-value = 0.3679

Mice Comparisons	Diversity Index	Mean ± (SD)	Mean ± (SD)	p-value	q-value
2 Month vs. 15 Month	Shannon	5.79 (0.50)	6.10 (0.54)	0.2626	0.4135
2 Month vs 25 Month	Shannon	5.79 (0.50)	6.32 (0.47)	0.0763	0.2404
15 Month vs 25 Month	Shannon	6.10 (0.54)	6.32 (0.47)	0.3995	0.4194
2 Month vs. 15 Month	Simpson	0.95 (0.01)	0.96 (0.01)	0.2425^{+}	0.3819^{+}
2 Month vs 25 Month	Simpson	0.95 (0.01)	0.97 (0.008)	0.0560^{+}	0.1763^{+}
15 Month vs 25 Month	Simpson	0.96 (0.01)	0.97 (0.008)	0.3747^{+}	0.3935^{+}
2 Month vs. 15 Month	Observed Features	209.83 (65.64)	256.66 (49.48)	0.1493	0.2351
2 Month vs 25 Month	Observed Features	209.83 (65.64)	273.71 (64.90)	0.0671	0.2113
15 Month vs 25 Month	Observed Features	256.66 (49.48)	273.71 (64.90)	0.5739	0.6026
2 Month vs. 15 Month	Evenness	0.75 (0.03)	0.76 (0.04)	0.7314	0.7680
2 Month vs 25 Month	Evenness	0.75 (0.03)	0.78 (0.02)	0.1904	0.4262
15 Month vs 25 Month	Evenness	0.76 (0.04)	0.78 (0.02)	0.2706	0.4262

Alpha diversity values of Shannon's Index, Simpson's Index, Observed Features and Pielou's Evenness examined at the feature level. Rarefaction level at 4,800 sequences per sample. Based on Shapiro-Wilk test of normality, either the parametric One-Way ANOVA or the non-parametric Kruskal-Wallis test ($^{+}$) were conducted within these alpha diversity indices. Results corrected for multiple comparisons using the Benjamini-Hochberg method (q-value). *Significance: p-value < 0.05; q-value < 0.05. Mean index score and standard deviation (SD) displayed. Group sample sizes: 2 Month (n = 6), 15 Month (n = 9), and 25 Month (n = 7).

Supplementary Table 2. Anti-Microbial Peptides and Regulator Differentially Expressed Genes between Aging Mice Groups.								
**Gene ID**	**Gene Description and Accession Number**	**Regulation Up/Down**	**logFC**	**logCPM**	**2 Month**	**15 Month**	**p-value**	**q-value**
Ang4	angiogenin, ribonuclease A family, member 4 [Source:MGI Symbol;Acc:MGI:2656551]	Down	-3.396	7.715	7292.600	861.667	**1.329E-07***	**3.636E-05***
Reg3b	regenerating islet-derived 3 beta [Source:MGI Symbol;Acc:MGI:97478]	Down	-2.366	7.163	4269.600	1660.500	**2.184E-04***	**9.868E-03***
Retnlb	resistin like beta [Source:MGI Symbol;Acc:MGI:1888505]	Down	-1.867	6.918	3493.600	1346.833	**0.001***	**0.024***
Lypd8l	LY6/PLAUR domain containing 8 like [Source:MGI Symbol;Acc:MGI:1925604]	Down	-1.263	6.480	2271.000	1762.833	**0.003***	0.051
S100a10	S100 calcium binding protein A10 (calpactin) [Source:MGI Symbol;Acc:MGI:1339468]	Down	-0.687	8.900	11078.600	11043.833	**0.005***	0.074
Nlrp6	NLR family, pyrin domain containing 6 [Source:MGI Symbol;Acc:MGI:2141990]	Up	0.685	7.795	2907.200	7820.167	**0.015***	0.127
S100g	S100 calcium binding protein G [Source:MGI Symbol;Acc:MGI:104528]	Down	-1.665	6.283	2250.000	1452.500	**0.016***	0.131
Lcn2	lipocalin 2 [Source:MGI Symbol;Acc:MGI:96757]	Up	7.164	-1.050	0.000	18.500	**0.041***	0.226
Sprr2a3	small proline-rich protein 2A3 [Source:MGI Symbol;Acc:MGI:3845028]	Down	-0.787	8.598	9251.600	7723.000	**0.042***	0.227
Extl3	exostosin-like glycosyltransferase 3 [Source:MGI Symbol;Acc:MGI:1860765]	Up	2.309	3.800	91.800	615.500	0.129	0.435
Reg3g	regenerating islet-derived 3 gamma [Source:MGI Symbol;Acc:MGI:109406]	Down	-0.902	6.002	1460.400	1385.167	0.148	0.474
Cdx2	caudal type homeobox 2 [Source:MGI Symbol;Acc:MGI:88361]	Up	0.390	7.843	3584.200	7027.333	0.240	0.629
Myd88	myeloid differentiation primary response gene 88 [Source:MGI Symbol;Acc:MGI:108005]	Up	0.279	4.949	421.000	968.167	0.638	0.920
Sprr2a2	small proline-rich protein 2A2 [Source:MGI Symbol;Acc:MGI:3845026]	Up	0.226	5.386	844.200	972.000	0.664	0.930
Sprr2a1	small proline-rich protein 2A1 [Source:MGI Symbol;Acc:MGI:1330350]	Up	0.347	4.812	549.400	569.333	0.705	0.939
Lypd8	LY6/PLAUR domain containing 8 [Source:MGI Symbol;Acc:MGI:1917413]	Up	0.024	12.160	78146.400	128513.167	0.926	0.987
Defb37	defensin beta 37 [Source:MGI Symbol;Acc:MGI:2672966]	Up	0.022	2.387	90.200	131.000	1.000	1.000

Gene ID	Gene Description and Accession Number	Regulation Up/Down	logFC	logCPM	2 Month	25 Month	p-value	q-value
Sprr2a3	small proline-rich protein 2A3 [Source:MGI Symbol;Acc:MGI:3845028]	Down	-1.947	8.262	9251.600	3787.667	**7.432E-13***	**3.069E-10***
Ang4	angiogenin, ribonuclease A family, member 4 [Source:MGI Symbol;Acc:MGI:2656551]	Down	-4.837	7.663	7292.600	498.667	**5.793E-10***	**8.339E-08***
S100g	S100 calcium binding protein G [Source:MGI Symbol;Acc:MGI:104528]	Down	-3.023	6.054	2250.000	392.833	**6.088E-08***	**3.972E-06***
Lypd8l	LY6/PLAUR domain containing 8 like [Source:MGI Symbol;Acc:MGI:1925604]	Down	-2.287	6.243	2271.000	725.333	**9.083E-08***	**5.524E-06***
Retnlb	resistin like beta [Source:MGI Symbol;Acc:MGI:1888505]	Down	-2.931	6.763	3493.600	703.667	**2.395E-06***	**7.374E-05***
S100a10	S100 calcium binding protein A10 (calpactin) [Source:MGI Symbol;Acc:MGI:1339468]	Down	-0.962	8.822	11078.600	9428.833	**6.665E-06***	**1.634E-04***
Defb45	defensin beta 45 [Source:MGI Symbol;Acc:MGI:3650541]	Up	9.661	1.273	0.000	171.833	**3.406E-04***	**0.004***
Reg3b	regenerating islet-derived 3 beta [Source:MGI Symbol;Acc:MGI:97478]	Down	-3.589	7.038	4269.600	619.500	**4.894E-04***	**0.006***
Lypd8	LY6/PLAUR domain containing 8 [Source:MGI Symbol;Acc:MGI:1917413]	Up	0.692	11.867	78146.400	89254.500	**0.001***	**0.011***
Sprr2a2	small proline-rich protein 2A2 [Source:MGI Symbol;Acc:MGI:3845026]	Down	-1.674	4.865	844.200	397.333	**0.001***	**0.013***
Lcn2	lipocalin 2 [Source:MGI Symbol;Acc:MGI:96757]	Up	8.604	0.270	0.000	60.500	**0.004***	**0.031***
Reg3g	regenerating islet-derived 3 gamma [Source:MGI Symbol;Acc:MGI:109406]	Down	-2.630	5.583	1460.400	374.833	**0.004***	**0.032***
Sprr2a1	small proline-rich protein 2A1 [Source:MGI Symbol;Acc:MGI:1330350]	Down	-1.690	4.357	549.400	274.000	0.091	0.287
Extl3	exostosin-like glycosyltransferase 3 [Source:MGI Symbol;Acc:MGI:1860765]	Up	2.413	3.934	91.800	708.000	0.117	0.341
Ltf	lactotransferrin [Source:MGI Symbol;Acc:MGI:96837]	Up	5.871	-1.977	0.000	8.500	0.153	0.393
Cdx2	caudal type homeobox 2 [Source:MGI Symbol;Acc:MGI:88361]	Up	0.293	7.494	3584.200	4482.167	0.306	0.596
Myd88	myeloid differentiation primary response gene 88 [Source:MGI Symbol;Acc:MGI:108005]	Up	0.319	4.646	421.000	635.333	0.543	0.795
Defb37	defensin beta 37 [Source:MGI Symbol;Acc:MGI:2672966]	Down	-0.795	2.050	90.200	83.167	0.658	0.865
Nlrp6	NLR family, pyrin domain containing 6 [Source:MGI Symbol;Acc:MGI:2141990]	Up	0.023	7.422	2907.200	4879.833	0.933	0.978

Gene ID	Gene Description and Accession Number	Regulation Up/Down	logFC	logCPM	15 Month	25 Month	p-value	q-value
Sprr2a3	small proline-rich protein 2A3 [Source:MGI Symbol;Acc:MGI:3845028]	Down	-1.176	7.794	7723.000	3787.667	**3.556E-04***	0.101
Sprr2a2	small proline-rich protein 2A2 [Source:MGI Symbol;Acc:MGI:3845026]	Down	-1.463	4.804	972.000	397.333	**4.269E-04***	0.110
Nlrp6	NLR family, pyrin domain containing 6 [Source:MGI Symbol;Acc:MGI:2141990]	Down	-0.690	7.842	7820.167	4879.833	**0.003***	0.270
Lypd8	LY6/PLAUR domain containing 8 [Source:MGI Symbol;Acc:MGI:1917413]	Down	-0.685	11.924	128513.167	89254.500	**0.007***	0.348
Sprr2a1	small proline-rich protein 2A1 [Source:MGI Symbol;Acc:MGI:1330350]	Down	-1.346	4.199	569.333	274.000	**0.011***	0.421
Lypd8l	LY6/PLAUR domain containing 8 like [Source:MGI Symbol;Acc:MGI:1925604]	Down	-1.050	5.418	1762.833	725.333	**0.014***	0.447
Cdx2	caudal type homeobox 2 [Source:MGI Symbol;Acc:MGI:88361]	Down	-0.708	7.777	7027.333	4482.167	**0.021***	0.507
Retnlb	resistin like beta [Source:MGI Symbol;Acc:MGI:1888505]	Down	-1.065	5.421	1346.833	703.667	**0.032***	0.588
Ang4	angiogenin, ribonuclease A family, member 4 [Source:MGI Symbol;Acc:MGI:2656551]	Down	-1.441	4.832	861.667	498.667	**0.035***	0.603
Reg3g	regenerating islet-derived 3 gamma [Source:MGI Symbol;Acc:MGI:109406]	Down	-1.737	4.975	1385.167	374.833	**0.040***	0.627
S100g	S100 calcium binding protein G [Source:MGI Symbol;Acc:MGI:104528]	Down	-1.381	4.846	1452.500	392.833	0.056	0.666
Myd88	myeloid differentiation primary response gene 88 [Source:MGI Symbol;Acc:MGI:108005]	Down	-0.627	4.866	968.167	635.333	0.058	0.672
Ltf	lactotransferrin [Source:MGI Symbol;Acc:MGI:96837]	Up	6.153	-2.152	0.000	8.500	0.068	0.683
S100a10	S100 calcium binding protein A10 (calpactin) [Source:MGI Symbol;Acc:MGI:1339468]	Down	-0.294	8.487	11043.833	9428.833	0.103	0.761
Reg3b	regenerating islet-derived 3 beta [Source:MGI Symbol;Acc:MGI:97478]	Down	-1.240	5.220	1660.500	619.500	0.182	0.888
Defb45	defensin beta 45 [Source:MGI Symbol;Acc:MGI:3650541]	Up	1.918	1.505	36.000	171.833	0.288	0.956
Defb37	defensin beta 37 [Source:MGI Symbol;Acc:MGI:2672966]	Down	-0.828	2.115	131.000	83.167	0.407	0.990
Lcn2	lipocalin 2 [Source:MGI Symbol;Acc:MGI:96757]	Up	1.429	0.577	18.500	60.500	0.443	0.995
Extl3	exostosin-like glycosyltransferase 3 [Source:MGI Symbol;Acc:MGI:1860765]	Up	0.098	4.575	615.500	708.000	0.736	1.000

Edge-R conducted to detect RNASeq differential gene expression levels between aging mice groups. Results corrected for multiple comparisons using the Benjamini-Hochberg method (q-value). *Significance: p-value < 0.05; q-value < 0.05. Mean gene expression levels per group displayed. Number of mice per group: 2 Month (n = 5), 15 Month (n = 6), and 25 Month (n = 6). logFC = log fold change; logCPM = log counts per million.

Supplementary Table 3. *RELM-ß* and *Reg3b* Real-Time Polymerase Chain Reaction Gene Expression Values of Aging Mice.

RELM-ß (resistin-like molecule beta, Retnlß) **Kruskal-Wallis: p-value = 0.0015*⁺**				
**Mice Comparisons**	**Mean ± (SD)**	**Mean ± (SD)**	**p-value**	**q-value**
2 Month vs. 15 Month	0.844 (0.67)	0.377 (0.15)	0.1936⁺	0.1335⁺
2 Month vs 25 Month	0.844 (0.67)	0.202 (0.12)	**0.0019*⁺**	**0.0040*⁺**
15 Month vs 25 Month	0.377 (0.15)	0.202 (0.12)	0.1190⁺	0.1250⁺
Reg3ß (Regenerating islet-derived 3 beta) **One-Way Analysis of Variance: $F_{(2, 14)}$ = 4.551, p-value = 0.0300***				
**Mice Comparisons**	**Mean ± (SD)**	**Mean ± (SD)**	**p-value**	**q-value**
2 Month vs. 15 Month	1.048 (0.38)	0.825 (0.27)	0.3048	0.2134
2 Month vs 25 Month	1.048 (0.38)	0.453 (0.35)	**0.0098***	**0.0205***
15 Month vs 25 Month	0.825 (0.27)	0.453 (0.35)	0.0960	0.1008

Based on Shapiro-Wilk test of normality, the parametric one-way analysis of variance (ANOVA) test or non-parametric Kruskal-Wallis test (⁺) were conducted on *RELM-ß* and *Reg3b* qPCR expression values between aging mice. Results corrected for multiple comparisons using the Benjamini-Hochberg method (q-value). *Significance: p-value < 0.05; q-value < 0.05. Mean index score and standard deviation (SD) displayed. Number of values per group: 2 Month (n = 5), 15 Month (n = 6), and 25 Month (n = 6).

Supplementary Table 4. Colonic Barrier Mucus, Shaping and Junction Differentially Expressed Genes between Aging Mice Groups.

Gene ID	Gene Description and Accession Number	Regulation Up/Down	logFC	logCPM	2 Month	15 Month	p-value	q-value
Muc3	mucin 3, intestinal [Source:MGI Symbol;Acc:MGI:1203527]	Up	1.727	10.230	9362.600	50949.000	**7.317E-07***	**1.545E-04***
Clca1	chloride channel accessory 1 [Source:MGI Symbol;Acc:MGI:1346342]	Down	-1.670	10.353	36206.600	18055.833	**5.159E-05***	**0.004***
HMGB1	high mobility group box 1 [Source:MGI Symbol;Acc:MGI:96113]	Down	-0.945	8.479	8754.200	7464.833	**7.268E-05***	**0.005***
Hsp90b1	heat shock protein 90, beta (Grp94), member 1 [Source:MGI Symbol;Acc:MGI:98817]	Up	1.130	8.513	4076.800	14506.500	**1.073E-04***	**0.006***
Clca4a	chloride channel accessory 4A [Source:MGI Symbol;Acc:MGI:2139744]	Down	-1.703	6.257	2392.200	1052.500	**1.312E-04***	**0.007***
Cldn15	claudin 15 [Source:MGI Symbol;Acc:MGI:1913103]	Up	1.450	7.512	1683.600	8469.667	**1.690E-04***	**0.008***
Muc3a	mucin 3A, cell surface associated [Source:MGI Symbol;Acc:MGI:3588263]	Up	1.380	7.644	1696.000	8536.833	**1.884E-04***	**0.009***
Reg3b	regenerating islet-derived 3 beta [Source:MGI Symbol;Acc:MGI:97478]	Down	-2.366	7.163	4269.600	1660.500	**2.184E-04***	**0.010***
Tff3	trefoil factor 3, intestinal [Source:MGI Symbol;Acc:MGI:104638]	Down	-1.158	9.120	13792.200	11328.833	**0.001***	**0.021***
Ctnnb1	catenin (cadherin associated protein), beta 1 [Source:MGI Symbol;Acc:MGI:88276]	Down	-1.078	10.036	26508.200	18896.667	**0.001***	**0.021***
Retnlb	resistin like beta [Source:MGI Symbol;Acc:MGI:1888505]	Down	-1.867	6.918	3493.600	1346.833	**0.001***	**0.024***
Muc2	mucin 2 [Source:MGI Symbol;Acc:MGI:1339364]	Up	1.093	13.358	109142.800	442098.667	**0.001***	**0.030***
Tjp1	tight junction protein 1 [Source:MGI Symbol;Acc:MGI:98759]	Down	-1.172	6.447	2231.200	1565.167	**0.002***	**0.035***
Cldn14	claudin 14 [Source:MGI Symbol;Acc:MGI:1860425]	Up	9.480	1.065	0.000	118.333	**0.003***	0.053
Tjp3	tight junction protein 3 [Source:MGI Symbol;Acc:MGI:1351650]	Up	0.683	8.307	4154.600	11045.000	**0.009***	0.097
Cldn4	claudin 4 [Source:MGI Symbol;Acc:MGI:1313314]	Down	-2.012	4.073	533.400	218.167	**0.018***	0.142
Cdh1	cadherin 1 [Source:MGI Symbol;Acc:MGI:88354]	Up	0.531	8.752	6200.200	14758.167	**0.035***	0.209
Muc1	mucin 1, transmembrane [Source:MGI Symbol;Acc:MGI:97231]	Up	5.380	0.581	1.400	85.667	**0.043***	0.230

Cldn7	claudin 7 [Source:MGI Symbol;Acc:MGI:1859285]	Up	0.497	9.332	8876.600	22702.167	**0.045***	0.237
Clca2	chloride channel accessory 2 [Source:MGI Symbol;Acc:MGI:2139758]	Down	-4.188	1.237	96.600	6.833	0.113	0.404
Reg3g	regenerating islet-derived 3 gamma [Source:MGI Symbol;Acc:MGI:109406]	Down	-0.902	6.002	1460.400	1385.167	0.148	0.474
Cldn23	claudin 23 [Source:MGI Symbol;Acc:MGI:1919158]	Up	0.893	4.185	389.600	393.667	0.224	0.608
Piezo1	piezo-type mechanosensitive ion channel component 1 [Source:MGI Symbol;Acc:MGI:3603204]	Up	1.482	3.466	96.000	462.333	0.293	0.695
Cldn2	claudin 2 [Source:MGI Symbol;Acc:MGI:1276110]	Up	0.243	6.701	1532.800	3263.667	0.485	0.843
Ocln	occludin [Source:MGI Symbol;Acc:MGI:106183]	Up	0.236	5.193	703.200	1082.500	0.602	0.906
Pparg	peroxisome proliferator activated receptor gamma [Source:MGI Symbol;Acc:MGI:97747]	Up	0.049	6.759	1904.400	3119.833	0.882	0.977
**Gene ID**	**Gene Description and Accession Number**	**Regulation Up/Down**	**logFC**	**logCPM**	**2 Month**	**25 Month**	**p-value**	**q-value**
Ctnnb1	catenin (cadherin associated protein), beta 1 [Source:MGI Symbol;Acc:MGI:88276]	Down	-1.684	9.878	26508.200	13110.500	**6.015E-14***	**2.860E-11***
Clca4a	chloride channel accessory 4A [Source:MGI Symbol;Acc:MGI:2139744]	Down	-2.312	6.152	2392.200	691.667	**4.792E-08***	**3.327E-06***
Clca1	chloride channel accessory 1 [Source:MGI Symbol;Acc:MGI:1346342]	Down	-1.858	10.341	36206.600	18565.833	**5.749E-08***	**3.798E-06***
Cldn15	claudin 15 [Source:MGI Symbol;Acc:MGI:1913103]	Up	1.393	7.501	1683.600	7280.667	**1.960E-07***	**1.002E-05***
Cldn14	claudin 14 [Source:MGI Symbol;Acc:MGI:1860425]	Up	10.865	2.445	0.000	361.500	**3.935E-07***	**1.736E-05***
HMGB1	high mobility group box 1 [Source:MGI Symbol;Acc:MGI:96113]	Down	-0.907	8.525	8754.200	7656.167	**2.094E-06***	**6.614E-05***
Retnlb	resistin like beta [Source:MGI Symbol;Acc:MGI:1888505]	Down	-2.931	6.763	3493.600	703.667	**2.395E-06***	**7.374E-05***
Muc1	mucin 1, transmembrane [Source:MGI Symbol;Acc:MGI:97231]	Up	7.338	2.584	1.400	316.833	**5.613E-06***	**1.439E-04***
Muc3a	mucin 3A, cell surface associated [Source:MGI Symbol;Acc:MGI:3588263]	Up	1.408	7.684	1696.000	7990.000	**2.175E-05***	**4.363E-04***
Hsp90b1	heat shock protein 90, beta (Grp94), member 1 [Source:MGI Symbol;Acc:MGI:98817]	Up	0.944	8.416	4076.800	13029.500	**2.882E-04***	**0.004***

Muc2	mucin 2 [Source:MGI Symbol;Acc:MGI:1339364]	Up	1.250	13.494	109142.800	421131.333	**3.733E-04***	**0.005***
Reg3b	regenerating islet-derived 3 beta [Source:MGI Symbol;Acc:MGI:97478]	Down	-3.589	7.038	4269.600	619.500	**4.894E-04***	**0.006***
Tjp1	tight junction protein 1 [Source:MGI Symbol;Acc:MGI:98759]	Down	-1.050	6.522	2231.200	1675.500	**0.001***	**0.006***
Clca2	chloride channel accessory 2 [Source:MGI Symbol;Acc:MGI:2139758]	Down	-9.844	1.188	96.600	0.000	**0.002***	**0.019***
Reg3g	regenerating islet-derived 3 gamma [Source:MGI Symbol;Acc:MGI:109406]	Down	-2.630	5.583	1460.400	374.833	**0.004***	**0.032***
Cldn4	claudin 4 [Source:MGI Symbol;Acc:MGI:1313314]	Down	-2.330	4.035	533.400	156.833	**0.006***	**0.041***
Tjp3	tight junction protein 3 [Source:MGI Symbol;Acc:MGI:1351650]	Up	0.562	8.256	4154.600	10290.000	**0.007***	**0.044***
Muc3	mucin 3, intestinal [Source:MGI Symbol;Acc:MGI:1203527]	Up	1.007	9.719	9362.600	37180.167	**0.008***	**0.049***
Cldn23	claudin 23 [Source:MGI Symbol;Acc:MGI:1919158]	Down	-1.704	3.952	389.600	211.167	**0.012***	0.067
Pparg	peroxisome proliferator activated receptor gamma [Source:MGI Symbol;Acc:MGI:97747]	Down	-0.764	6.451	1904.400	1830.833	**0.020***	0.098
Tff3	trefoil factor 3, intestinal [Source:MGI Symbol;Acc:MGI:104638]	Up	0.595	9.372	13792.200	17267.333	**0.025***	0.118
Ocln	occludin [Source:MGI Symbol;Acc:MGI:106183]	Up	0.203	5.240	703.200	1153.833	0.631	0.849
Cldn7	claudin 7 [Source:MGI Symbol;Acc:MGI:1859285]	Up	0.093	9.118	8876.600	17479.167	0.670	0.871
Piezo1	piezo-type mechanosensitive ion channel component 1 [Source:MGI Symbol;Acc:MGI:3603204]	Up	0.491	2.285	96.000	125.833	0.710	0.889
Cdh1	cadherin 1 [Source:MGI Symbol;Acc:MGI:88354]	Up	0.039	8.449	6200.200	10086.500	0.855	0.950
Cldn2	claudin 2 [Source:MGI Symbol;Acc:MGI:1276110]	Up	0.014	6.596	1532.800	2945.833	0.970	0.991
**Gene ID**	**Gene Description and Accession Number**	**Regulation Up/Down**	**logFC**	**logCPM**	**15 Month**	**25 Month**	**p-value**	**q-value**
Piezo1	piezo-type mechanosensitive ion channel component 1 [Source:MGI Symbol;Acc:MGI:3603204]	Down	-1.983	3.368	462.333	125.833	**3.888E-04***	0.107

Cdh1	cadherin 1 [Source:MGI Symbol;Acc:MGI:88354]	Down	-0.592	8.784	14758.167	10086.500	**0.001***	0.121
Ctnnb1	catenin (cadherin associated protein), beta 1 [Source:MGI Symbol;Acc:MGI:88276]	Down	-0.626	9.246	18896.667	13110.500	**0.020***	0.502
Pparg	peroxisome proliferator activated receptor gamma [Source:MGI Symbol;Acc:MGI:97747]	Down	-0.736	6.495	3119.833	1830.833	**0.022***	0.520
Cldn7	claudin 7 [Source:MGI Symbol;Acc:MGI:1859285]	Down	-0.429	9.418	22702.167	17479.167	**0.024***	0.539
Retnlb	resistin like beta [Source:MGI Symbol;Acc:MGI:1888505]	Down	-1.065	5.421	1346.833	703.667	**0.032***	0.588
Reg3g	regenerating islet-derived 3 gamma [Source:MGI Symbol;Acc:MGI:109406]	Down	-1.737	4.975	1385.167	374.833	**0.040***	0.627
Muc1	mucin 1, transmembrane [Source:MGI Symbol;Acc:MGI:97231]	Up	2.045	2.791	85.667	316.833	**0.047***	0.642
Muc3	mucin 3, intestinal [Source:MGI Symbol;Acc:MGI:1203527]	Down	-0.734	10.538	50949.000	37180.167	0.059	0.672
Cldn23	claudin 23 [Source:MGI Symbol;Acc:MGI:1919158]	Down	-0.833	3.439	393.667	211.167	0.067	0.683
Clca2	chloride channel accessory 2 [Source:MGI Symbol;Acc:MGI:2139758]	Down	-5.957	-2.301	6.833	0.000	0.073	0.688
Clca4a	chloride channel accessory 4A [Source:MGI Symbol;Acc:MGI:2139744]	Down	-0.625	5.043	1052.500	691.667	0.073	0.689
Tff3	trefoil factor 3, intestinal [Source:MGI Symbol;Acc:MGI:104638]	Up	0.542	8.853	11328.833	17267.333	0.092	0.735
Reg3b	regenerating islet-derived 3 beta [Source:MGI Symbol;Acc:MGI:97478]	Down	-1.240	5.220	1660.500	619.500	0.182	0.888
Cldn14	claudin 14 [Source:MGI Symbol;Acc:MGI:1860425]	Up	1.371	2.812	118.333	361.500	0.229	0.923
Cldn2	claudin 2 [Source:MGI Symbol;Acc:MGI:1276110]	Down	-0.255	6.763	3263.667	2945.833	0.247	0.934
Hsp90b1	heat shock protein 90, beta (Grp94), member 1 [Source:MGI Symbol;Acc:MGI:98817]	Down	-0.203	8.903	14506.500	13029.500	0.295	0.960
Cldn4	claudin 4 [Source:MGI Symbol;Acc:MGI:1313314]	Down	-0.337	2.753	218.167	156.833	0.562	1.000
Clca1	chloride channel accessory 1 [Source:MGI Symbol;Acc:MGI:1346342]	Up	0.201	9.341	18055.833	18565.833	0.602	1.000
Muc2	mucin 2 [Source:MGI Symbol;Acc:MGI:1339364]	Down	-0.121	13.898	442098.667	421131.333	0.708	1.000
Tjp1	tight junction protein 1 [Source:MGI Symbol;Acc:MGI:98759]	Up	0.095	5.929	1565.167	1675.500	0.762	1.000

Cldn15	claudin 15 [Source:MGI Symbol;Acc:MGI:1913103]	Down	-0.080	8.043	8469.667	7280.667	0.792	1.000
HMGB1	high mobility group box 1 [Source:MGI Symbol;Acc:MGI:96113]	Up	0.015	8.060	7464.833	7656.167	0.929	1.000
Muc3a	mucin 3A, cell surface associated [Source:MGI Symbol;Acc:MGI:3588263]	Down	-0.011	8.193	8536.833	7990.000	0.964	1.000
Ocln	occludin [Source:MGI Symbol;Acc:MGI:106183]	Up	0.014	5.167	1082.500	1153.833	0.966	1.000

Edge-R conducted to detect RNASeq differential gene expression levels between aging mice groups. Results corrected for multiple comparisons using the Benjamini-Hochberg method (q-value). *Significance: p-value < 0.05; q-value < 0.05. Mean gene expression levels per group displayed. Number of mice per group: 2 Month (n = 5), 15 Month (n = 6), and 25 Month (n = 6). logFC = log fold change; logCPM = log counts per million.

Supplementary Table 5. Senescence Differentially Expressed Genes between Aging Mice Groups.								
**Gene ID**	**Gene Description and Accession Number**	**Regulation Up/Down**	**logFC**	**logCPM**	**2 Month**	**15 Month**	**p-value**	**q-value**
HMGB1	high mobility group box 1 [Source:MGI Symbol;Acc:MGI:96113]	Down	-0.945	8.479	8754.200	7464.833	**7.27E-05***	**0.005***
Hsp90b1	heat shock protein 90, beta (Grp94), member 1 [Source:MGI Symbol;Acc:MGI:98817]	Up	1.130	8.513	4076.800	14506.500	**0.0001***	**0.006***
Dkkl1	dickkopf-like 1 [Source:MGI Symbol;Acc:MGI:1354963]	Up	9.008	0.613	0.000	76.333	**0.0003***	**0.014***
Ghr	growth hormone receptor [Source:MGI Symbol;Acc:MGI:95708]	Up	9.872	1.439	0.000	114.667	**0.0004***	**0.016***
Grn	granulin [Source:MGI Symbol;Acc:MGI:95832]	Up	1.407	6.388	702.400	3778.167	**0.0004***	**0.016***
Fzd5	frizzled class receptor 5 [Source:MGI Symbol;Acc:MGI:108571]	Up	1.636	6.093	506.000	2977.333	**0.0005***	**0.019***
Mt1	metallothionein 1 [Source:MGI Symbol;Acc:MGI:97171]	Up	1.316	7.459	1871.600	7754.833	**0.0005***	**0.020***
Ctnnb1	catenin (cadherin associated protein), beta 1 [Source:MGI Symbol;Acc:MGI:88276]	Down	-1.078	10.036	26508.200	18896.667	**0.0006***	**0.021***
Igfbp5	insulin-like growth factor binding protein 5 [Source:MGI Symbol;Acc:MGI:96440]	Up	7.610	0.872	0.200	66.000	**0.0007***	**0.023***
Vwf	Von Willebrand factor [Source:MGI Symbol;Acc:MGI:98941]	Up	9.121	0.723	0.000	93.333	**0.0007***	**0.023***
Wnt6	wingless-type MMTV integration site family, member 6 [Source:MGI Symbol;Acc:MGI:98960]	Up	7.977	-0.338	0.000	35.333	**0.003***	0.051
Fzd9	frizzled class receptor 9 [Source:MGI Symbol;Acc:MGI:1313278]	Up	8.969	0.578	0.000	78.167	**0.004***	0.061
Fzd4	frizzled class receptor 4 [Source:MGI Symbol;Acc:MGI:108520]	Up	8.684	0.313	0.000	74.000	**0.005***	0.069
Mmp7	matrix metallopeptidase 7 [Source:MGI Symbol;Acc:MGI:103189]	Up	8.821	0.440	0.000	70.500	**0.005***	0.073
Ctsb	cathepsin B [Source:MGI Symbol;Acc:MGI:88561]	Up	0.705	8.324	4160.800	11455.833	**0.014***	0.121
Xpa	xeroderma pigmentosum, complementation group A [Source:MGI Symbol;Acc:MGI:99135]	Down	-1.389	4.838	705.200	513.667	**0.015***	0.128
Ccl25	chemokine (C-C motif) ligand 25 [Source:MGI Symbol;Acc:MGI:1099448]	Down	-2.287	3.795	384.800	140.000	**0.017***	0.137
Nudt1	nudix (nucleoside diphosphate linked moiety X)-type motif 1 [Source:MGI Symbol;Acc:MGI:109280]	Up	2.057	4.502	161.000	1112.667	**0.019***	0.150

Tnfrsf11b	tumor necrosis factor receptor superfamily, member 11b (osteoprotegerin) [Source:MGI Symbol;Acc:MGI:109587]	Up	6.324	-1.687	0.000	14.500	**0.021***	0.156
Stub1	STIP1 homology and U-Box containing protein 1 [Source:MGI Symbol;Acc:MGI:1891731]	Up	0.835	5.787	697.600	2082.500	**0.021***	0.157
Mif	macrophage migration inhibitory factor (glycosylation-inhibiting factor) [Source:MGI Symbol;Acc:MGI:96982]	Up	0.994	6.429	1040.000	3629.333	**0.021***	0.158
Mmp15	matrix metallopeptidase 15 [Source:MGI Symbol;Acc:MGI:109320]	Up	0.854	6.946	1576.600	4668.833	**0.023***	0.167
Jund	jun D proto-oncogene [Source:MGI Symbol;Acc:MGI:96648]	Up	0.590	8.522	7849.800	9260.167	**0.025***	0.173
Irs1	insulin receptor substrate 1 [Source:MGI Symbol;Acc:MGI:99454]	Up	7.980	-0.329	0.000	42.000	**0.027***	0.184
Txn1	thioredoxin 1 [Source:MGI Symbol;Acc:MGI:98874]	Up	0.558	9.183	12493.600	14813.167	**0.036***	0.211
Lcn2	lipocalin 2 [Source:MGI Symbol;Acc:MGI:96757]	Up	7.164	-1.050	0.000	18.500	**0.041***	0.226
Mmp14	matrix metallopeptidase 14 (membrane-inserted) [Source:MGI Symbol;Acc:MGI:101900]	Up	1.056	6.410	821.800	3628.000	**0.047***	0.242
Wnt2b	wingless-type MMTV integration site family, member 2B [Source:MGI Symbol;Acc:MGI:1261834]	Up	5.949	-1.958	0.000	9.500	0.055	0.267
Il18	interleukin 18 [Source:MGI Symbol;Acc:MGI:107936]	Up	0.502	5.535	625.800	1654.500	0.337	0.737
Kitl	kit ligand [Source:MGI Symbol;Acc:MGI:96974]	Up	0.441	5.056	688.200	742.000	0.416	0.801
Dvl1	dishevelled segment polarity protein 1 [Source:MGI Symbol;Acc:MGI:94941]	Up	0.160	5.317	670.200	1330.667	0.786	0.958
**Gene ID**	**Gene Description and Accession Number**	**Regulation Up/Down**	**logFC**	**logCPM**	**2 Month**	**25 Month**	**p-value**	**q-value**
Ctnnb1	catenin (cadherin associated protein), beta 1 [Source:MGI Symbol;Acc:MGI:88276]	Down	-1.684	9.878	26508.200	13110.500	**6.01E-14***	**2.86E-11***
Vwf	Von Willebrand factor [Source:MGI Symbol;Acc:MGI:98941]	Up	10.722	2.304	0.000	251.000	**1.68E-09***	**2.09E-07***
Fzd9	frizzled class receptor 9 [Source:MGI Symbol;Acc:MGI:1313278]	Up	10.453	2.041	0.000	212.667	**1.53E-08***	**1.34E-06***
Mt1	metallothionein 1 [Source:MGI Symbol;Acc:MGI:97171]	Up	2.132	8.137	1871.600	11532.167	**2.51E-07***	**1.22E-05***

Ghr	growth hormone receptor [Source:MGI Symbol;Acc:MGI:95708]	Up	10.284	1.876	0.000	228.500	**6.91E-07***	**2.84E-05***
Igfbp5	insulin-like growth factor binding protein 5 [Source:MGI Symbol;Acc:MGI:96440]	Up	8.591	1.867	0.200	179.000	**1.85E-06***	**6.04E-05***
Hmgb1	high mobility group box 1 [Source:MGI Symbol;Acc:MGI:96113]	Down	-0.907	8.525	8754.200	7656.167	**2.09E-06***	**6.61E-05***
Fzd4	frizzled class receptor 4 [Source:MGI Symbol;Acc:MGI:108520]	Up	9.115	0.748	0.000	79.333	**2.69E-06***	**8.06E-05***
Mmp7	matrix metallopeptidase 7 [Source:MGI Symbol;Acc:MGI:103189]	Up	9.615	1.227	0.000	133.667	**4.53E-06***	**0.0001***
Cav1^{+}	caveolin 1, caveolae protein [Source:MGI Symbol;Acc:MGI:102709]	Up	8.845	0.494	0.000	65.667	**4.66E-06***	**0.0001***
Hsp90b1	heat shock protein 90, beta (Grp94), member 1 [Source:MGI Symbol;Acc:MGI:98817]	Up	0.944	8.416	4076.800	13029.500	**0.0002***	**0.004***
Wnt2b	wingless-type MMTV integration site family, member 2B [Source:MGI Symbol;Acc:MGI:1261834]	Up	7.997	-0.285	0.000	38.667	**0.0004***	**0.006***
Mmp14	matrix metallopeptidase 14 (membrane-inserted) [Source:MGI Symbol;Acc:MGI:101900]	Up	1.459	6.718	821.800	4426.000	**0.001***	**0.008***
Jund	jun D proto-oncogene [Source:MGI Symbol;Acc:MGI:96648]	Up	0.690	8.506	7849.800	8096.667	**0.001***	**0.009***
Grn	granulin [Source:MGI Symbol;Acc:MGI:95832]	Up	1.088	6.173	702.400	2934.333	**0.002***	**0.017***
Wnt6	wingless-type MMTV integration site family, member 6 [Source:MGI Symbol;Acc:MGI:98960]	Up	8.028	-0.258	0.000	35.333	**0.002***	**0.018***
Dkkl1	dickkopf-like 1 [Source:MGI Symbol;Acc:MGI:1354963]	Up	7.123	-1.039	0.000	19.333	**0.002***	**0.019***
Cdkn1a^{+}	cyclin-dependent kinase inhibitor 1A (P21) [Source:MGI Symbol;Acc:MGI:104556]	Down	-0.856	7.458	4052.000	3469.667	**0.002***	**0.019***
Lmna^{+}	lamin A [Source:MGI Symbol;Acc:MGI:96794]	Up	0.648	8.579	5279.200	13839.333	**0.002***	**0.021***
Eps8^{+}	epidermal growth factor receptor pathway substrate 8 [Source:MGI Symbol;Acc:MGI:104684]	Down	-1.007	6.093	1686.400	1280.500	**0.003***	**0.025***
Mmp15	matrix metallopeptidase 15 [Source:MGI Symbol;Acc:MGI:109320]	Up	1.123	7.171	1576.600	5363.833	**0.003***	**0.025***
Lcn2	lipocalin 2 [Source:MGI Symbol;Acc:MGI:96757]	Up	8.604	0.270	0.000	60.500	**0.004***	**0.031***
Dvl1	dishevelled segment polarity protein 1 [Source:MGI Symbol;Acc:MGI:94941]	Up	0.951	5.848	670.200	2073.833	**0.004***	**0.032***

Ccl25	chemokine (C-C motif) ligand 25 [Source:MGI Symbol;Acc:MGI:1099448]	Down	-2.080	3.853	384.800	157.333	**0.006***	**0.043***
Polg+	polymerase (DNA directed), gamma [Source:MGI Symbol;Acc:MGI:1196389]	Up	1.284	4.762	273.000	1083.833	**0.007***	**0.046***
Mif	macrophage migration inhibitory factor (glycosylation-inhibiting factor) [Source:MGI Symbol;Acc:MGI:96982]	Up	0.965	6.443	1040.000	3291.333	**0.008***	0.051
Akt1+	thymoma viral proto-oncogene 1 [Source:MGI Symbol;Acc:MGI:87986]	Down	-0.792	7.221	3454.400	3160.833	**0.008***	0.051
Kitl	kit ligand [Source:MGI Symbol;Acc:MGI:96974]	Down	-1.398	4.718	688.200	448.667	**0.009***	0.053
Nudt1	nudix (nucleoside diphosphate linked moiety X)-type motif 1 [Source:MGI Symbol;Acc:MGI:109280]	Up	2.208	4.667	161.000	1168.500	**0.009***	0.054
Txn1	thioredoxin 1 [Source:MGI Symbol;Acc:MGI:98874]	Up	0.617	9.189	12493.600	14794.667	**0.012***	0.068
Atg5+	autophagy related 5 [Source:MGI Symbol;Acc:MGI:1277186]	Down	-1.175	5.376	1061.400	771.000	**0.015***	0.079
Xpa	xeroderma pigmentosum, complementation group A [Source:MGI Symbol;Acc:MGI:99135]	Down	-1.095	4.965	705.200	619.667	**0.015***	0.080
Gsk3a+	glycogen synthase kinase 3 alpha [Source:MGI Symbol;Acc:MGI:2152453]	Down	-1.144	4.932	677.200	544.667	**0.015***	0.081
Atm+	ataxia telangiectasia mutated [Source:MGI Symbol;Acc:MGI:107202]	Up	3.348	3.663	36.800	631.333	**0.018***	0.091
Stub1	STIP1 homology and U-Box containing protein 1 [Source:MGI Symbol;Acc:MGI:1891731]	Up	0.748	5.758	697.600	1991.500	**0.018***	0.092
Fzd5	frizzled class receptor 5 [Source:MGI Symbol;Acc:MGI:108571]	Up	1.119	5.734	506.000	1924.333	**0.019***	0.094
Pparg+	peroxisome proliferator activated receptor gamma [Source:MGI Symbol;Acc:MGI:97747]	Down	-0.764	6.451	1904.400	1830.833	**0.020***	0.098
Surf1+	surfeit gene 1 [Source:MGI Symbol;Acc:MGI:98443]	Up	0.617	6.214	997.800	2598.833	**0.020***	0.099
Irs1	insulin receptor substrate 1 [Source:MGI Symbol;Acc:MGI:99454]	Up	8.446	0.128	0.000	64.667	**0.021***	0.102
Bub3+	BUB3 mitotic checkpoint protein [Source:MGI Symbol;Acc:MGI:1343463]	Up	0.639	5.991	1300.400	1500.500	**0.026***	0.124
Il18	interleukin 18 [Source:MGI Symbol;Acc:MGI:107936]	Up	1.105	5.977	625.800	2910.667	**0.029***	0.134
Atr+	ataxia telangiectasia and Rad3 related [Source:MGI Symbol;Acc:MGI:108028]	Up	3.677	2.958	27.000	392.167	**0.043***	0.172

Tnfrsf11b	tumor necrosis factor receptor superfamily, member 11b (osteoprotegerin) [Source:MGI Symbol;Acc:MGI:109587]	Up	6.513	-1.535	0.000	10.667	0.153	0.392
Ctsb	cathepsin B [Source:MGI Symbol;Acc:MGI:88561]	Up	0.333	8.126	4160.800	9379.667	0.224	0.491

Gene ID	Gene Description and Accession Number	Regulation Up/Down	logFC	logCPM	15 Month	25 Month	p-value	q-value
Htra2^{+}	HtrA serine peptidase 2 [Source:MGI Symbol;Acc:MGI:1928676]	Down	-1.265	4.460	755.667	385.667	**0.001***	0.194
Myc^{+}	myelocytomatosis oncogene [Source:MGI Symbol;Acc:MGI:97250]	Up	0.956	4.501	404.167	830.500	**0.005***	0.330
Kitl	kit ligand [Source:MGI Symbol;Acc:MGI:96974]	Down	-0.975	4.512	742.000	448.667	**0.009***	0.382
Mt1	metallothionein 1 [Source:MGI Symbol;Acc:MGI:97171]	Up	0.793	8.446	7754.833	11532.167	**0.020***	0.500
Socs2^{+}	suppressor of cytokine signaling 2 [Source:MGI Symbol;Acc:MGI:1201787]	Up	0.510	6.363	1988.000	2663.833	**0.020***	0.500
Ctnnb1	catenin (cadherin associated protein), beta 1 [Source:MGI Symbol;Acc:MGI:88276]	Down	-0.626	9.246	18896.667	13110.500	**0.020***	0.502
Pparg^{+}	peroxisome proliferator activated receptor gamma [Source:MGI Symbol;Acc:MGI:97747]	Down	-0.736	6.495	3119.833	1830.833	**0.022***	0.520
Cdk7^{+}	cyclin-dependent kinase 7 [Source:MGI Symbol;Acc:MGI:102956]	Down	-0.630	4.549	790.000	546.667	**0.031***	0.578
Vwf	Von Willebrand factor [Source:MGI Symbol;Acc:MGI:98941]	Up	1.576	2.610	93.333	251.000	**0.038***	0.609
Sod2^{+}	superoxide dismutase 2, mitochondrial [Source:MGI Symbol;Acc:MGI:98352]	Down	-0.459	6.973	3992.833	3083.667	**0.041***	0.628
Ctsb	cathepsin B [Source:MGI Symbol;Acc:MGI:88561]	Down	-0.385	8.501	11455.833	9379.667	**0.042***	0.631
Msra^{+}	methionine sulfoxide reductase A [Source:MGI Symbol;Acc:MGI:106916]	Down	-0.453	6.188	2465.667	1742.333	**0.044***	0.634
Clock^{+}	circadian locomotor output cycles kaput [Source:MGI Symbol;Acc:MGI:99698]	Up	0.540	5.420	943.333	1506.833	**0.046***	0.639
Dvl1	dishevelled segment polarity protein 1 [Source:MGI Symbol;Acc:MGI:94941]	Up	0.763	5.898	1330.667	2073.833	0.052	0.660
Fzd5	frizzled class receptor 5 [Source:MGI Symbol;Acc:MGI:108571]	Down	-0.544	6.460	2977.333	1924.333	0.058	0.671
Dkkl1	dickkopf-like 1 [Source:MGI Symbol;Acc:MGI:1354963]	Down	-1.902	0.879	76.333	19.333	0.110	0.776

Fzd9	frizzled class receptor 9 [Source:MGI Symbol;Acc:MGI:1313278]	Up	1.460	2.375	78.167	212.667	0.138	0.830
Grn	granulin [Source:MGI Symbol;Acc:MGI:95832]	Down	-0.350	6.782	3778.167	2934.333	0.173	0.874
Il18	interleukin 18 [Source:MGI Symbol;Acc:MGI:107936]	Up	0.589	6.144	1654.500	2910.667	0.198	0.901
Wnt2b	wingless-type MMTV integration site family, member 2B [Source:MGI Symbol;Acc:MGI:1261834]	Up	2.028	-0.128	9.500	38.667	0.223	0.920
Igfbp5	insulin-like growth factor binding protein 5 [Source:MGI Symbol;Acc:MGI:96440]	Up	0.979	2.337	66.000	179.000	0.232	0.924
Mmp15	matrix metallopeptidase 15 [Source:MGI Symbol;Acc:MGI:109320]	Up	0.252	7.484	4668.833	5363.833	0.234	0.926
Mmp14	matrix metallopeptidase 14 (membrane-inserted) [Source:MGI Symbol;Acc:MGI:101900]	Up	0.360	7.067	3628.000	4426.000	0.284	0.956
Hsp90b1	heat shock protein 90, beta (Grp94), member 1 [Source:MGI Symbol;Acc:MGI:98817]	Down	-0.203	8.903	14506.500	13029.500	0.295	0.960
Lcn2	lipocalin 2 [Source:MGI Symbol;Acc:MGI:96757]	Up	1.429	0.577	18.500	60.500	0.443	0.995
Mmp7	matrix metallopeptidase 7 [Source:MGI Symbol;Acc:MGI:103189]	Up	0.762	1.770	70.500	133.667	0.519	1.000
Jund	jun D proto-oncogene [Source:MGI Symbol;Acc:MGI:96648]	Down	-0.129	8.243	9260.167	8096.667	0.552	1.000
Stub1	STIP1 homology and U-Box containing protein 1 [Source:MGI Symbol;Acc:MGI:1891731]	Down	-0.110	6.134	2082.500	1991.500	0.601	1.000
Nudt1	nudix (nucleoside diphosphate linked moiety X)-type motif 1 [Source:MGI Symbol;Acc:MGI:109280]	Up	0.133	5.259	1112.667	1168.500	0.650	1.000
Ghr	growth hormone receptor [Source:MGI Symbol;Acc:MGI:95708]	Up	0.405	2.577	114.667	228.500	0.663	1.000
Fzd4	frizzled class receptor 4 [Source:MGI Symbol;Acc:MGI:108520]	Up	0.409	1.414	74.000	79.333	0.716	1.000
Txn1	thioredoxin 1 [Source:MGI Symbol;Acc:MGI:98874]	Down	-0.079	8.944	14813.167	14794.667	0.756	1.000
Ccl25	chemokine (C-C motif) ligand 25 [Source:MGI Symbol;Acc:MGI:1099448]	Up	0.162	2.500	140.000	157.333	0.814	1.000
Irs1	insulin receptor substrate 1 [Source:MGI Symbol;Acc:MGI:99454]	Up	0.451	0.762	42.000	64.667	0.824	1.000
Mif	macrophage migration inhibitory factor (glycosylation-inhibiting factor) [Source:MGI Symbol;Acc:MGI:96982]	Down	-0.046	6.855	3629.333	3291.333	0.861	1.000

Hmgb1	high mobility group box 1 [Source:MGI Symbol;Acc:MGI:96113]	Up	0.015	8.060	7464.833	7656.167	0.929	1.000
Tnfrsf11b	tumor necrosis factor receptor superfamily, member 11b (osteoprotegerin) [Source:MGI Symbol;Acc:MGI:109587]	Down	-0.167	-0.927	14.500	10.667	0.936	1.000
Wnt6	wingless-type MMTV integration site family, member 6 [Source:MGI Symbol;Acc:MGI:98960]	0	0.009	0.529	35.333	35.333	1.000	1.000

[+] = Unique gene for this comparison only. Edge-R conducted to detect RNASeq differential gene expression levels between aging mice groups. Results corrected for multiple comparisons using the Benjamini-Hochberg method (q-value). *Significance: p-value < 0.05; q-value < 0.05. Mean gene expression levels per group displayed. Number of mice per group: 2 Month (n = 5), 15 Month (n = 6), and 25 Month (n = 6). logFC = log fold change; logCPM = log counts per million.

Supplementary Table 6. Inflammation Differentially Expressed Genes between Aging Mice Groups.

Gene ID	Gene Description and Accession Number	Regulation Up/Down	logFC	logCPM	2 Month	15 Month	p-value	q-value
Saa1	serum amyloid A 1 [Source:MGI Symbol;Acc:MGI:98221]	Down	-2.267	6.463	2680.400	854.500	**0.0001***	**0.008***
C3	complement component 3 [Source:MGI Symbol;Acc:MGI:88227]	Up	9.167	0.765	0.000	100.667	**0.0001***	**0.008***
Reg3b	regenerating islet-derived 3 beta [Source:MGI Symbol;Acc:MGI:97478]	Down	-2.366	7.163	4269.600	1660.500	**0.0002***	**0.010***
Ghr	growth hormone receptor [Source:MGI Symbol;Acc:MGI:95708]	Up	9.872	1.439	0.000	114.667	**0.0004***	**0.016***
C2cd4a	C2 calcium-dependent domain containing 4A [Source:MGI Symbol;Acc:MGI:3645763]	Up	8.464	0.109	0.000	61.667	**0.001***	**0.033***
Ptges	prostaglandin E synthase [Source:MGI Symbol;Acc:MGI:1927593]	Up	9.420	1.009	0.000	126.167	**0.003***	0.050
Nupr1	nuclear protein transcription regulator 1 [Source:MGI Symbol;Acc:MGI:1891834]	Up	1.231	9.317	6959.400	29340.833	**0.004***	0.066
Tgfb1	transforming growth factor, beta 1 [Source:MGI Symbol;Acc:MGI:98725]	Up	5.429	2.524	3.600	338.667	**0.006***	0.076
Fcer1g	Fc receptor, IgE, high affinity I, gamma polypeptide [Source:MGI Symbol;Acc:MGI:95496]	Up	9.329	0.910	0.000	58.167	**0.007***	0.083
Ahcy	S-adenosylhomocysteine hydrolase [Source:MGI Symbol;Acc:MGI:87968]	Up	0.658	8.311	6932.000	7672.833	**0.008***	0.090
Adora1	adenosine A1 receptor [Source:MGI Symbol;Acc:MGI:99401]	Up	8.145	-0.184	0.000	45.167	**0.008***	0.091
Lbp	lipopolysaccharide binding protein [Source:MGI Symbol;Acc:MGI:1098776]	Up	8.416	0.049	0.000	33.000	**0.010***	0.101
Nlrp6	NLR family, pyrin domain containing 6 [Source:MGI Symbol;Acc:MGI:2141990]	Up	0.685	7.795	2907.200	7820.167	**0.015***	0.127
Serpinb9	serine (or cysteine) peptidase inhibitor, clade B, member 9 [Source:MGI Symbol;Acc:MGI:106603]	Up	8.434	0.081	0.000	59.333	**0.018***	0.141
Plscr1	phospholipid scramblase 1 [Source:MGI Symbol;Acc:MGI:893575]	Down	-0.859	6.617	2512.200	2090.333	**0.020***	0.151
Il20rb	interleukin 20 receptor beta [Source:MGI Symbol;Acc:MGI:2143266]	Up	7.169	-1.028	0.000	23.667	**0.031***	0.198
Tnfrsf11a	tumor necrosis factor receptor superfamily, member 11a, NFKB activator [Source:MGI Symbol;Acc:MGI:1314891]	Up	0.994	5.566	468.600	1756.500	**0.035***	0.209

Adora2b	adenosine A2b receptor [Source:MGI Symbol;Acc:MGI:99403]	Up	6.425	-1.620	0.000	13.167	**0.046***	0.240
Ido1	indoleamine 2,3-dioxygenase 1 [Source:MGI Symbol;Acc:MGI:96416]	Down	-3.865	2.075	133.800	21.167	**0.050***	0.250
**Gene ID**	**Gene Description and Accession Number**	**Regulation Up/Down**	**logFC**	**logCPM**	**2 Month**	**25 Month**	**p-value**	**q-value**
C3	complement component 3 [Source:MGI Symbol;Acc:MGI:88227]	Up	10.092	1.688	0.000	152.833	**5.91E-08***	**3.88E-06***
Ahcy	S-adenosylhomocysteine hydrolase [Source:MGI Symbol;Acc:MGI:87968]	Down	-1.027	8.194	6932.000	5732.333	**3.27E-07***	**1.49E-05***
Ghr	growth hormone receptor [Source:MGI Symbol;Acc:MGI:95708]	Up	10.284	1.876	0.000	228.500	**6.91E-07***	**2.84E-05***
Ptges	prostaglandin E synthase [Source:MGI Symbol;Acc:MGI:1927593]	Up	8.959	0.600	0.000	66.333	**4.2E-06***	**0.0001***
Saa1	serum amyloid A 1 [Source:MGI Symbol;Acc:MGI:98221]	Down	-2.206	6.511	2680.400	1177.000	**7.07E-06***	**0.0001***
C2cd4a	C2 calcium-dependent domain containing 4A [Source:MGI Symbol;Acc:MGI:3645763]	Up	8.722	0.378	0.000	60.667	**1.03E-05***	**0.0002***
Dnase1^{+}	deoxyribonuclease I [Source:MGI Symbol;Acc:MGI:103157]	Up	8.011	-0.273	0.000	36.667	**1.96E-05***	**0.0004***
Nupr1	nuclear protein transcription regulator 1 [Source:MGI Symbol;Acc:MGI:1891834]	Up	1.312	9.412	6959.400	26337.000	**4.28E-05***	**0.0007***
Il20rb	interleukin 20 receptor beta [Source:MGI Symbol;Acc:MGI:2143266]	Up	8.477	0.153	0.000	63.833	**0.0004***	**0.005***
Reg3b	regenerating islet-derived 3 beta [Source:MGI Symbol;Acc:MGI:97478]	Down	-3.589	7.038	4269.600	619.500	**0.0004***	**0.006***
Adora2b	adenosine A2b receptor [Source:MGI Symbol;Acc:MGI:99403]	Up	8.353	0.039	0.000	48.667	**0.0006***	**0.007***
Selenos^{+}	selenoprotein S [Source:MGI Symbol;Acc:MGI:95994]	Up	0.951	6.707	1268.600	4089.500	**0.0008***	**0.009***
Tgfb1	transforming growth factor, beta 1 [Source:MGI Symbol;Acc:MGI:98725]	Up	5.731	2.870	3.600	371.667	**0.0009***	**0.010***
Plscr1	phospholipid scramblase 1 [Source:MGI Symbol;Acc:MGI:893575]	Down	-1.099	6.562	2512.200	1794.333	**0.001***	**0.010***
Fcer1g	Fc receptor, IgE, high affinity I, gamma polypeptide [Source:MGI Symbol;Acc:MGI:95496]	Up	7.260	-0.926	0.000	20.500	**0.001***	**0.012***
Tnfrsf11a	tumor necrosis factor receptor superfamily, member 11a, NFKB activator [Source:MGI Symbol;Acc:MGI:1314891]	Up	1.220	5.747	468.600	2139.167	**0.004***	**0.031***

Reg3g^{+}	regenerating islet-derived 3 gamma [Source:MGI Symbol;Acc:MGI:109406]	Down	-2.630	5.583	1460.400	374.833	**0.004***	**0.032***
Adora1	adenosine A1 receptor [Source:MGI Symbol;Acc:MGI:99401]	Up	8.342	0.025	0.000	42.333	**0.005***	**0.034***
Park7^{+}	Parkinson disease (autosomal recessive, early onset) 7 [Source:MGI Symbol;Acc:MGI:2135637]	Up	0.627	7.550	2543.600	6711.500	**0.007***	**0.046***
Serpinb9	serine (or cysteine) peptidase inhibitor, clade B, member 9 [Source:MGI Symbol;Acc:MGI:106603]	Up	7.605	-0.630	0.000	29.833	**0.007***	**0.048***
Tgfb2^{+}	transforming growth factor, beta 2 [Source:MGI Symbol;Acc:MGI:98726]	Up	7.783	-0.480	0.000	26.833	**0.008***	**0.049***
Akt1^{+}	thymoma viral proto-oncogene 1 [Source:MGI Symbol;Acc:MGI:87986]	Down	-0.792	7.221	3454.400	3160.833	**0.008***	0.051
Serpinf2^{+}	serine (or cysteine) peptidase inhibitor, clade F, member 2 [Source:MGI Symbol;Acc:MGI:107173]	Up	7.001	-1.143	0.000	16.167	**0.011***	0.061
Csf1^{+}	colony stimulating factor 1 (macrophage) [Source:MGI Symbol;Acc:MGI:1339753]	Up	7.319	-0.871	0.000	26.500	**0.011***	0.062
Pparg^{+}	peroxisome proliferator activated receptor gamma [Source:MGI Symbol;Acc:MGI:97747]	Down	-0.764	6.451	1904.400	1830.833	**0.020***	0.098
Sigirr^{+}	single immunoglobulin and toll-interleukin 1 receptor (TIR) domain [Source:MGI Symbol;Acc:MGI:1344402]	Up	2.094	5.180	294.200	1630.833	**0.024***	0.116
Pik3r1^{+}	phosphoinositide-3-kinase regulatory subunit 1 [Source:MGI Symbol;Acc:MGI:97583]	Up	0.860	5.929	715.200	2118.500	**0.040***	0.164
Lbp	lipopolysaccharide binding protein [Source:MGI Symbol;Acc:MGI:1098776]	Up	6.976	-1.159	0.000	17.333	**0.041***	0.168
Ffar2^{+}	free fatty acid receptor 2 [Source:MGI Symbol;Acc:MGI:2441731]	Up	1.744	6.069	547.600	2747.000	**0.044***	0.174
Ighg2b^{+}	immunoglobulin heavy constant gamma 2B [Source:MGI Symbol;Acc:MGI:96445]	Up	6.611	-1.437	0.000	18.167	**0.048***	0.185
Ido1	indoleamine 2,3-dioxygenase 1 [Source:MGI Symbol;Acc:MGI:96416]	Down	-2.354	2.276	133.800	37.500	0.143	0.381
Nlrp6	NLR family, pyrin domain containing 6 [Source:MGI Symbol;Acc:MGI:2141990]	Up	0.023	7.422	2907.200	4879.833	0.933	0.978
**Gene ID**	**Gene Description and Accession Number**	**Regulation Up/Down**	**logFC**	**logCPM**	**15 Month**	**25 Month**	**p-value**	**q-value**

Nlrp6	NLR family, pyrin domain containing 6 [Source:MGI Symbol;Acc:MGI:2141990]	Down	-0.690	7.842	7820.167	4879.833	**0.003***	0.270
Tgfb2+	transforming growth factor, beta 2 [Source:MGI Symbol;Acc:MGI:98726]	Up	8.069	-0.616	0.000	26.833	**0.003***	0.296
C2cd4b+	C2 calcium-dependent domain containing 4B [Source:MGI Symbol;Acc:MGI:1922947]	Up	2.052	3.465	132.833	467.167	**0.007***	0.348
Ext1+	exostosin glycosyltransferase 1 [Source:MGI Symbol;Acc:MGI:894663]	Up	0.700	5.275	883.833	1329.333	**0.009***	0.394
Alox5+	arachidonate 5-lipoxygenase [Source:MGI Symbol;Acc:MGI:87999]	Down	-1.948	3.015	313.667	84.667	**0.015***	0.458
Pparg+	peroxisome proliferator activated receptor gamma [Source:MGI Symbol;Acc:MGI:97747]	Down	-0.736	6.495	3119.833	1830.833	**0.022***	0.520
Selenos+	selenoprotein S [Source:MGI Symbol;Acc:MGI:95994]	Up	0.477	6.872	2854.833	4089.500	**0.022***	0.522
Pdgfa+	platelet derived growth factor, alpha [Source:MGI Symbol;Acc:MGI:97527]	Up	0.534	6.225	1884.000	2559.667	**0.026***	0.555
Dnase1l3+	deoxyribonuclease 1-like 3 [Source:MGI Symbol;Acc:MGI:1314633]	Down	-6.327	-2.028	10.333	0.000	**0.030***	0.573
Ahcy	S-adenosylhomocysteine hydrolase [Source:MGI Symbol;Acc:MGI:87968]	Down	-0.391	7.873	7672.833	5732.333	**0.037***	0.607
Reg3g+	regenerating islet-derived 3 gamma [Source:MGI Symbol;Acc:MGI:109406]	Down	-1.737	4.975	1385.167	374.833	**0.040***	0.627
Sod2+	superoxide dismutase 2, mitochondrial [Source:MGI Symbol;Acc:MGI:98352]	Down	-0.459	6.973	3992.833	3083.667	**0.041***	0.628
Saa3+	serum amyloid A 3 [Source:MGI Symbol;Acc:MGI:98223]	Down	-3.696	1.595	114.500	11.500	**0.042***	0.631
Fcer1g	Fc receptor, IgE, high affinity I, gamma polypeptide [Source:MGI Symbol;Acc:MGI:95496]	Down	-2.082	1.132	58.167	20.500	0.175	0.878
Reg3b	regenerating islet-derived 3 beta [Source:MGI Symbol;Acc:MGI:97478]	Down	-1.240	5.220	1660.500	619.500	0.182	0.888
C3	complement component 3 [Source:MGI Symbol;Acc:MGI:88227]	Up	0.897	2.186	100.667	152.833	0.190	0.899
Adora2b	adenosine A2b receptor [Source:MGI Symbol;Acc:MGI:99403]	Up	1.900	0.230	13.167	48.667	0.251	0.938
Plscr1	phospholipid scramblase 1 [Source:MGI Symbol;Acc:MGI:893575]	Down	-0.256	6.118	2090.333	1794.333	0.257	0.940
Ido1	indoleamine 2,3-dioxygenase 1 [Source:MGI Symbol;Acc:MGI:96416]	Up	1.473	0.279	21.167	37.500	0.409	0.990
Il20rb	interleukin 20 receptor beta [Source:MGI Symbol;Acc:MGI:2143266]	Up	1.296	0.501	23.667	63.833	0.426	0.991

Tnfrsf11a	tumor necrosis factor receptor superfamily, member 11a, NFKB activator [Source:MGI Symbol;Acc:MGI:1314891]	Up	0.194	6.113	1756.500	2139.167	0.428	0.991
Lbp	lipopolysaccharide binding protein [Source:MGI Symbol;Acc:MGI:1098776]	Down	-1.450	0.391	33.000	17.333	0.453	0.998
Serpinb9	serine (or cysteine) peptidase inhibitor, clade B, member 9 [Source:MGI Symbol;Acc:MGI:106603]	Down	-0.844	0.617	59.333	29.833	0.647	1.000
Ghr	growth hormone receptor [Source:MGI Symbol;Acc:MGI:95708]	Up	0.405	2.577	114.667	228.500	0.663	1.000
Ptges	prostaglandin E synthase [Source:MGI Symbol;Acc:MGI:1927593]	Down	-0.486	1.705	126.167	66.333	0.673	1.000
C2cd4a	C2 calcium-dependent domain containing 4A [Source:MGI Symbol;Acc:MGI:3645763]	Down	-0.234	1.111	61.667	60.667	0.822	1.000
Nupr1	nuclear protein transcription regulator 1 [Source:MGI Symbol;Acc:MGI:1891834]	Down	-0.060	9.863	29340.833	26337.000	0.871	1.000
Adora1	adenosine A1 receptor [Source:MGI Symbol;Acc:MGI:99401]	Down	-0.171	0.766	45.167	42.333	0.916	1.000
Saa1	serum amyloid A 1 [Source:MGI Symbol;Acc:MGI:98221]	Up	0.051	5.116	854.500	1177.000	0.932	1.000

$^{+}$ = Unique gene for this comparison only. Edge-R conducted to detect RNASeq differential gene expression levels between aging mice groups. Results corrected for multiple comparisons using the Benjamini-Hochberg method (q-value). *Significance: p-value < 0.05; q-value < 0.05. Mean gene expression levels per group displayed. Number of mice per group: 2 Month (n = 5), 15 Month (n = 6), and 25 Month (n = 6). logFC = log fold change; logCPM = log counts per million.

Supplementary Table 7. Tight junction protein zonula occludens-1 (ZO-1) in aging mice.				
**Kruskal-Wallis test: p-value = 0.0005*⁺**				
**Mice Comparisons**	**Mean ± (SD)**	**Mean ± (SD)**	**p-value**	**q-value**
2 Month vs. 15 Month	72.42 (53.96)	64.25 (26.59)	0.5582⁺	0.1954⁺
2 Month vs 25 Month	72.42 (53.96)	34.77 (23.38)	**0.0016*⁺**	**0.0008*⁺**
15 Month vs 25 Month	64.25 (26.59)	34.77 (23.38)	**0.0009*⁺**	**0.0008*⁺**

Based on Shapiro-Wilk test of normality, the non-parametric Kruskal-Wallis test (⁺) was conducted on tight junction protein zonula occludens-1 (ZO-1) in aging mice. Results corrected for multiple comparisons using the Benjamini-Hochberg method (q-value). *Significance: p-value < 0.05; q-value < 0.05. Mean index score and standard deviation (SD) displayed. Number of values per group: 2 Month (n = 31), 15 Month (n = 19), and 25 Month (n = 37).

Supplementary Table 8. Gamma-H2AX phosphorylation (γH2AX) DNA damage signaling scores of aging mice.				
**One-Way Analysis of Variance: $F_{(2, 11)}$ = 10.04, p = 0.0033***				
**Mice Comparisons**	**Mean ± (SD)**	**Mean ± (SD)**	**p-value**	**q-value**
2 Month vs. 15 Month	24.63 (9.82)	35.79 (11.69)	0.2782	0.0974
2 Month vs 25 Month	24.63 (9.82)	61.78 (18.36)	**0.0010***	**0.0011***
15 Month vs 25 Month	35.79 (11.69)	61.78 (18.36)	**0.0259***	**0.0136***

Based on Shapiro-Wilk test of normality, the parametric one-way analysis of variance (ANOVA) conducted on gamma-H2AX phosphorylation (γH2AX) DNA damage signaling scores in aging mice. Results corrected for multiple comparisons using the Benjamini-Hochberg method (q-value). *Significance: p-value < 0.05; q-value < 0.05. Mean index score and standard deviation (SD) displayed. Number of values per group: 2 Month (n = 6), 15 Month (n = 3), and 25 Month (n = 5).

2.1 Supplementary Figures

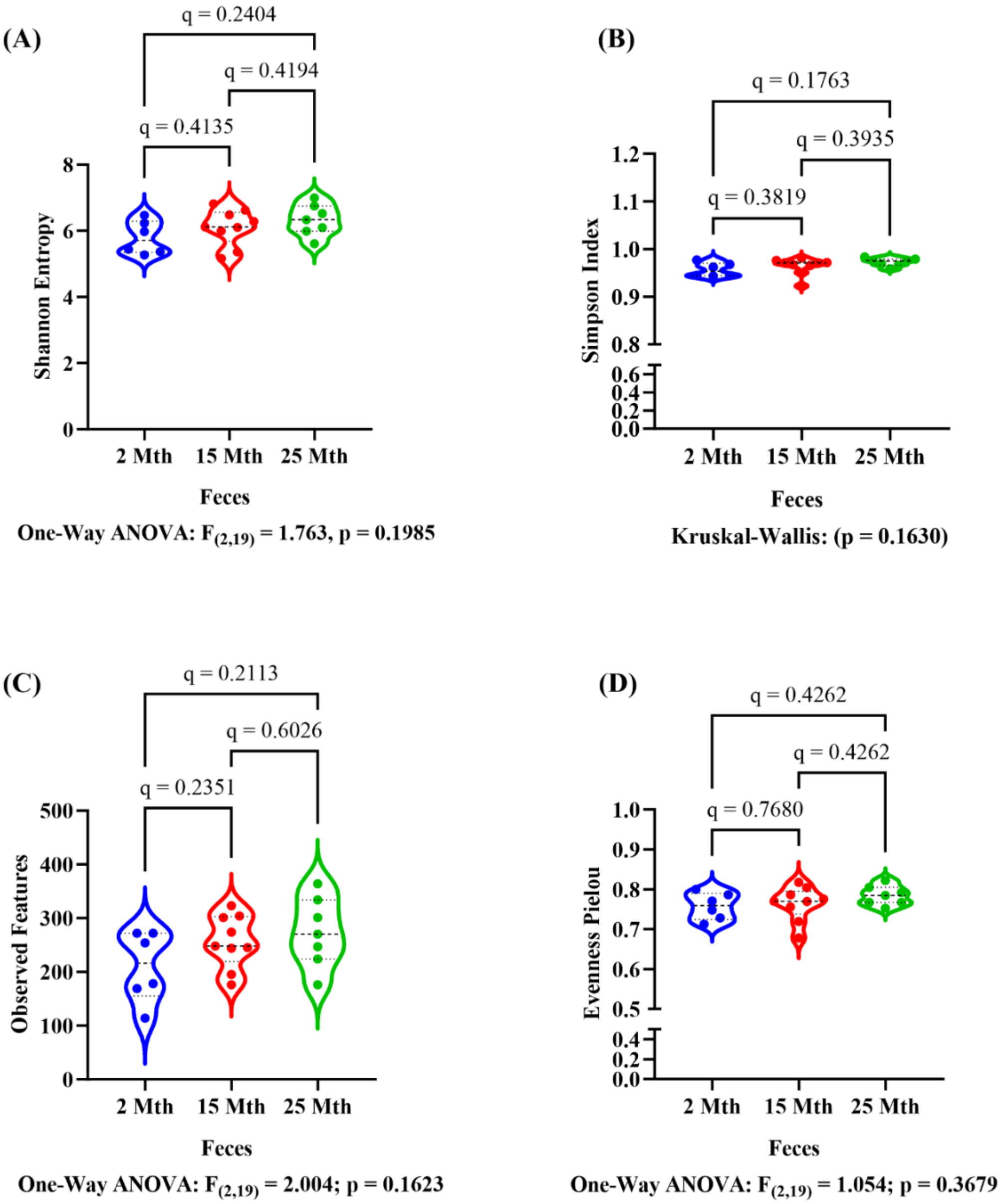

Supplementary Figure 1. Examination of Alpha-Diversity Indices within Gut Microbiota at Different Ages. Alpha diversity values of **(A)** Shannon's Index, **(B)** Simpson's Index, **(C)** Observed Features and **(D)** Pielou's Evenness examined at the feature level. Rarefaction level at 4,800 sequences per sample. Based on Shapiro-Wilk test of normality, either the parametric One-Way ANOVA or the non-parametric Kruskal-Wallis test were conducted within these alpha diversity indices, as shown within each figure. Results corrected for multiple comparisons using the Benjamini-Hochberg method (q-value). No significant differences in alpha-diversity indices were observed between age groups. Mth = Month. Group sample sizes: 2 Month (n = 6), 15 Month (n = 9), and 25 Month (n = 7).

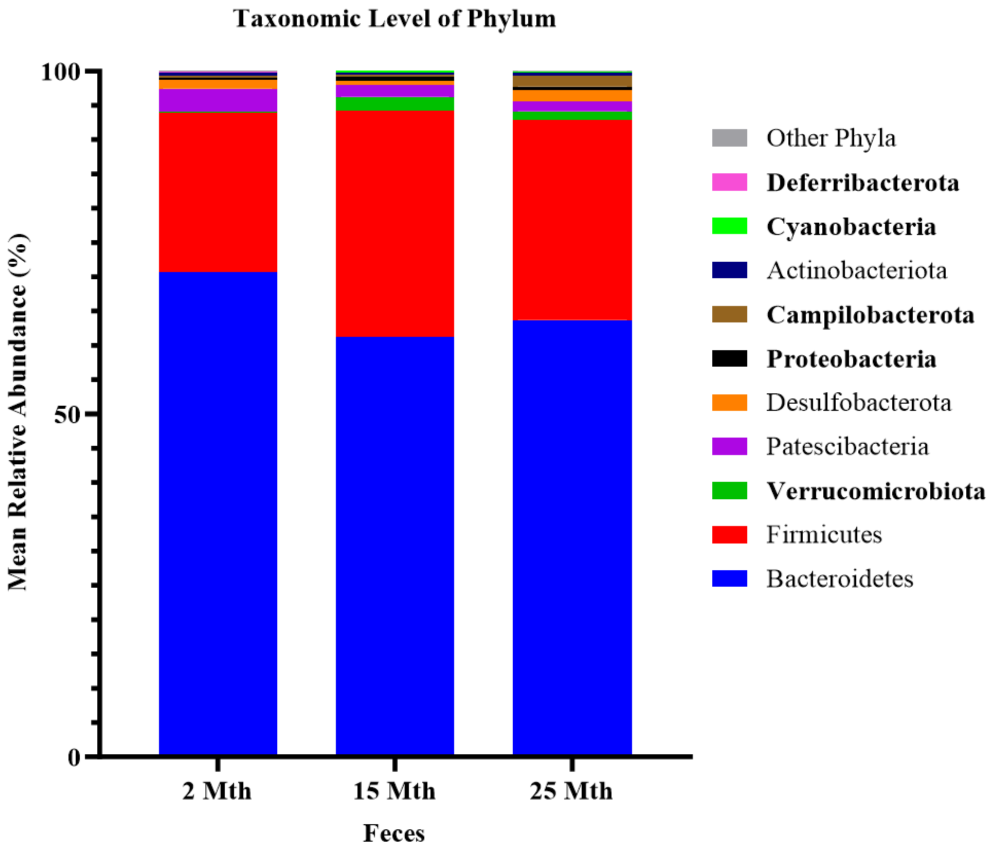

Supplementary Figure 2. Alteration in Gut Microbiota Composition at Different Ages. Mean relative abundance of microbial phyla (>1% relative abundance) for 2 Month (Mth), 15 Mth, and 25 Mth old mice. Bold taxa indicate a significant difference ($p < 0.05$) between aging mice groups assessed using the centered log-ratio with Kruskal-Wallis (CLR-KW) to generate p-values and corrected for multiple comparisons using the Benjamini-Hochberg method (q-value). Group sample sizes: 2 Month (n = 6), 15 Month (n = 9), and 25 Month (n = 7).

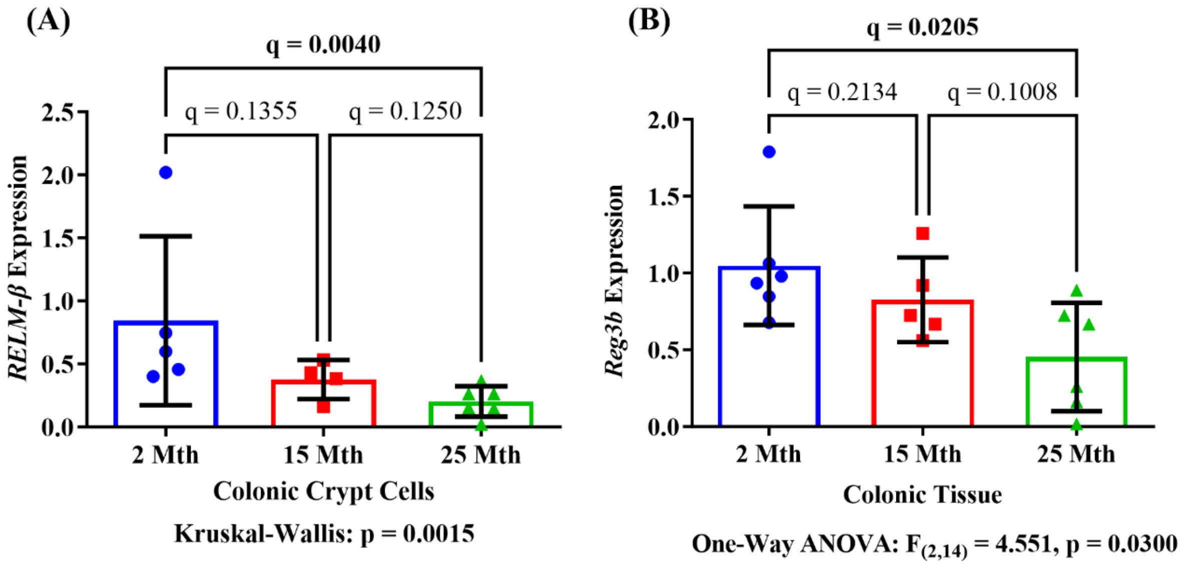

Supplementary Figure 3. Anti-Microbial Peptides *RELMβ* and *Reg3β* gene expression loss with aging mice validated with Real Time-Polymerase Chain Reaction on colonic tissue/crypt samples. Anti-microbial peptides **(A)** *RELMβ* and **(B)** *Reg3β* genes both demonstrated an aging effect indicating that these two gene expressions down-regulated with aging. Multiple comparison analysis showed significantly decreased expression of both RELMβ (*Retnlb*) (q = 0.0040) and *Reg3β* (q = 0.0205) in 25-month-old mice compared to 2-month-old mice. Based on Shapiro-Wilk test of normality, either the parametric One-Way ANOVA or the non-parametric Kruskal-Wallis test were conducted for differences between ages, as shown within each figure. Results corrected for multiple comparisons using the Benjamini-Hochberg method (q-value). Mth = Month.

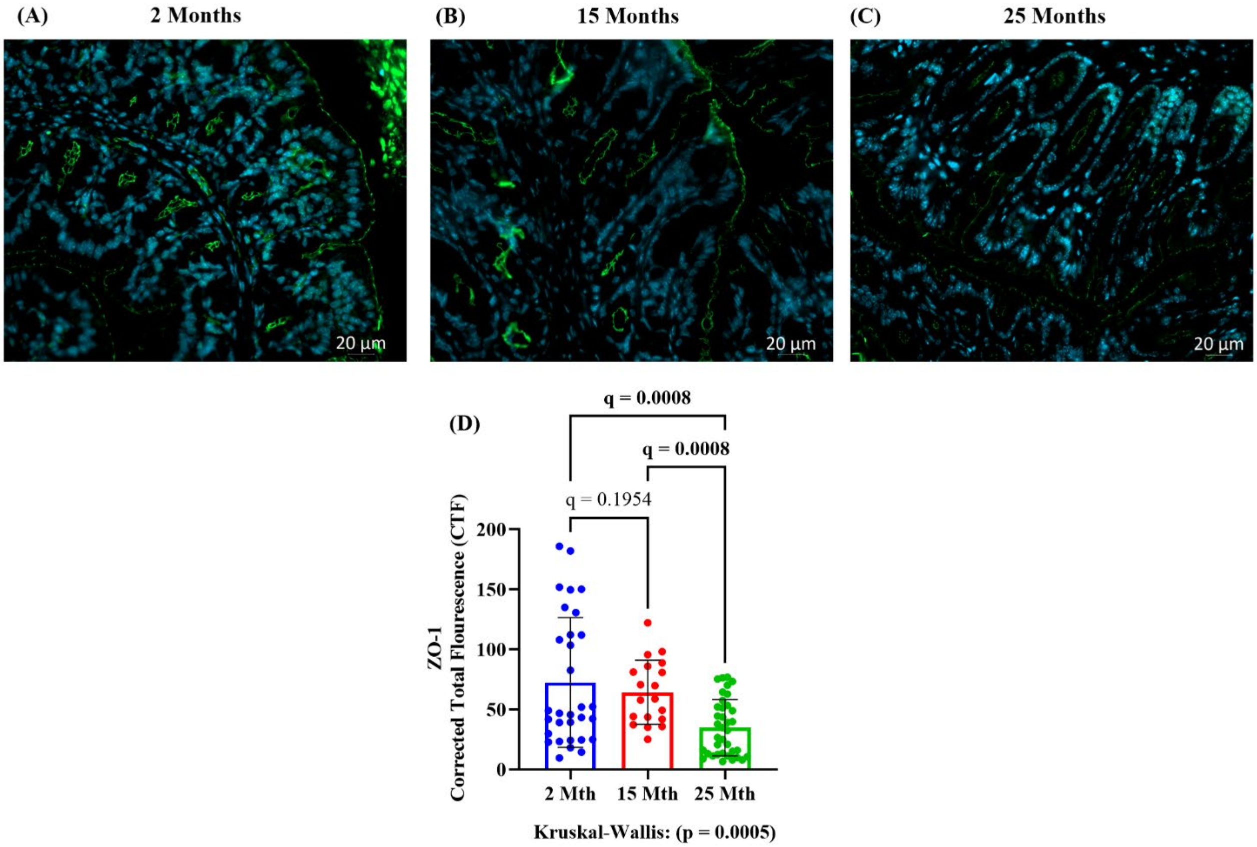

Supplementary Figure 4. Tight Junction Protein Zonula Occludens-1 Integrity in Aging Mice. **(A, B, C, D)** Immunofluorescence staining for ZO-1 demonstrated a significant (KW: p = 0.0005) disruption between aged mice groups **(A, B, C)**, indicating that its expression decreased with aging. **(D)** Multiple comparison analysis showed significantly (q = 0.0008) decreased expression of ZO-1 in 25-month-old mice, when compared to both 2-month- and 15-month-old mice groups. Based on Shapiro-Wilk test of normality, the non-parametric Kruskal-Wallis test was used to assess for differences between ages. Results corrected for multiple comparisons using the Benjamini-Hochberg method (q-value). Mth = Month.

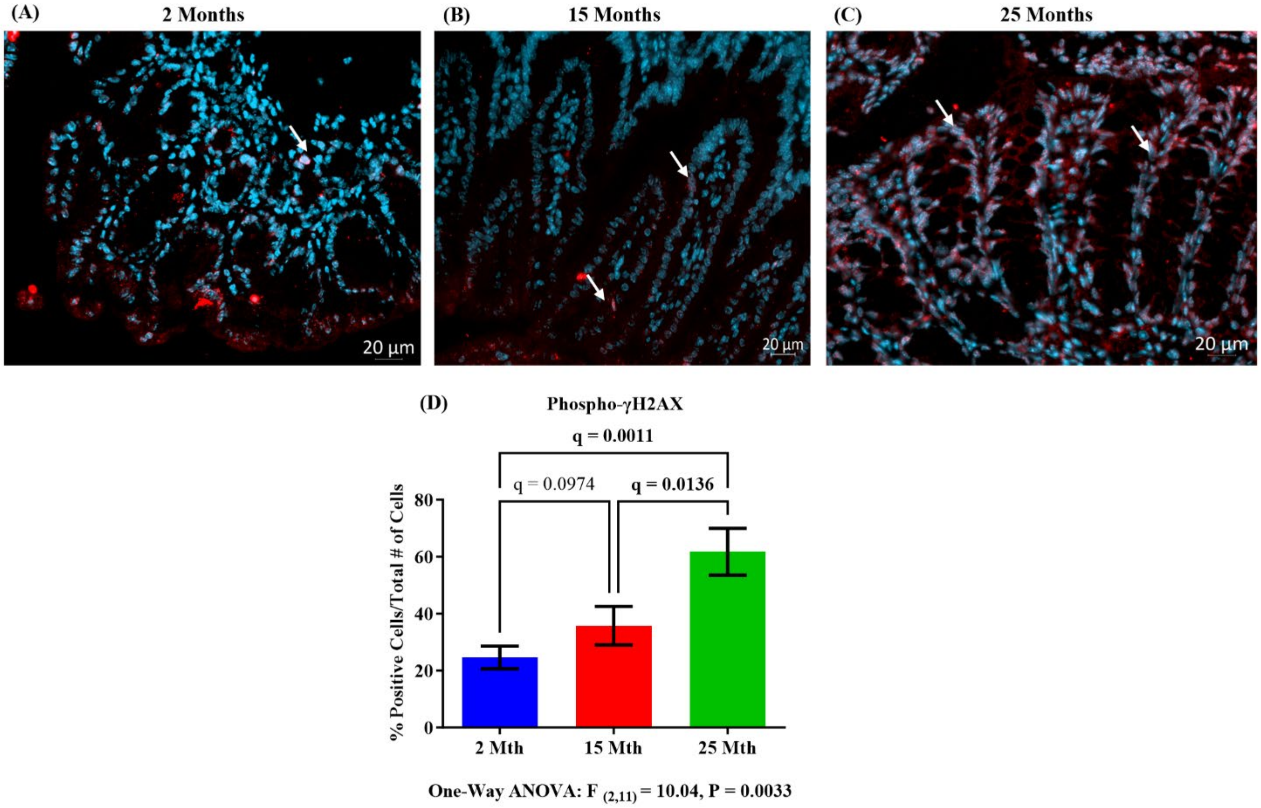

Supplementary Figure 5. Phosphorylation of the Histone Variant H2AX in Aging Mice. (A, B, C, D) The γH2AX protein expression demonstrated an aging effect (one-way ANOVA: $F_{(2,11)} = 10.04$, $p = 0.0033$), indicating that its expression increased with aging. **(D)** Multiple comparison analysis showed significantly increased expression of γH2AX in 25-month-old mice, when compared to both 2-month ($q = 0.0011$) and 15-month ($q = 0.0136$) -old mice groups. Based on Shapiro-Wilk test of normality, the parametric one-way analysis of variance (ANOVA) is used to assess differences between ages. Results corrected for multiple comparisons using the Benjamini-Hochberg method (q-value). Mth = Month.

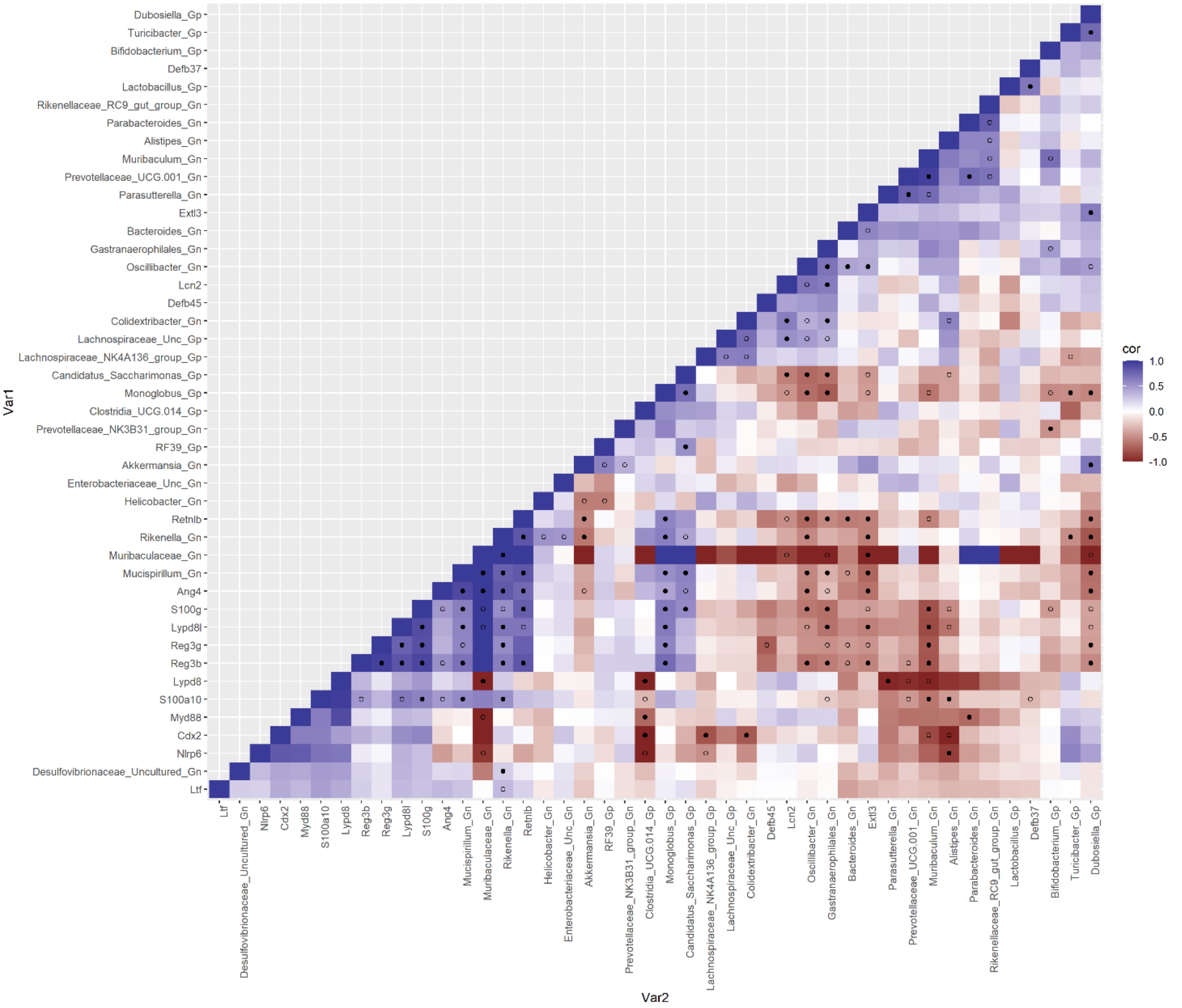

Supplementary Figure 6. Interactions between Aging Anti-Microbial Peptides Genes and Gram-Positive/Gram-Negative Gut Microbes comparing Two-Month-Old and 15-Month-Old Mice. Sparse Correlation for Compositional Data (SparCC) plot depicting gene-microbe correlations. The color of the squares indicates the magnitude of the correlation. *Pseudo* p-values computed using one hundred randomized sets and then corrected using the Benjamini-Hochberg method. Heatmap indicates significant associations with p-values ($p<0.05$) empty circle and q-values ($q<0.05$) black circle. Gp = Gram-positive genus; Gn = Gram-negative genus.

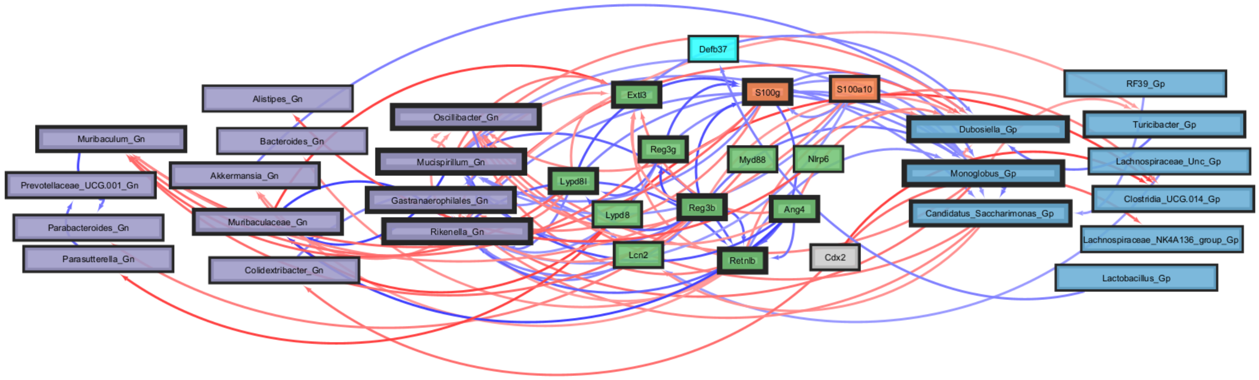

Supplementary Figure 7. Network Interactions Depicting Significant Aging Anti-Microbial Peptides Genes and Gram-Positive/Gram-Negative Gut Microbes comparing Two-Month-Old and 15-Month-Old Mice. Significant correlations ($R < -0.5$, $R > 0.5$; $q < 0.05$) generated from SparCC exported and visualized as network plots within the open-source platform Cytoscape 3.10.0. Blue edges indicate positive correlation and red edges indicate negative correlation. Purple nodes indicate Gram-negative genera and blue nodes indicate Gram-positive genera. The colors for the genes and gene clusters (based on Markov cluster algorithm) are defined using interaction networks in STRING database v12.0. The thickness of the nodes or individual features calculated using the cytoHubba module within Cytoscape 3.10.0 and ranked by importance (thick black square: higher importance, thin black square: lower importance) by the Maximal Clique Centrality method.

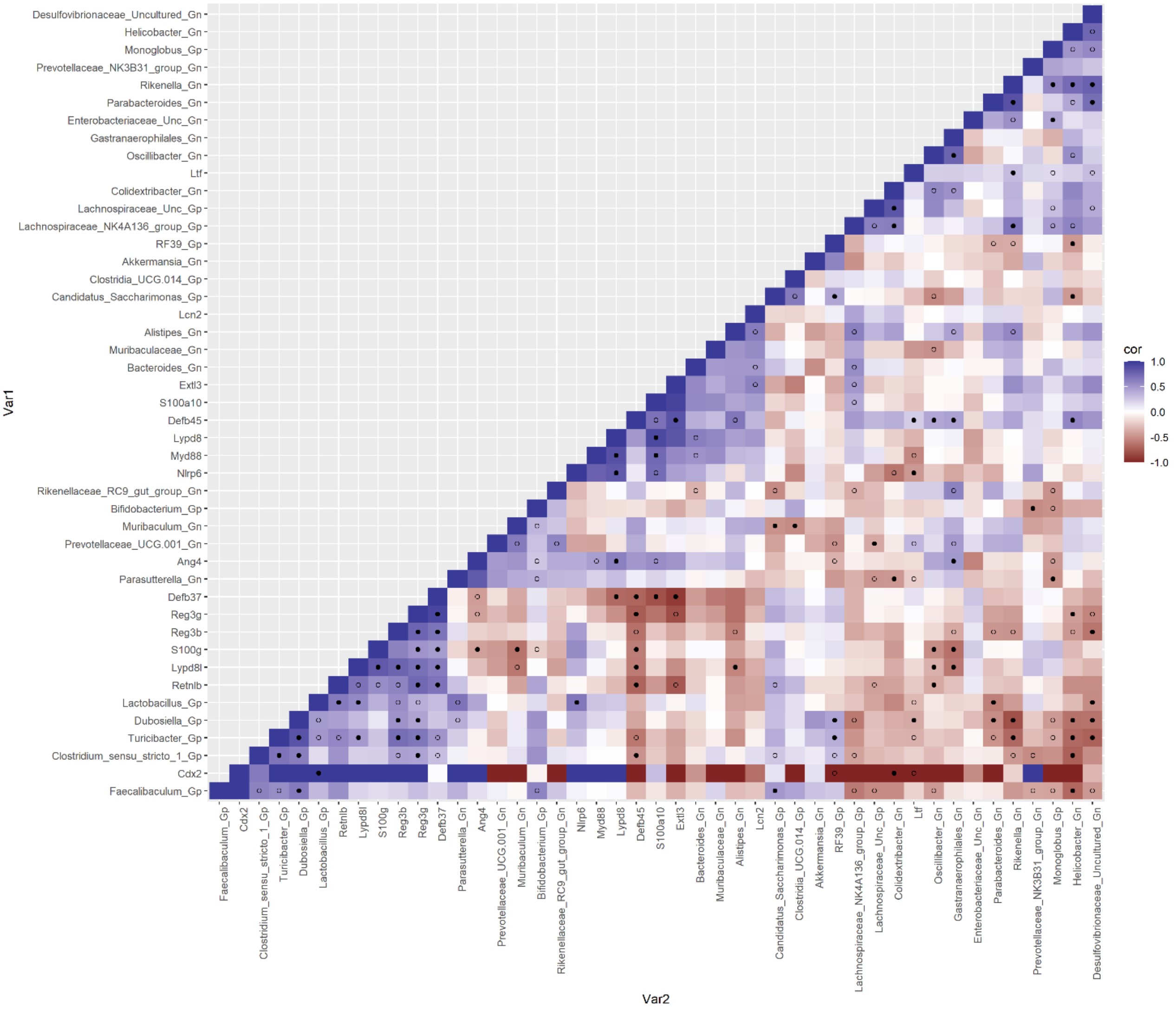

Supplementary Figure 8. Interactions between Aging Anti-Microbial Peptides Genes and Gram-Positive/Gram-Negative Gut Microbes comparing 15-Month-Old and 25-Month-Old Mice. Sparse Correlation for Compositional Data (SparCC) plot depicting gene-microbe correlations. The color of the squares indicates the magnitude of the correlation. *Pseudo* p-values computed using one hundred randomized sets and then corrected using the Benjamini-Hochberg method. Heatmap indicates significant associations with p-values ($p<0.05$) empty circle and q-values ($q<0.05$) black circle. Gp = Gram-positive genus; Gn = Gram-negative genus.

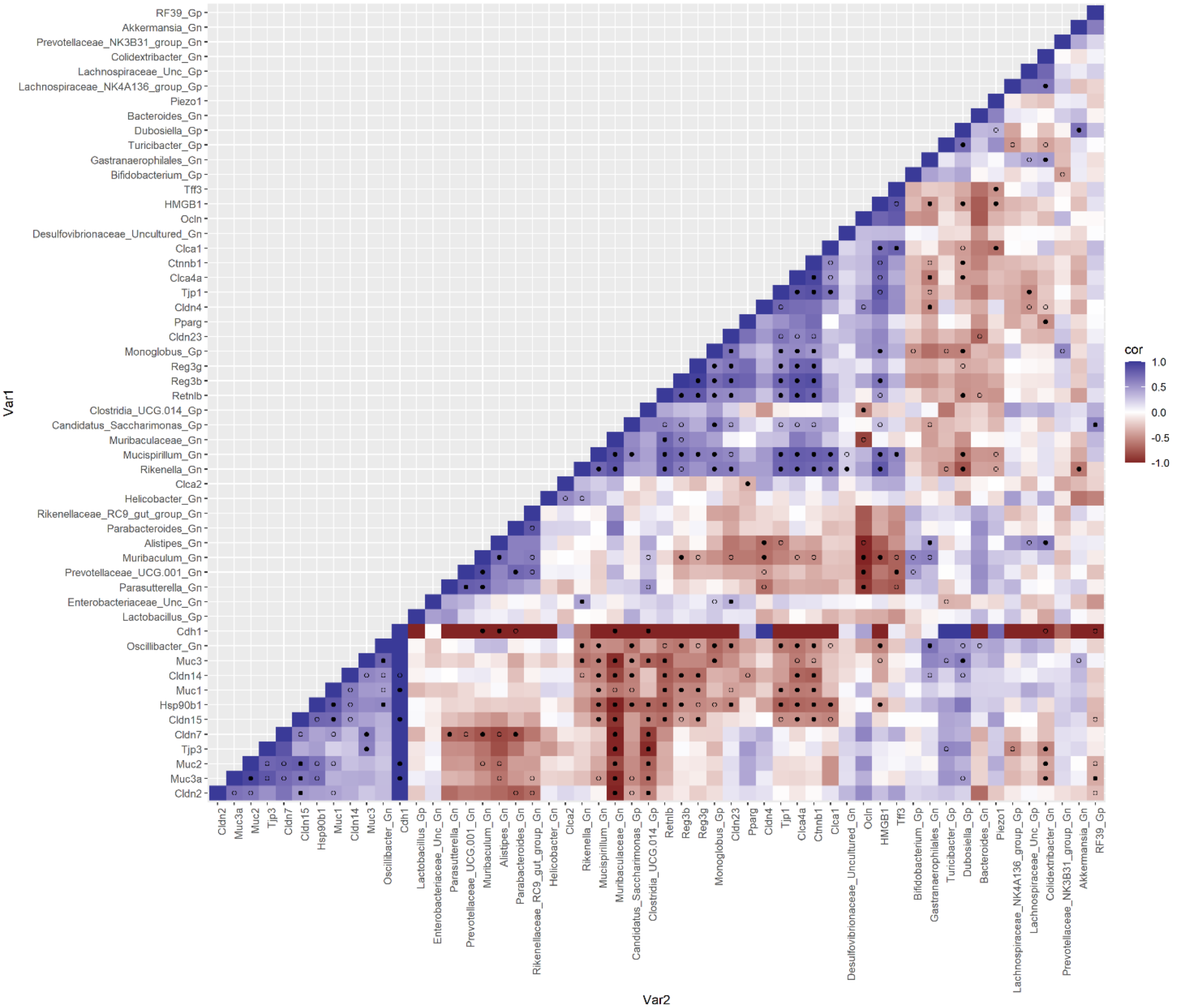

Supplementary Figure 9. Interactions between Aging Barrier Genes and Gram-Positive/Gram-Negative Gut Microbes comparing Two-Month-Old and 15-Month-Old Mice. Sparse Correlation for Compositional Data (SparCC) plot depicting gene-microbe correlations. The color of the squares indicates the magnitude of the correlation. *Pseudo* p-values computed using one hundred randomized sets and then corrected using the Benjamini-Hochberg method. Heatmap indicates significant associations with p-values ($p<0.05$) empty circle and q-values ($q<0.05$) black circle. Gp = Gram-positive genus; Gn = Gram-negative genus.

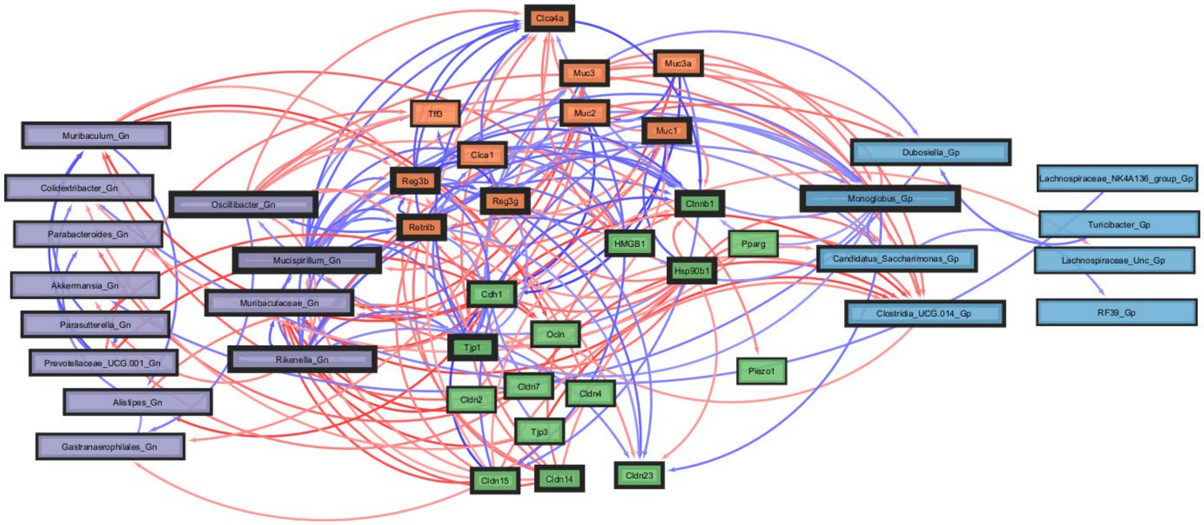

Supplementary Figure 10. Network Interactions Depicting Significant Aging Barrier Genes and Gram-Positive/Gram-Negative Gut Microbes comparing Two-Month-Old and 15-Month-Old Mice. Significant correlations ($R < -0.5$, $R > 0.5$; $q < 0.05$) generated from SparCC exported and visualized as network plots within the open-source platform Cytoscape 3.10.0. Blue edges indicate positive correlation and red edges indicate negative correlation. Purple nodes indicate Gram-negative genera and blue nodes indicate Gram-positive genera. The colors for the genes and gene clusters (based on Markov cluster algorithm) are defined using interaction networks in STRING database v12.0. The thickness of the nodes or individual features calculated using the cytoHubba module within Cytoscape 3.10.0 and ranked by importance (thick black square: higher importance, thin black square: lower importance) by the Maximal Clique Centrality method.

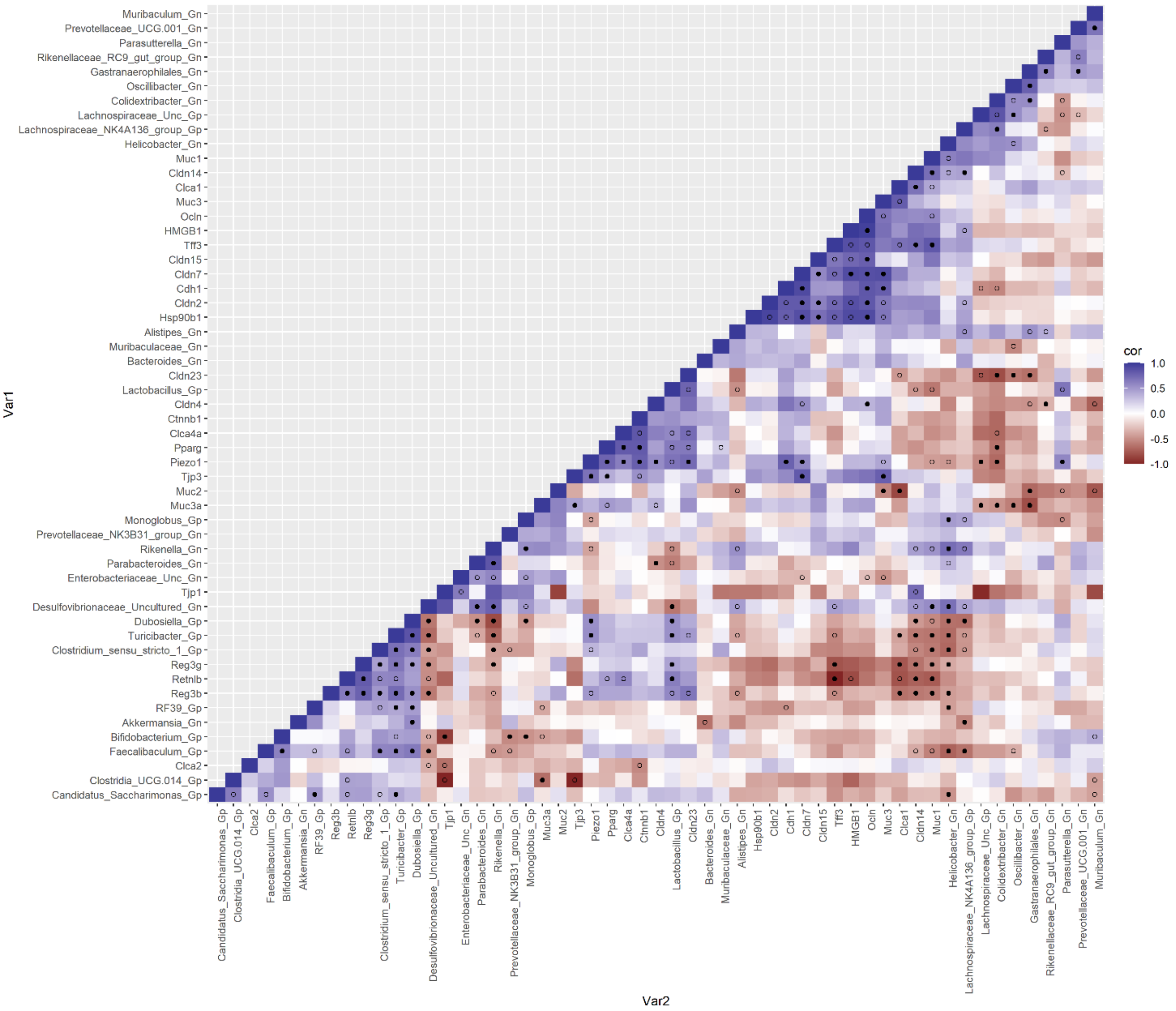

Supplementary Figure 11. Interactions between Aging Barrier Genes and Gram-Positive/Gram-Negative Gut Microbes comparing 15-Month-Old and 25-Month-Old Mice. Sparse Correlation for Compositional Data (SparCC) plot depicting gene-microbe correlations. The color of the squares indicates the magnitude of the correlation. *Pseudo* p-values computed using one hundred randomized sets and then corrected using the Benjamini-Hochberg method. Heatmap indicates significant associations with p-values (p<0.05) empty circle and q-values (q<0.05) black circle. Gp = Gram-positive genus; Gn = Gram-negative genus.

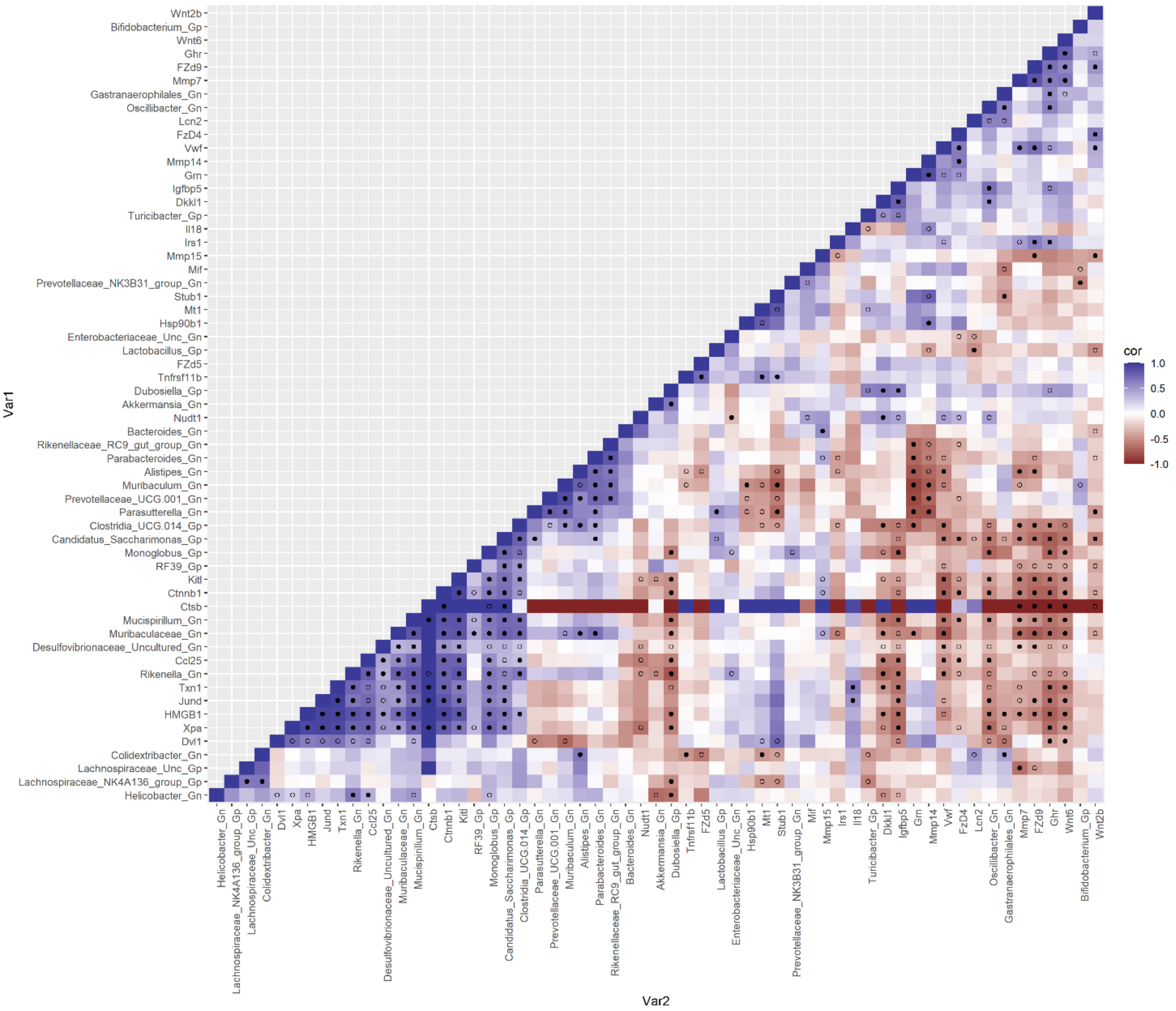

Supplementary Figure 12. Interactions between Aging Senescence Genes and Gram-Positive/Gram-Negative Gut Microbes comparing Two-Month-Old and 15-Month-Old Mice. Sparse Correlation for Compositional Data (SparCC) plot depicting gene-microbe correlations. The color of the squares indicates the magnitude of the correlation. *Pseudo* p-values computed using one hundred randomized sets and then corrected using the Benjamini-Hochberg method. Heatmap indicates significant associations with p-values (p<0.05) empty circle and q-values (q<0.05) black circle. Gp = Gram-positive genus; Gn = Gram-negative genus.

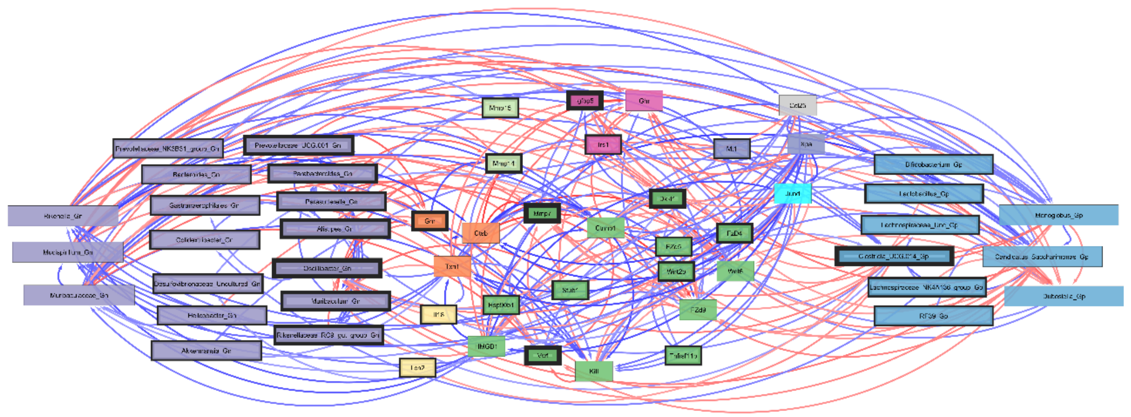

Supplementary Figure 13. Network Interactions Depicting Significant Aging Senescence Genes and Gram-Positive/Gram-Negative Gut Microbes comparing Two-Month-Old and 15-Month-Old Mice. Significant correlations ($R < -0.5$, $R > 0.5$; $q < 0.05$) generated from SparCC exported and visualized as network plots within the open-source platform Cytoscape 3.10.0. Blue edges indicate positive correlation and red edges indicate negative correlation. Purple nodes indicate Gram-negative genera and blue nodes indicate Gram-positive genera. The colors for the genes and gene clusters (based on Markov cluster algorithm) are defined using interaction networks in STRING database v12.0. The thickness of the nodes or individual features calculated using the cytoHubba module within Cytoscape 3.10.0 and ranked by importance (thick black square: higher importance, thin black square: lower importance) by the Maximal Clique Centrality method.

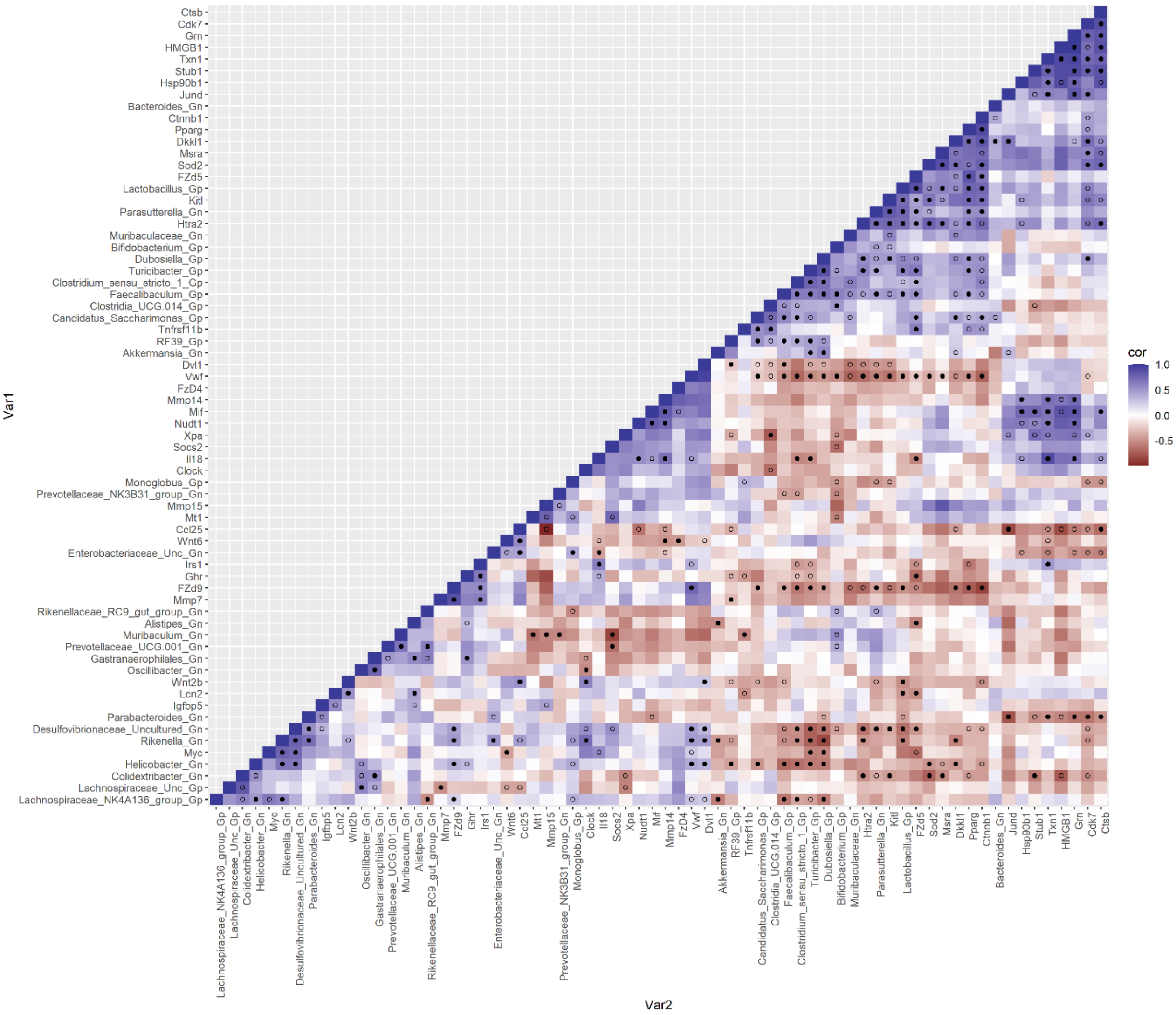

Supplementary Figure 14. Interactions between Aging Senescence Genes and Gram-Positive/Gram-Negative Gut Microbes comparing 15-Month-Old and 25-Month-Old Mice. Sparse Correlation for Compositional Data (SparCC) plot depicting gene-microbe correlations. The color of the squares indicates the magnitude of the correlation. *Pseudo* p-values computed using one hundred randomized sets and then corrected using the Benjamini-Hochberg method. Heatmap indicates significant associations with p-values ($p<0.05$) empty circle and q-values ($q<0.05$) black circle. Gp = Gram-positive genus; Gn = Gram-negative genus.

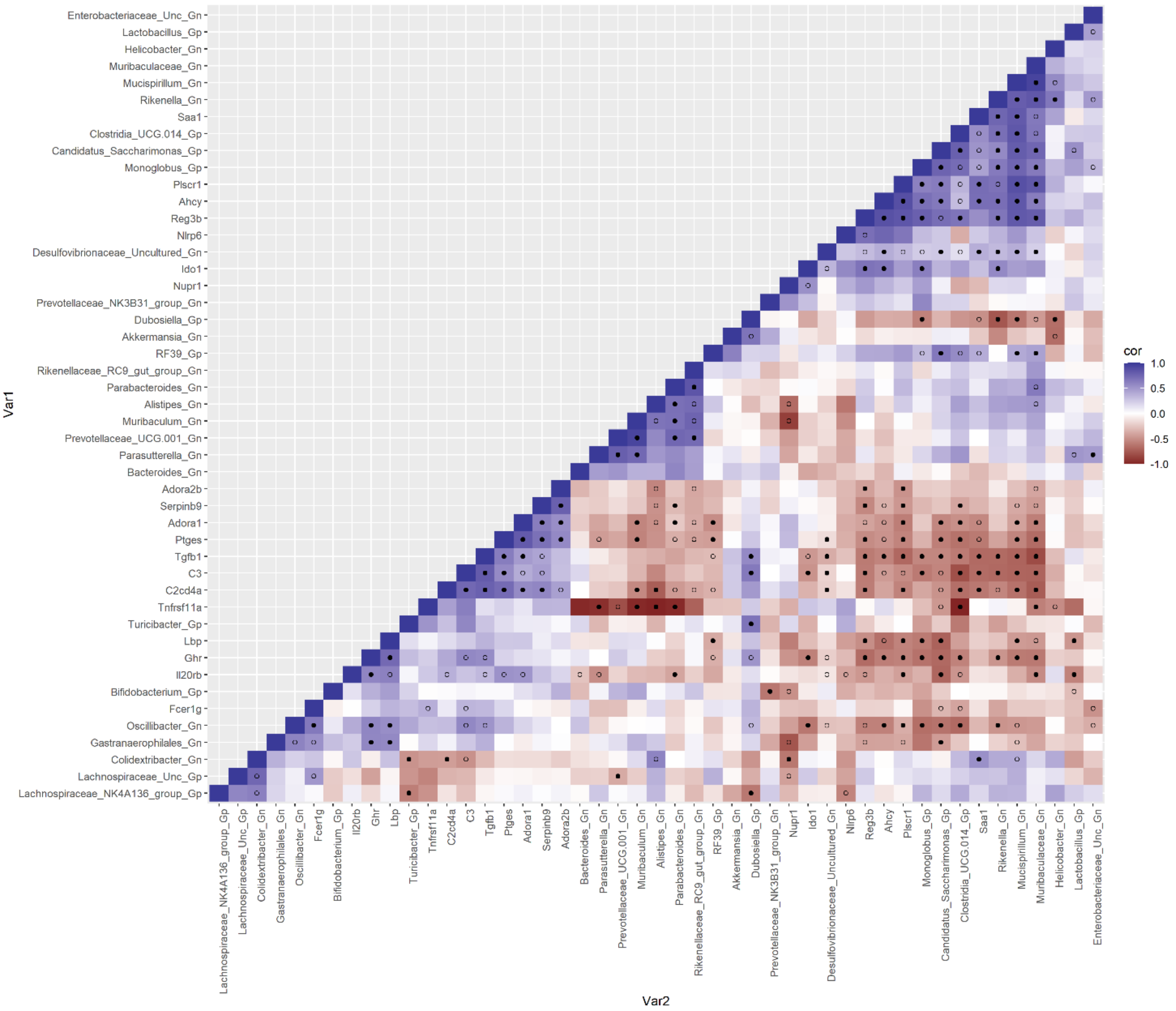

Supplementary Figure 15. Interactions between Aging Inflammation Genes and Gram-Positive/Gram-Negative Gut Microbes comparing Two-Month-Old and 15-Month-Old Mice. Sparse Correlation for Compositional Data (SparCC) plot depicting gene-microbe correlations. The color of the squares indicates the magnitude of the correlation. *Pseudo* p-values computed using one hundred randomized sets and then corrected using the Benjamini-Hochberg method. Heatmap indicates significant associations with p-values ($p<0.05$) empty circle and q-values ($q<0.05$) black circle. Gp = Gram-positive genus; Gn = Gram-negative genus.

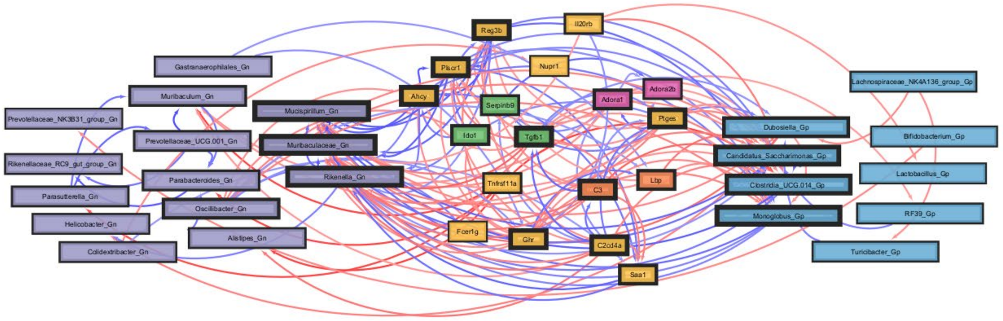

Supplementary Figure 16. Network Interactions Depicting Significant Aging Inflammation Genes and Gram-Positive/Gram-Negative Gut Microbes comparing Two-Month-Old and 15-Month-Old Mice. Significant correlations ($R < -0.5$, $R > 0.5$; $q < 0.05$) generated from SparCC exported and visualized as network plots within the open-source platform Cytoscape 3.10.0. Blue edges indicate positive correlation and red edges indicate negative correlation. Purple nodes indicate Gram-negative genera and blue nodes indicate Gram-positive genera. The colors for the genes and gene clusters (based on Markov cluster algorithm) are defined using interaction networks in STRING database v12.0. The thickness of the nodes or individual features calculated using the cytoHubba module within Cytoscape 3.10.0 and ranked by importance (thick black square: higher importance, thin black square: lower importance) by the Maximal Clique Centrality method.

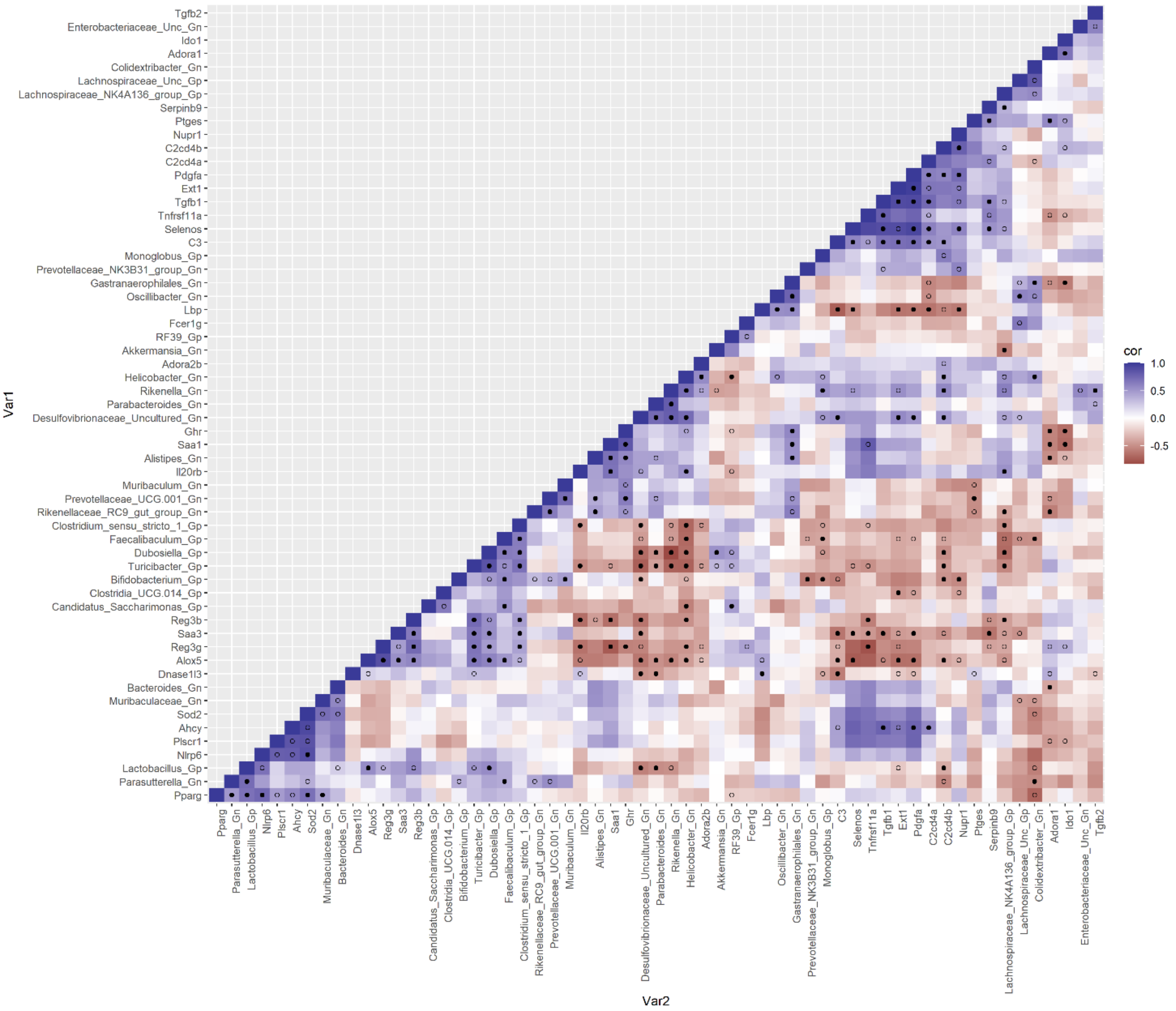

Supplementary Figure 17. Interactions between Aging Inflammation Genes and Gram-Positive/Gram-Negative Gut Microbes comparing 15-Month-Old and 25-Month-Old Mice. Sparse Correlation for Compositional Data (SparCC) plot depicting gene-microbe correlations. The color of the squares indicates the magnitude of the correlation. *Pseudo* p-values computed using one hundred randomized sets and then corrected using the Benjamini-Hochberg method. Heatmap indicates significant associations with p-values ($p<0.05$) empty circle and q-values ($q<0.05$) black circle. Gp = Gram-positive genus; Gn = Gram-negative genus.
